# SAR of Novel 3-Arylisoquinolinones: *meta*-Substitution on the Aryl Ring Dramatically Enhances Antiproliferative Activity through Binding to Microtubules

**DOI:** 10.1021/acs.jmedchem.1c01936

**Published:** 2022-03-15

**Authors:** Mai A. Elhemely, Asma A. Belgath, Sherihan El-Sayed, Kepa K. Burusco, Manikandan Kadirvel, Annalisa Tirella, Katherine Finegan, Richard A. Bryce, Ian J. Stratford, Sally Freeman

**Affiliations:** † Division of Pharmacy & Optometry, School of Health Sciences, Faculty of Biology, Medicine & Health, University of Manchester, Manchester M13 9PT, U.K.; ‡ Department of Pharmacology and Toxicology, Faculty of Pharmacy, Beni-Suef University, Beni-Suef 62514, Egypt; § Department of Medicinal Chemistry, Faculty of Pharmacy, Zagazig University, Zagazig 44519, Egypt; ∥ BIOtech Center for Biomedical Technologies, Department of Industrial Engineering, University of Trento, Via delle Regole 101, Trento 38123, Italy

## Abstract

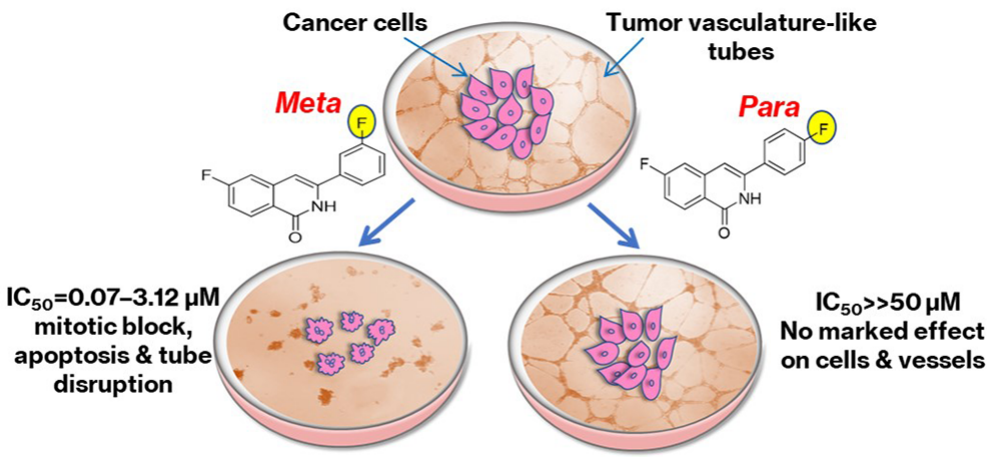

A set of *meta*-substituted 3-arylisoquinolinones have been identified that show substantial cytotoxicity in breast, liver, lung and colon cancer cell lines; these are up to 700-fold more active than the corresponding *para* analogues. These compounds were initially proposed as inhibitors of *N*-ribosyl dihydronicotinamide (NRH): quinone oxidoreductase 2 (NQO2) but were found to be inactive against the enzyme. Instead, COMPARE analysis suggested that 6-fluoro-3-(*meta*-fluorophenyl)isoquinolin-1(2*H*)-one (**4**) could mimic colchicine and interact with microtubules, a recognized target for cancer therapy. Subsequent docking, molecular dynamics simulations, and free energy analysis further suggested that compound **4** bound well into the colchicine-binding pocket of tubulin. Indeed, **4** suppressed tubulin polymerization, caused *G*_2_/*M* cell cycle arrest, and induced apoptosis. Also, **4** inhibited the formation of endothelial cell capillary-like tubes and further disrupted the structure of preestablished tubes; the effects were not observed with *para* analogue **5**. In accordance with this, the computed free energy of binding of **5** to tubulin was lower in magnitude than that for **4** and appeared to arise in part from the inability of the *para* substituent to occupy a tubulin subpocket, which is possible in the *meta* orientation. In conclusion, the antiproliferative potential of the novel 3-arylisoquinolinones is markedly influenced by a subtle change in the structure (*meta* versus *para*). The *meta*-substituted isoquinolinone **4** is a microtubule-destabilizing agent with potential tumor-selectivity and antiangiogenic and vascular disrupting features.

## Introduction

*N*-Ribosyl dihydronicotinamide (NRH): quinone oxidoreductase 2 (NQO2) is a cytosolic flavoprotein that catalyzes two-electron reduction of numerous quinones into hydroquinones, thereby deterring the production of one-electron reduced semiquinone radicals identified to trigger oxidative stress.^1^ Besides its presence in several normal tissues,^2^ NQO2 is overexpressed in different solid tumors such as colon, liver, lung, and breast cancers.^3^ There is evidence that NQO2 plays a protective role against cancer initiation; NQO2 downregulation in mice has been found to enhance the development of myeloid hyperplasia of bone marrow and the carcinogen-induction of skin cancer.^4,5^ In addition, genetic silencing of NQO2 in cancer cells has been associated with a reduction in the activity of the oncogenic NF-κB, thereby contributing against cancer progression.^6^ Further, it has been demonstrated that genetic or pharmacologic inhibition of NQO2 reduces cell proliferation in triple-negative breast and prostate cancer cell lines.^7,8^ Collectively, these results strongly suggest NQO2 to be a potential cancer therapeutic target.

**Figure 1:**
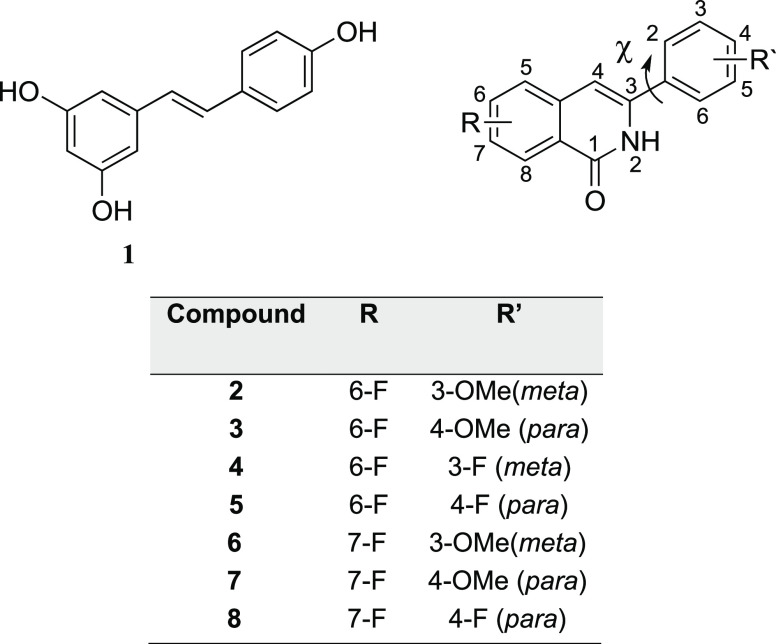
3-Arylisoquinolinones 2–8 as analogues of resveratrol 1. Angle χ is defined by atoms N_2_–C_3_–C_1_–C_2_.

For these reasons, we and others have sought to develop pharmacologically acceptable inhibitors of NQO2.^6,9−13^ The synthesis and biological evaluation of a series of 3-arylisoquinolinones are reported here. The rationale for choosing this series was based on their structural similarity to resveratrol **1** (Figure 1), which is the best-known inhibitor of NQO2.^14^ However, resveratrol is rapidly and extensively metabolized through both hepatic 3-*O*-glucuronidation and 3-*O*-sulfation of its phenolic groups and intestinal reductive metabolism of the stilbene bond resulting in poor oral bioavailability.^15,16^ In an attempt to overcome these problems, resveratrol’s phenolic groups were replaced with hydrogen (−H), methoxy (−OMe), or fluoro (−F) substituents, and the stilbene bond was incorporated into an isoquinolinone moiety to ultimately give our novel 3-arylisoquinolinones **2**–**8** (Figure 1). Structurally, the compounds can be classified into two main groups depending on the position of −OMe or −F substituents on the aryl ring: the *meta* compounds (**2**, **4**, **6**) and the *para* compounds (**3**, **5**, **7**, **8**).

Interestingly, isoquinolinones have been reported to exert antiproliferative activity against lung, ovarian, breast, colorectal, and melanoma cancer cell lines.^17−19^ Thus, in the present study, the newly synthesized 3-arylisoquinolinones **2**–**8** were evaluated for NQO2 inhibitory activity, as well as determining their antiproliferative activity in a range of cancer cell lines. Finally, the underpinning mechanism of action by which these compounds exert their cytotoxicity was elucidated.

## Results and Discussion

### Chemistry

The synthesis of seven differently substituted 3-arylisoquinolinones **2**–**8** was carried out using the method developed by Khadka and co-workers (Scheme 1).^20^ 4-Fluoro-2-methylbenzoic acid **9** or 5-fluoro-2-methylbenzoic acid **10** was reacted with thionyl chloride and diethylamine to form the amide intermediates **13** and **14**. Then, the amides reacted with the appropriate benzonitriles (**15**–**18**) in the presence of n-BuLi or lithium diisopropylamide (LDA), producing the desired 3-arylisoquinolinones **2**–**8**. The compounds were characterized by ^1^H, ^13^C, and ^19^F NMR spectroscopy (Figures S1–S21).

**Scheme 1:**
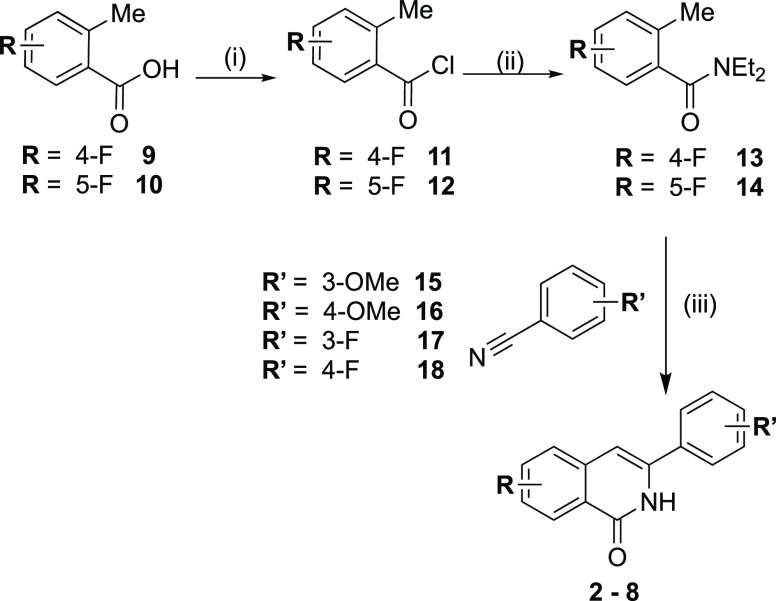
Synthesis of 3-Arylisoquinolinones 2–8 Reagents and conditions: (i) SOCl_2_, reflux, 45 °C; (ii) Et_2_NH, CH_2_Cl_2_, 0 °C; (iii) *n*-BuLi/LDA, dry THF, −78 °C.

### Inhibition of NQO2 Activity

3-Arylisoquinolinones **2**–**8** were examined for their abilities to inhibit the enzyme activity of human recombinant NQO2, with resveratrol as a positive control (IC_50_ 1.0 μM). All of the 3-arylisoquinolinones were inactive against NQO2 at concentrations up to 100 μM.

### Inhibition of Cancer Cell Proliferation

Initially, the human breast cancer cell line MCF-7 was used in a prescreen analysis of the anticancer activity of 3-arylisoquinolinones. The cells were treated with serial concentrations of compounds **2**–**8** for 96 h prior to the assessment of proliferation using the sulforhodamine B (SRB) assay. For each compound, the concentration required to inhibit cell growth by 50% compared to that of the untreated control cells (IC_50_) was determined (Table 1). Compounds with −OCH_3_ or −F substituents on the *meta* position of the aryl ring (**2**, **4**, **6**) showed significant growth inhibition with IC_50_ values of 0.4–0.8 μM, compared to the corresponding compounds with *para* substituents (**3**, **5**, **7**, **8**), which showed IC_50_ values above 50 μM.

**Table 1:**
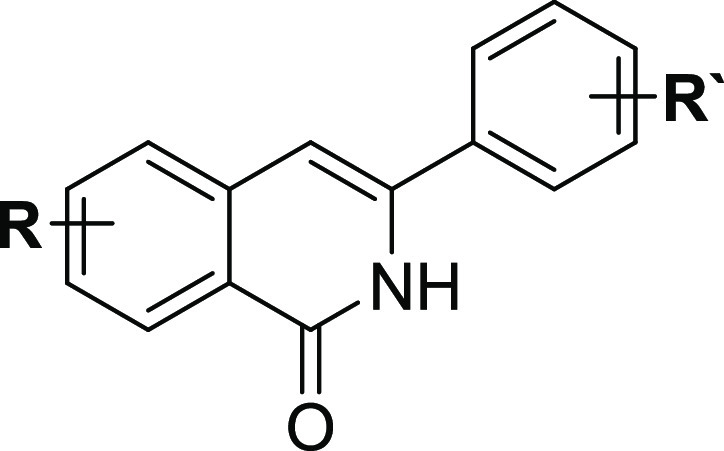
IC_50_ Values (μM, Mean ± SEM) of 3-Arylisoquinolinones Following 96 h Treatment of Several Cancer Cell Lines^a^

cpd.	R	R′	MCF-7 (prescreen)	MDA-MB-231	HepG2	SNU423	A549	HCT116
**2**	6-F	*m*-OMe	0.75 ± 0.13	0.17 ± 0.01	3.12 ± 0.34	0.41 ± 0.03	0.37 ± 0.01	0.22 ± 0.02
**3**	6-F	*p*-OMe	>50	>50	>50	>50	>50	>50
**4**	6-F	*m*-F	0.41 ± 0.09	0.07 ± 0.002	1.44 ± 0.21	0.25 ± 0.03	0.27 ± 0.06	0.13 ± 0.03
**5**	6-F	*p*-F	>50	>50	>50	>50	>50	>50
**6**	7-F	*m*-OMe	0.68 ± 0.13	0.21 ± 0.05	3.03 ± 0.61	0.60 ± 0.06	0.38 ± 0.02	0.19 ± 0.01
**7**	7-F	*p-*OMe	>50					
**8**	7-F	*p*-F	>50					

a IC50 values were derived from dose–response curves of at least three independent experiments using the SRB assay.

In light of this substantial change in activity between the *meta* and *para* compounds, a more comprehensive analysis of the antiproliferative activity was performed for the *meta*-/*para*-3-arylisoquinolinone pairs **2** and **3**, **4**, and **5**, as well as **6**. A panel of breast (MDA-MB-231), liver (HepG2, SNU423), lung (A549), and colon (HCT116) cancer cell lines were treated with the five 3-arylisoquinolinone analogs for 96 h followed by the SRB assay. The dose–response curves (including those for MCF-7) are presented in Figure S22, and their relevant IC_50_ values are listed in Table 1. Interestingly, the results from the five cell lines confirm the prescreen and show the *meta*-substituted compounds to have greater growth-inhibitory activity than their *para-*substituted analogues, with a difference up to 700-fold in IC_50_ values. The differential activity between *meta-* and *para*-3-arylisoquinolinones was reproduced in the National Cancer Institute (NCI) one-dose screen using a panel of 59 cancer cell lines (Figure S23). This screen showed that the *meta* compound **4** was far more potent in the inhibition of growth of many of the cell lines, compared to the less active *para* analog **5** or **7**.

There was no significant difference between the IC_50_ values of **2** (6-F, *m*-OMe) and **6** (7-F, *m*-OMe) across the cell lines, suggesting that the position of fluorine on the primary isoquinolinone ring is not a limiting factor for determining the antiproliferative activity of the *meta* compounds. In comparison to compounds **2** and **6**, the growth-inhibitory activity of **4** was significantly greater in HepG2 (*p* = 0.04) and SNU423 (*p* = 0.0007) cells. Additionally, the IC_50_ value of **4** was markedly lower than that of **2** in HCT116 and **6** in MDA-MB-231 cells (*p* = 0.02).

**Figure 2:**
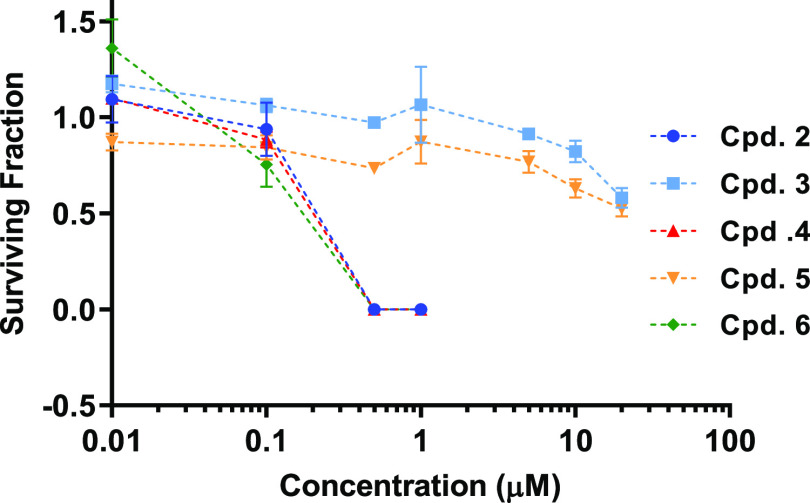
Clonogenic survival of HepG2 cells following prolonged treatment with 3-arylisoquinolinones. HepG2 cells (1000/well) in six-well plates were treated with DMSO or different concentrations of compounds **2–6** for 14 days. Colonies were then fixed, stained, counted, and used for calculating surviving fractions. Graph points on survival curves represent mean ± SEM from three independent experiments.

The clonogenic assay was then employed to confirm the differential activity between the *meta* and *para* 3-arylisoquinolinones and to assess whether the *meta* compounds possessed a cytotoxic effect. HepG2 cells were treated with varying concentrations of **2–6** added once and kept for 14 days before counting colonies and calculating surviving fraction (Figure 2). No colonies were apparent when cells were treated with either 0.5 or 1 μM of **2, 4**, or **6**. In contrast, for **3** or **5**, at concentrations as high as 20 μM, the surviving fraction was only reduced to 0.5. This clearly demonstrates that the *meta-*substituted compounds are far more cytotoxic than their *para* analogues, in agreement with the SRB findings.

In the preceding experiments, the cells were continuously exposed to the compounds prior to scoring for the antiproliferative/cytotoxic effects. To determine whether the potency of the *meta* analogues could be maintained following short drug exposures, the six cancer cell lines were treated with **2**, **4**, and **6** for 24 h followed by a 72 h compound-free incubation period, and determined the antiproliferative effect using the SRB assay. The *meta* compounds were also examined for the presence of differential antiproliferative activity against cancer cells over the normal liver cell line THLE-3. As shown in Table 2, the IC_50_ values of the *meta* compounds after 24 h^+drug^/72^–drug^ treatment were in the low micromolar range and comparable to those following continuous 96 h treatment (Table 1). Moreover, the sensitivity of the six cancer cell lines toward compounds **2**, **4**, and **6** was markedly greater than that of THLE-3 cells. These results suggest that the *meta* compounds could selectively target tumor cells while being sparing to healthy, noncancerous cells.

**Table 2:** IC_50_ Values of *meta*-Substituted 3-Arylisoquinolinones (**2, 4, 6**) Following 24 h^+drug^/72 h^–drug^ Treatment of Six Cancer Cell Lines and One Normal Cell Line (THLE-3)^a^

	mean IC_50_ ± SEM (μM)		
cell lines	cpd. 2	cpd. 4	cpd. 6
MCF-7	1.43 ± 0.30	0.50 ± 0.01	1.60 ± 0.40
MDA-MB-231	0.19 ± 0.04	0.09 ± 0.01	0.22 ± 0.02
HepG2	2.33 ± 0.22	0.86 ± 0.06	1.40 ± 0.06
SNU423	0.58 ± 0.04	0.36 ± 0.01	0.68 ± 0.09
A549	0.83 ± 0.17	0.44 ± 0.08	0.47 ± 0.03
HCT116	0.20 ± 0.01	0.13 ± 0.01	0.21 ± 0.01
THLE-3	>25	>25	>25

a IC50 values were obtained from dose–response curves of three independent experiments using SRB assay.

### Identification of the Potential Cellular Target with COMPARE Analysis

The greatest growth-inhibitory activity has been shown here to be elicited by the *meta*-substituted 3-arylisoquinolinone **4** with submicromolar IC_50_ values against several cancer cell types, the lowest being 70 nM. To gain insight into the potential molecular mechanism corresponding to the cytotoxic effect of **4**, an NCI COMPARE analysis was carried out, where the NCI five-dose cell growth data of **4** (see Figure S24) were compared against NCI databases (Standard and Synthetic). COMPARE algorithm uses Pearson correlation coefficients to correlate and rank the compared compounds from the NCI databases to the seed compound **4** (NCS number 795055).^21^ Compare solutions (CS) with a correlation coefficient greater than 0.8 indicate a strong correlation, suggesting that the seed compound may have a mechanism of action similar to that of the highly correlated compounds.^22,23^

**Figure 3:**
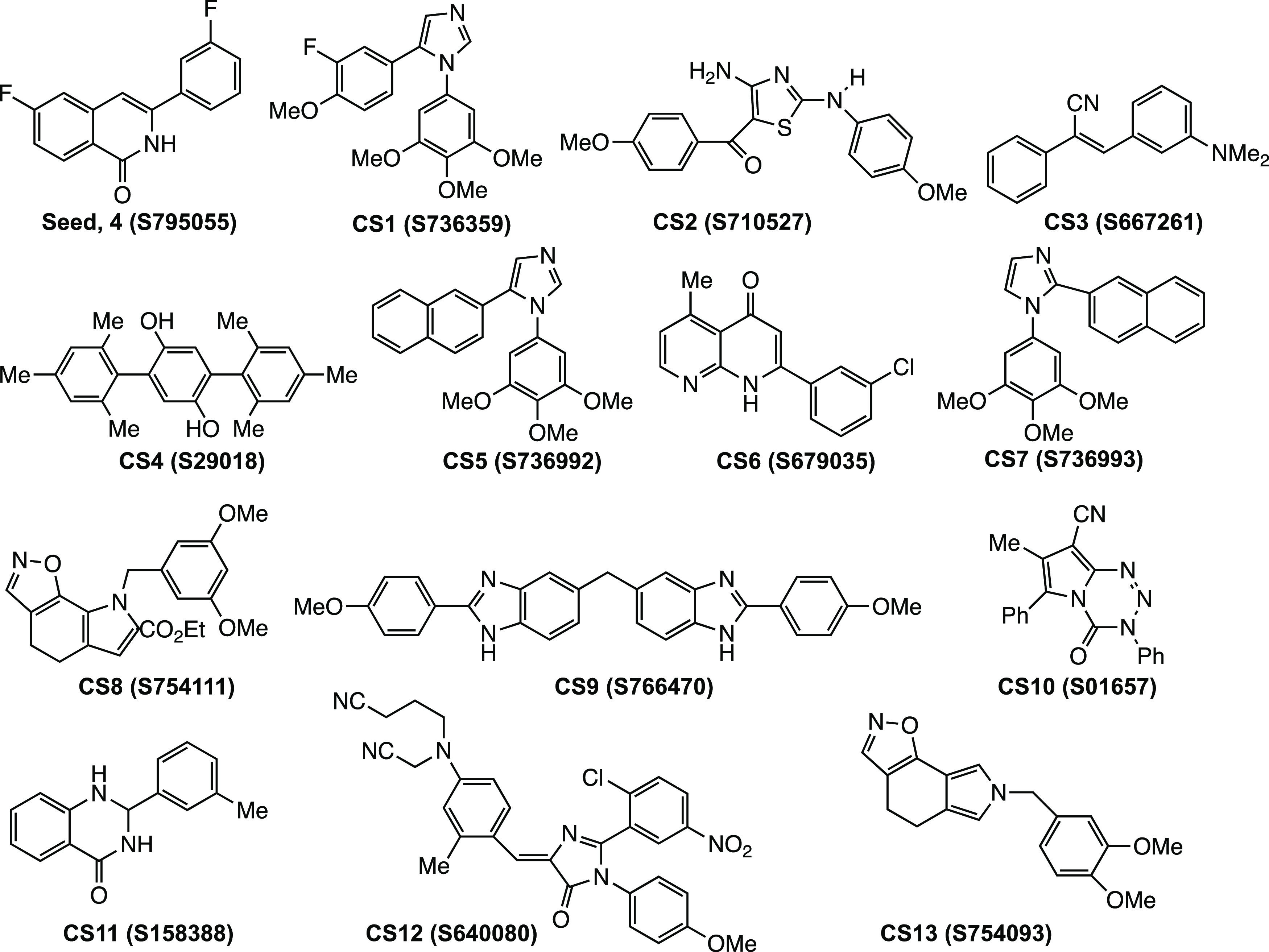
Chemical structures of the top 13 compounds from COMPARE analysis of seed compound **4** (S795055) against the Synthetic NCI Database.

Compound **4** was compared with the NCI Standard Database containing 171 drugs in clinical use with known mechanisms of action. The solutions for the top 5 compounds are given in Table S1. In this analysis, the correlation coefficient for the top-ranked solution was only 0.583, which is far below the minimum acceptable value to predict the mechanism of action. When COMPARE was made with the NCI Synthetic Database (∼40,000 compounds with known and unknown mechanisms of action), the solutions for the top 13 compounds gave correlation coefficients ranging from 0.772 to 0.821 (Table 3). The chemical structures of these 13 compounds along with **4** are given in Figure 3. Seven of these compounds were identified to bind to tubulin, hence acting as microtubule-targeting agents (MTAs). Further, the top match is 1,5-diaryl-1*H*-imidazole (**CS1, S736359**), which interestingly has *meta*-F, and the fifth match is another imidazole derivative (**CS5, S736992**) but without a −F substituent. Both matches have been reported to bind to the colchicine site of tubulin and possess vascular disrupting activity.^24,25^ Notably, the results of COMPARE analysis of the NCI Standard Database revealed that two of the top five compounds are also MTAs. Consequently, **4** was predicted to target microtubules, and the molecular modeling and experiments outlined below were designed to examine this prediction.

**Table 3:** COMPARE Analysis Results for Compound **4** (S795055, Five-Dose Data) against the Synthetic NCI Database (Top 13 Results)

compare solution	NSC number	mechanism of action	correlation coefficient	references
**CS1**	S736359	binding to the colchicine site of tubulin and vascular disruption	0.821	(24, 25)
**CS2**	S710527	transcription factor NF-kB	0.810	patent: WO2007118149A2
**CS3**	S667261	inhibition of peroxisome proliferator-activated receptors (PPAR)	0.800	patent: WO2013072390A2
**CS4**	S29018	unknown	0.798	
**CS5**	S736992	binding to the colchicine site of tubulin and vascular disruption	0.796	(24, 25)
**CS6**	S679035	inhibition of tubulin polymerization	0.782	(26)
**CS7**	S736993	binding to the colchicine site of tubulin	0.778	(24)
**CS8**	S754111	inhibition of tumor proliferation without a defined mechanism	0.778	(27)
**CS9**	S766470	unknown	0.777	
**CS10**	S701657	inhibition of tubulin polymerization	0.775	(28)
**CS11**	S158388	inhibition of tubulin polymerization	0.775	(29)
**CS12**	S640080	unknown	0.774	
**CS13**	S754093	inhibition of tubulin polymerization	0.772	(30)

### Molecular Modeling

**Figure 4:**
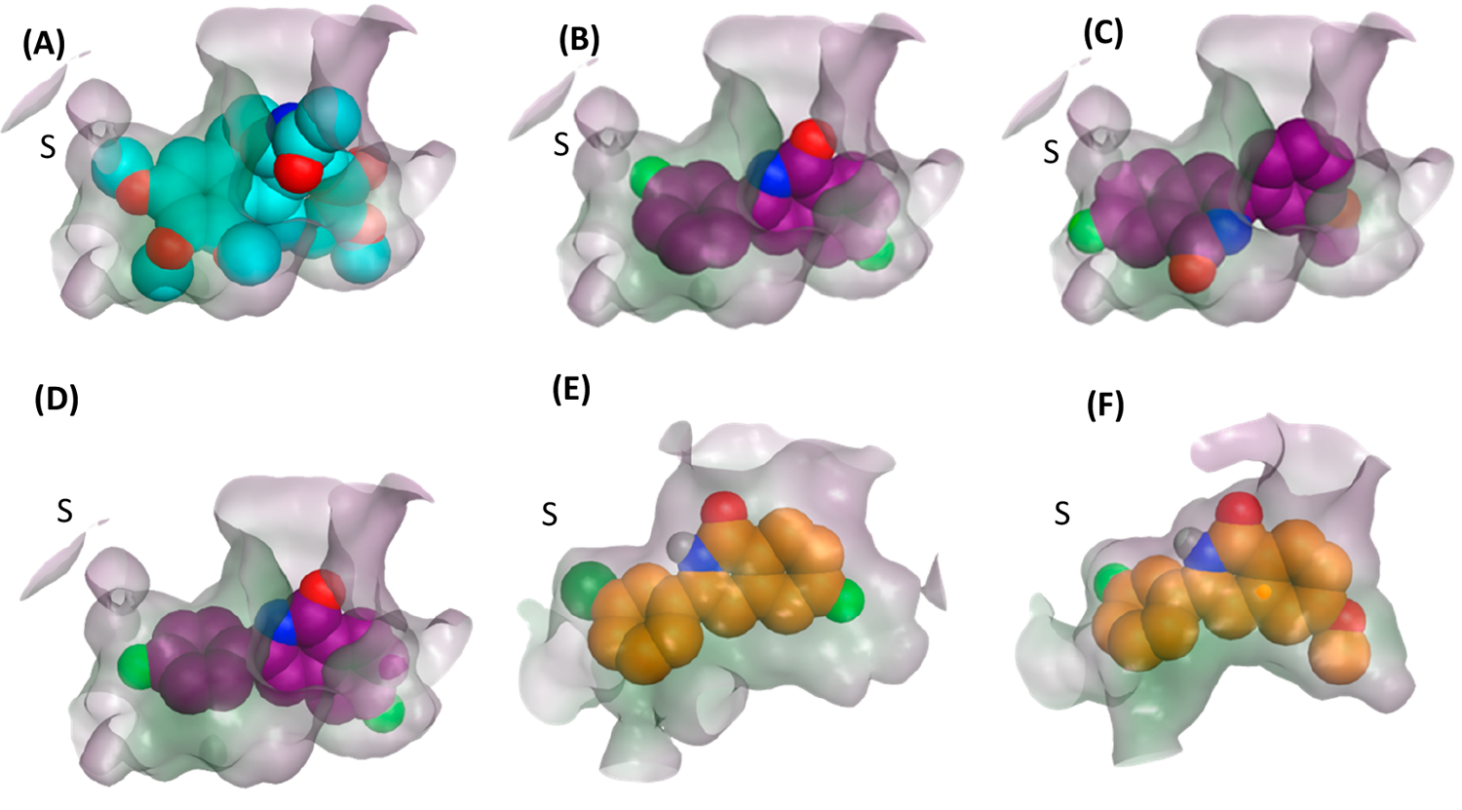
(A) Crystallographic pose of colchicine (cyan) in tubulin binding pocket, shown as a surface, indicating polar (purple) and nonpolar (green) regions; docked pose (dark purple) of (B) **4**, (C) **6** in its flipped orientation, and (D) **5**. MD-refined pose (orange) of (E) compound **md1** and (F) compound **md4**, based on simulation of **4**. Subpocket is marked S.

The ability of compounds **2**–**8** to interact with the colchicine-binding site of tubulin was assessed using computational docking using the FRED docking software and the Chemgauss4 scoring function (OpenEye Scientific Software, Inc.). The top-ranked poses of all compounds docked with the substituted phenyl group occupying the region of the active site where the 1,2,3-trimethoxyphenyl ring of colchicine locates crystallographically (Figure 4A,B). The exception was *meta*-OMe 3-aryl compound **6**, which adopted a flipped orientation in its top-ranked pose, where the isoquinolinone ring occupies this region; this pose has a Chemgauss4 score of −11.1 (Figure 4C), with a lower-ranked pose similar to that of the other compounds scoring −9.9.

For the commonly favored pose of **2**–**8**, the substituted phenyl ring of the ligand forms CH−π interactions with the Leu248 and Leu255 residues of the active site. For *meta*-substituted compounds such as **4**, the *meta* substituent was predicted to occupy a subpocket region formed by residues Leu242, Leu252, and Leu255 (marked S in Figure 4B) and filled by the methoxy group of colchicine in the X-ray structure (Figure 4A). However, the *para* orientation was unable to fill this subpocket (Figure 4D). Accordingly, the docking scores were 0.5–1.0 units more favorable for **2**, **4**, and **6** compared to their respective *para*-substituted compounds **3**, **5**, and **7** (Table S2). We note that in all cases, the magnitude of the docking scores were less than that of colchicine (−15.2), which may to some extent be expected given its considerably larger size.

**Figure 5:**
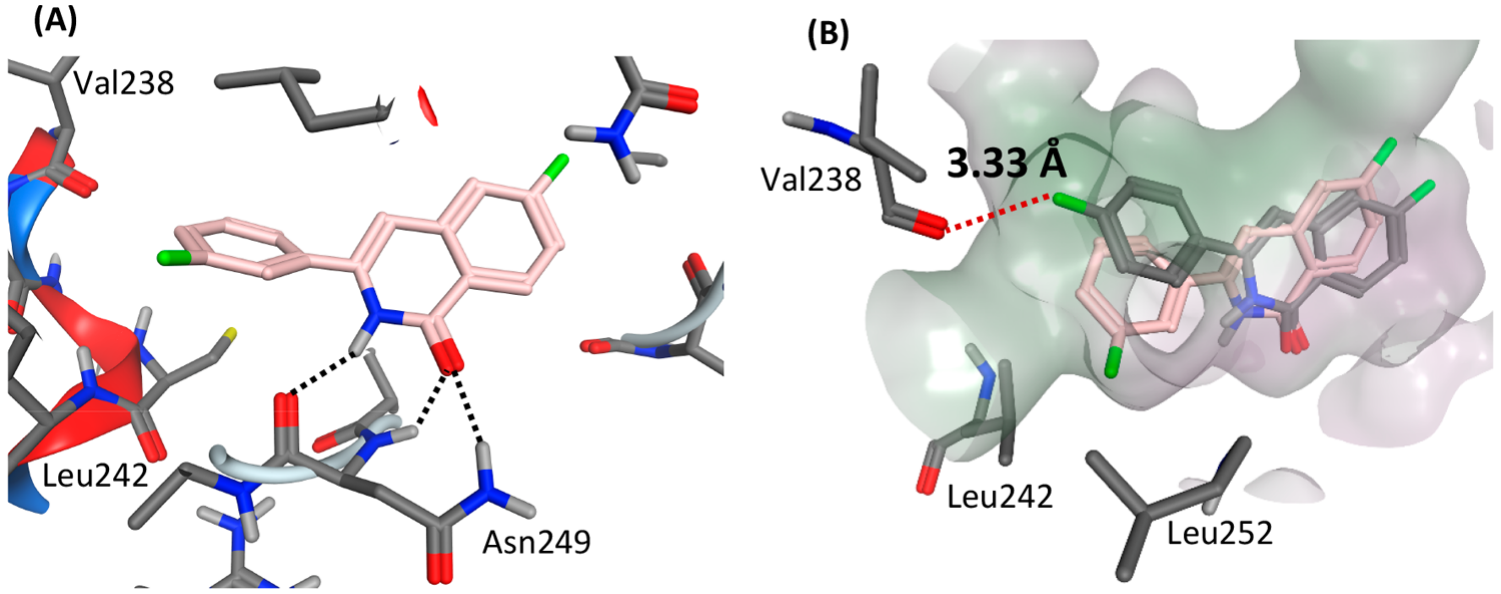
(A) MD-refined pose of compound **4** (pink); hydrogen bonds are indicated by black dotted lines. (B) Superposition of compounds **4** (pink) and **5** (gray); F···O close contact indicated by red dotted line and value of MD-averaged distance.

To refine the predicted binding mode of compound **4**, a 50 ns molecular dynamics simulation of its docked pose in tubulin in explicit aqueous solvent was performed. The ligand remained in its overall docked orientation in the binding pocket, with the occupation of the subpocket by the *meta*-F group (Table S4); however, during the MD simulation, the isoquinolinone amide H and O atoms of **4** also established several hydrogen bonds with the backbone and side chain of Asn249 (Figure 5A, Table 4). The two hydrogen bonds to the Asn249 backbone persisted over the simulation but were more sporadically formed with the Asn249 side chain.

**Table 4:** Average Interatomic Distances (in Å) between Tubulin Residue Asn249 and Amide O_am_ and N_am_ Atoms of Ligand from Final 10 ns of MD Simulation of Ligand–Tubulin Complexes (Standard Deviations in Parentheses)

distance (Å)			
compound	Asn249 N···O_am_	Asn249 O···N_am_	Asn249 N_δ2_···O_am_
**2**	3.23 (0.31)	3.04 (0.23)	4.56 (0.87)
**3**	3.06 (0.27)	3.24 (0.35)	4.77 (0.59)
**4**	3.03 (0.19)	3.05 (0.22)	3.41 (0.92)
**5**	3.09 (0.26)	3.02 (0.22)	4.64 (0.85)
**6**	3.05 (0.23)	3.12 (0.29)	5.05 (0.83)
**7**	3.68 (1.17)	3.43 (1.02)	5.15 (1.26)
**8**	3.17 (0.34)	3.01 (0.21)	4.87 (0.62)
**md1**	3.19 (0.30)	3.09 (0.24)	4.09 (1.17)
**md2**	3.34 (0.42)	2.88 (0.14)	5.07 (0.63)
**md3**	2.95 (0.17)	3.07 (0.21)	4.46 (0.37)
**md4**	3.07 (0.22)	3.08 (0.21)	2.95 (0.19)
**md5**	2.98 (0.19)	3.03 (0.18)	4.92 (0.83)
**md6**	3.11 (0.26)	3.11 (0.26)	4.94 (0.65)

The binding free energy of ligand **4** was then computed, applying the MM/GBSA method^55^ to compute the average affinity over the last 10 ns of the trajectory. A calculated Δ*G*_tot_ of −39.3 kcal/mol was predicted (Table 5), composed of a significant nonelectrostatic component Δ*G*_nonel_ of −42.2 kcal/mol, indicating good shape complementarity in the protein pocket, and largely canceling electrostatic contributions to binding Δ*G*_el_, from interaction with protein and solvent (Table 5). The predicted binding affinity for **4** is nevertheless reduced compared to that observed for the rather larger colchicine molecule, with Δ*G*_tot_ and Δ*G*_nonel_ values of −45.2 and −66.3 kcal/mol, respectively (Table 5).

**Table 5:** Calculated Total Binding Free Energies, Δ*G*_tot_, and Electrostatic (Δ*G*_el_) and Nonelectrostatic (Δ*G*_nonel_) Contributions, Using the MM/GBSA Method (Energies in kcal/mol; Standard deviations in Parentheses)

compound	R	R′	Δ*G*_tot_	Δ*G*_el_	Δ*G*_nonel_
**colchicine**			–45.2 (4.5)	21.3 (7.2)	–66.3 (3.3)
**2**	6-F	m-OMe	–37.4 (2.7)	8.7 (3.9)	–46.1 (2.1)
**3**	6-F	p-OMe	–34.4 (3.0)	12.1 (3.9)	–46.4 (2.3)
**4**	6-F	m-F	–39.3 (2.8)	3.0 (4.3)	–42.2 (2.5)
**5**	6-F	p-F	–36.4 (3.2)	6.9 (4.8)	–43.2 (2.2)
**6**	7-F	m-OMe	–37.1 (2.7)	9.4 (4.6)	–46.5 (1.8)
**7**	7-F	p-OMe	–35.9 (3.4)	11.5 (6.1)	–47.3 (2.3)
**8**	7-F	p-F	–32.7 (2.9)	9.4 (4.6)	–42.2 (1.9)
**md1**	6-F	m-Cl	–41.2 (3.2)	4.6 (4.8)	–45.8 (2.1)
**md2**	6-F	m-NHMe	–43.6 (3.0)	4.0 (4.0)	–47.5 (2.2)
**md3**	7-F	m-F	–36.1 (3.0)	8.5 (3.1)	–44.5 (2.0)
**md4**	6-OMe	m-F	–46.7 (2.8)	2.1 (3.7)	–48.8 (2.4)
**md5**	6,7-OMe	m-F	–44.9 (3.8)	9.4 (4.4)	–54.3 (2.2)
**md6**	6-OMe	m-NHMe	–45.4 (4.1)	5.4 (5.4)	–50.8 (2.0)

Furthermore, the ability of the other *meta-*substituted compounds, **2** and **6**, to assume this hydrogen-bonded pose of **4** was evaluated. Thus, MD simulations of **2** and **6** were started from the 30 ns orientation of **4**, equilibrating for 20 ns and computing the MM/GBSA affinity over a further 10 ns. Both compounds were able to retain this pose over the trajectory (Tables 4 and S4). Interestingly, **2** and **6** had a lower affinity than **4** and similar to each other, with Δ*G*_tot_ values of −37.4 and −37.1 kcal/mol, respectively (Table 5); this reflected the experimentally measured relative activity of **2**, **4**, and **6** (Table 1). From these simulations, both 6-F and 7-F derivatives appear to be tolerated within the tubulin site (Table S4).

Using the same protocol, the ability of the *para*-substituted compounds **3**, **5**, **7**, and **8** was then assessed to maintain the MD pose adopted by **4***via* simulation. The computed affinities range from −32.7 to −36.4 kcal/mol (Table 5), less than their *meta-*substituted counterparts by 1.2–3.0 kcal/mol. These compounds appear able to maintain the hydrogen bonding to tubulin via their isoquinolinone amide group (Table 4), but the *para* substituent experienced a close contact with the backbone carbonyl oxygen of Val238. For example, an unfavorable close contact between the Val238 backbone O and the *para*-F atom of compound **5** was observed, with an MD-averaged distance of 3.33 ± 0.41 Å (Figure 5B, Table S3); for compound **4**, however, this distance was larger, at 5.25 ± 0.47 Å.

Finally, to computationally probe the structure–activity relationship of **4** further, we use the above MD-based protocol to calculate Δ*G*_tot_ values for derivatives **md1**–**md6** initiated from the MD pose of **4** (Table 5). Substitution of the *meta*-F to *meta*-Cl group in **md1** resulted in an increased affinity by 1.9 kcal/mol, with greater filling of the S subpocket (Figure 4E, Table 5). When in **md2** a methylamine group instead replaces the 3-F, there is a more favorable Δ*G*_tot_ by 4.3 kcal/mol; this gain appears to arise from an additional hydrogen-bond interaction, formed by the methylamine NH of **md2** with the backbone O of Val238, having an MD average N···O distance of 2.92 ± 0.34 Å. To forge this interaction, the 3-aryl group is rotated in the binding pocket away from subpocket S (Table S4).

Putative compounds **md3**–**md5** consider substitutions at the 6, 7, and both these positions in compound **4**: changing the 6-F in **4** to 7-F to **md3** results in a loss of binding free energy by 3.2 kcal/mol (Table 5). However, mutation from 6-F to 6-OMe in **md4** leads to a significant gain in Δ*G*_tot_, to a value of −46.7 kcal/mol; this Δ*G*_tot_ value is improved over not only **4** but also colchicine, which has a computed free binding energy of −45.2 kcal/mol (Table 5). Indeed, the ligand 3-F and 6-OMe groups fits well into the tubulin site (Figure 4F); hydrogen bonding is maintained well by **md4**, particularly with the side chain of Asn249 (Table 4). Alteration of compound **4** to have an OMe group at both 6 and 7 positions (**md5**) is not well accommodated; this may be due to a mismatch of the nonpolar 7-OMe group with the polar backbone of Val238 (Figure 4F). Indeed, this position is occupied by the oxo group of colchicine in its crystallographic pose (Figure 4A). Finally, we also note that combining a 3-methylamine and 6-OMe substituent in compound **md6** does not appear to be additively favorable, with a computed Δ*G*_tot_ of −45.4 kcal/mol (Table 5). Nevertheless, computed affinities of compounds **md1**–**md6** suggest directions for further improvement of lead compound **4**.

### Effect on Tubulin Polymerization

**Figure 6:**
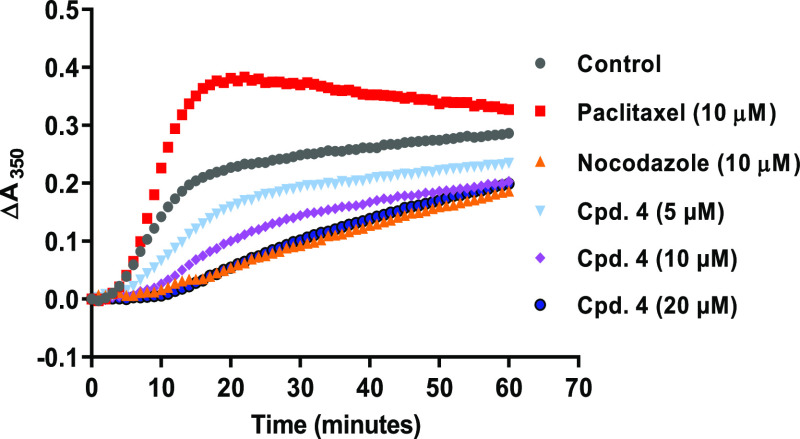
3-Arylisoquinolinone 4 inhibits tubulin polymerization. Tubulin was incubated at 37 °C in the presence of DMSO, paclitaxel, nocodazole, or 4. The absorbance was measured at 350 nm every minute for 1 h using a microplate reader. The delta absorbance (Δ*A*_350_) was calculated by subtracting the absorbance value of zero time from those of the subsequent time points, and then plotted versus time for each condition.

Experimentally, the direct interaction of **4** with tubulin was investigated in a cell-free system using a tubulin polymerization assay. The polymerization of purified tubulin into turbid microtubules was measured by a change in the absorbance at 37 °C in the presence of DMSO (negative control), paclitaxel and nocodazole (10 μM, positive polymerizing and depolymerizing controls), or different concentrations of **4** (Figure 6). In control samples, α- and β-tubulin subunits were able to heterodimerize and assemble into microtubules in a time-dependent manner as indicated by increasing absorbance values over time. Paclitaxel generated higher absorbance values briefly after the onset of the polymerization process, compared to the DMSO control. This indicates the incidence of fast polymerization associated with the formation of a denser microtubule mass. In contrast, **4** hindered tubulin polymerization in a concentration-dependent manner, subsequently reduced the final polymer mass of microtubules, and at its highest concentration tested (20 μM) **4** was as effective in suppressing tubulin polymerization as the positive control (nocodazole). It is worth mentioning that microtubule dynamics can be potently altered by microtubule-stabilizing or -destabilizing compounds at doses 10- to 100-fold lower than those required for increasing or decreasing the microtubule polymer mass.^31^ In fact, the anticancer efficacy of MTAs, such as paclitaxel and vinca alkaloids, has relied on disrupting the microtubule dynamics rather than changing the polymer mass, and therefore they are clinically dosed at very low concentrations.^31^

The impact of **4** on microtubule dynamics was further determined by assessing cellular downstream effects, including mitotic arrest, induction of apoptosis, and/or interference with tumor vasculature, all of which are well known in the classic MTAs.

### Effects on Cell Cycle and Apoptosis

**Figure 7:**
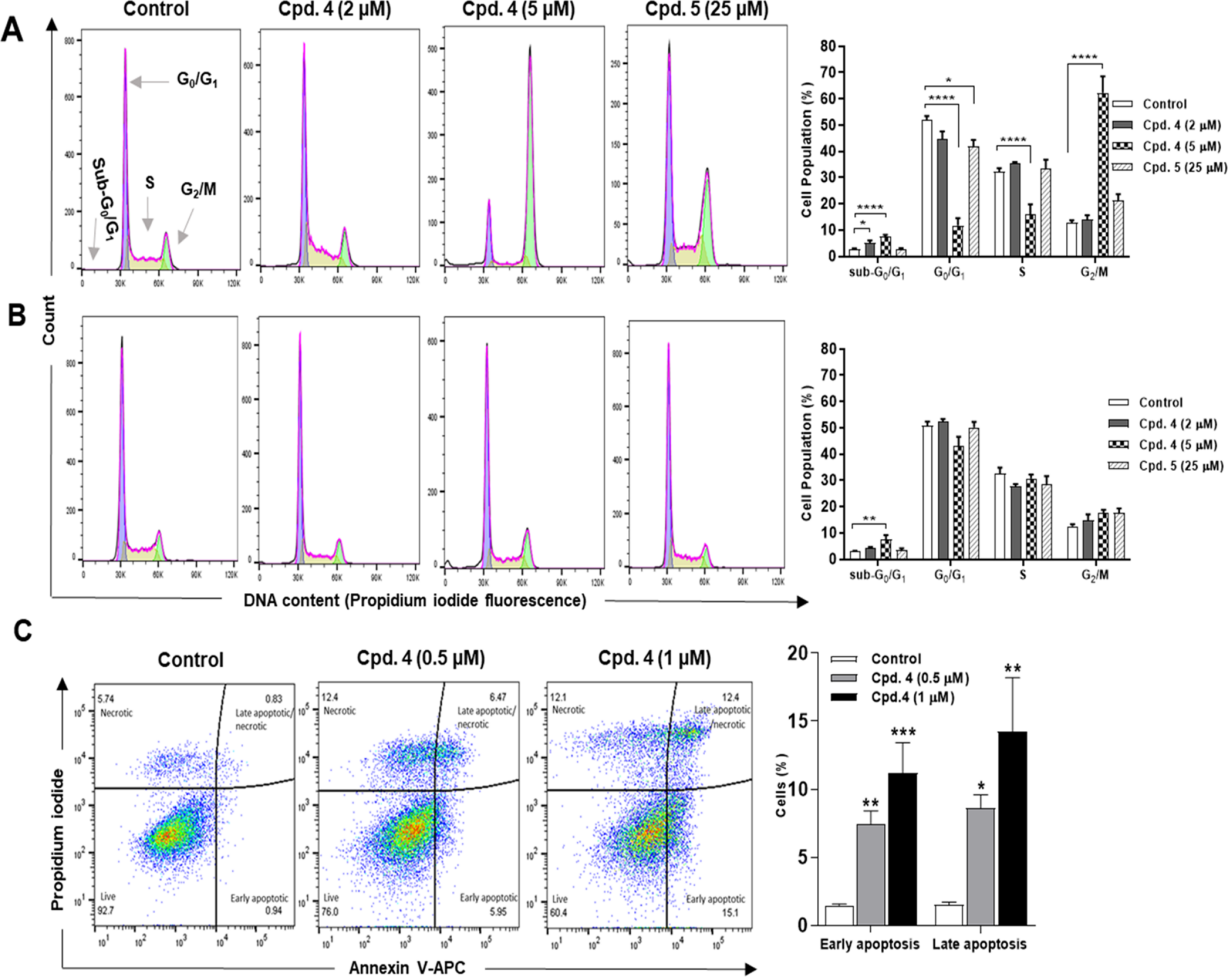
3-Arylisoquinolinone **4** induces reversible cell cycle arrest at the *G*_2_/*M* phase and apoptosis. (A) HepG2 cells were analyzed for cell cycle progression using flow cytometry following 24 h treatment with DMSO (control), **4** or 5. (B) HepG2 cells were treated for 24 h and the compound-containing medium was replaced with a fresh one for a further 24 h followed by cell cycle determination. (C) Induction of apoptosis in SNU423 cells was measured by Annexin V/PI assay following 24 h treatment. The cell cycle histograms and the quadrant dot plots are representatives of at least three independent experiments. Data are presented as mean ± SEM. *p ≤ 0.05, **p ≤ 0.01, ***p ≤ 0.001, and ****p ≤ 0.0001 compared to the control according to one-way ANOVA followed by Dunnett’s multiple comparisons test.

Given that microtubules play an important role in the progression of cells through the cell cycle, MTAs have been reported to be associated with mitotic arrest and eventually apoptosis.^31^ Therefore, the effects of the *meta* compound **4** and the corresponding *para* analogue **5** on the cell cycle of HepG2 cells were examined after 24 h treatment using flow cytometric analysis of the DNA content. As shown in Figure 7A, the untreated HepG2 cells had a typical cell cycle profile, whereby the largest and smallest proportions of cells were, respectively, located in *G*_0_/*G*_1_ (51.8 ± 1.7%) and *G*_2_/*M* (12.8 ± 1.0%) phases. Exposure of HepG2 cells to **4** at 5 μM caused a significant accumulation of cells in the *G*_2_/*M* (62.2 ± 6.3%) phase with a concomitant reduction in cell percentage at *G*_0_/*G*_1_ (11.7 ± 2.9%) and *S* (12.4 ± 2.2%) phases compared to the control (*p* ≤ 0.0001). Such mitotic block is strongly attributed to the disruption of microtubule function. On the other hand, at a concentration of 25 μM, **4** only decreased cell proportion at the *G*_0_/*G*_1_ phase, compared to the control (*p* = 0.032). The cell cycle analysis can normally detect small DNA fragments that appear at lower values of the DNA content histograms in a region called sub-*G*_0_/*G*_1_ or sub-*G*_1_. These fragments can be attributed to the presence of apoptotic (sub-diploid) cells.^32^ Compound **4**-treated HepG2 cells (Figure 7A) showed a significant sub-*G*_0_/*G*_1_ cell population, compared to the control (2 μM, *p* = 0.04; 5 μM, *p* < 0.0001). The induction of apoptosis by **4** was further confirmed on another cancer cell line, SNU423, through the use of Annexin V/Propidium iodide flow cytometry assay following 24 h treatment with concentrations of 0.5 and 1 μM (Figure 7C). Compared to the control, SNU423 cells treated with **4** demonstrated a significant increase in the percentages of early apoptotic (0.5 μM, *p* = 0.009; 1 μM, *p* = 0.0008) and late apoptotic cells (0.5 μM, *p* = 0.04; 1 μM, *p* = 0.003). These data clearly indicate that the antimitotic effect of **4** was associated with induction of apoptosis.

Further, to gain insight into the sustainability of alterations induced by 3-arylisoquinolinones on cell cycle, the compound-containing medium was removed after 24 h treatment and replaced with fresh medium for an additional 24 h (i.e., 24 h^+drug^/24 h^–drug^), prior to cell cycle analysis (Figure 7B). The percentages of HepG2 cells at *G*_0_/*G*_1_, *S*, and *G*_2_/*M* phases were restored to values similar to those of the control, while a high cell population at sub-*G*_0_/*G*_1_ was still displayed (*p* = 0.0096). Moreover, the cell population at the *G*_2_/*M* phase following exposure to **4** (5 μM) for 24 h^+drug^/24 h^–drug^ was significantly lower (17.49 ± 1.37%) compared to that of 24 h treatment (62.2 ± 6.3%, *p* ≤ 0.0001), indicating reversibility of the mitotic arrest. It has been reported that the reversion of MTA-induced mitotic blockade is a compound-specific property and even slight structural modifications in compounds within a particular class are associated with significant differences in their abilities to maintain mitotic arrest after compound washout.^33^ For instance, paclitaxel was a moderately reversible mitotic blocker; vinblastine, colcemid, and nocodazole were highly reversible; and vincristine and colchicine were irreversible.^33^ Generally, compounds with reversible therapeutic efficacy are favored since their effects, including toxicities, are possibly eliminated upon drug withdrawal, and this is an important criterion for use in patients.^34^

### Effects on Endothelial Tube Formation and Disruption

In addition to their antimitotic effects on cancer cells, some MTAs can alter the function of endothelial cells lining tumor vasculature resulting in inhibition of angiogenesis and/or disruption of the preestablished tumor vessels (collectively known as antivascular actions).^35^ Since the *meta*-F-substituted isoquinolinone **4** has demonstrated targeting of microtubules, its antivascular activities were further explored. Some MTAs have been reported to exhibit antivascular effects at low concentrations that do not induce cytotoxicity to the endothelial cells;^25,35,36^ therefore, the weakly tumor cytotoxic **5** (*para*-F-substituted isoquinolinone) was also tested. The work was carried out *in vitro* using the primary human umbilical vascular endothelial cells (HUVECs), which can assemble into capillary-like tubes when grown on an extracellular matrix substrate such as Matrigel.^37^

**Figure 8:**
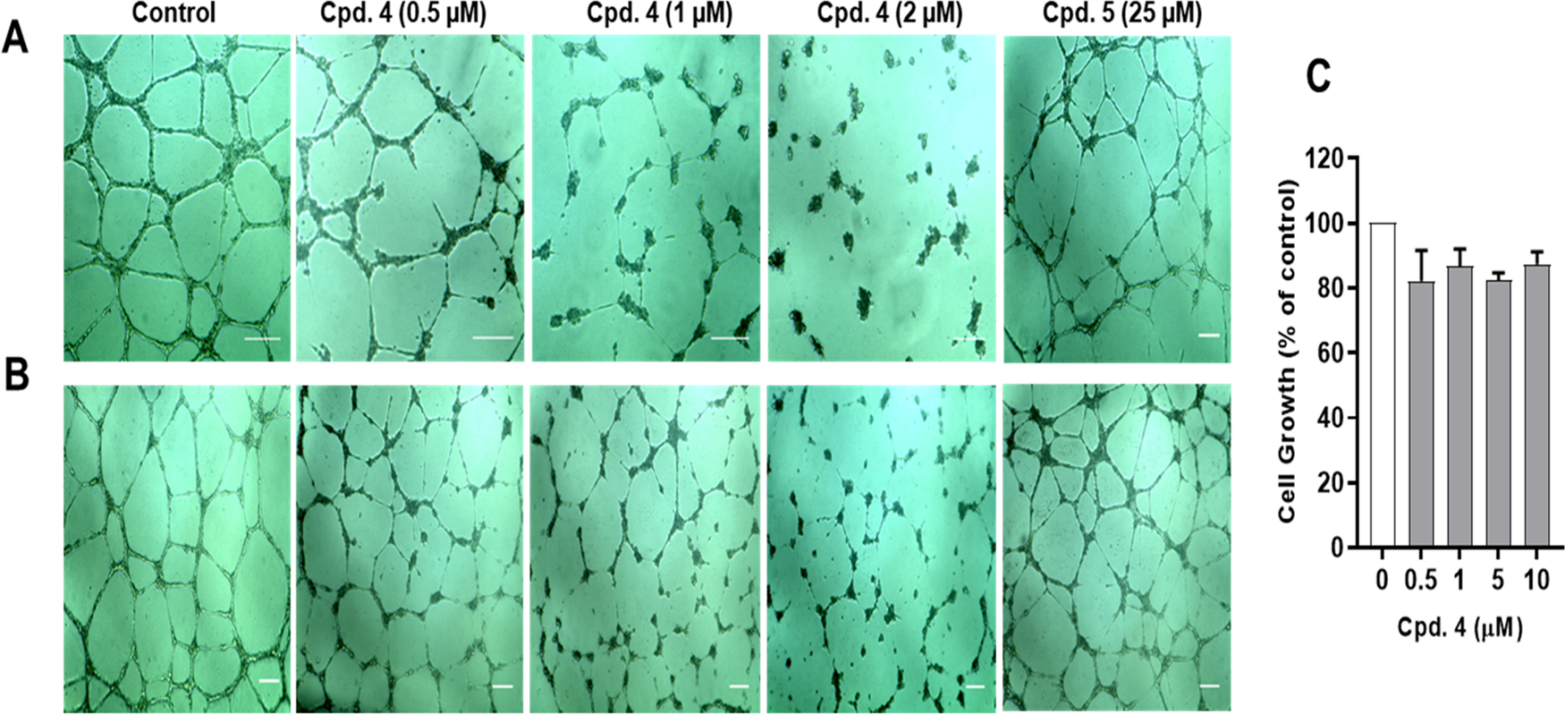
Compound **4** inhibits tube formation and disrupts preestablished tubes of endothelial cells at noncytotoxic concentrations. (A) HUVECs were seeded on Matrigel-coated wells with a medium containing DMSO (control) or the indicated concentrations of 4 or 5 for 16 h. (B) After allowing HUVECs to form capillary-like tubes on Matrigel-coated wells for 4 h, the cells were treated with DMSO, **4**, or **5** for an additional 16 h. The morphology of cells was observed under a light microscope following the 16 h incubation period (A, B) and the bright-field images were captured at 4× magnification. The presented images are representatives of three independent experiments. Scale bar = 200 μm. (C) Effect of **4** on endothelial cells proliferation. HUVECs were treated with a range of concentrations of **4** for 24 h followed by SRB assay. Each column represents the mean ± SEM of three independent experiments.

Figure 8A shows that HUVECs in the control group were able to form a network of interconnecting, capillary-like tubes within 16 h following seeding. Compound **4** at 2 μM markedly suppressed tube formation in a concentration-dependent manner, and cells remained disconnected. In contrast, **5** did not significantly affect the process of tube formation at a high concentration (25 μM). Moreover, networks of HUVEC-based tubes were exposed for 16 h to different concentrations of **4** and **5** to assess their potential tube disruptive effects (Figure 8B). In contrast to **5**, **4** was able to disrupt the preestablished capillary-like tubes formed by HUVECs in a concentration-dependent manner, compared to the control. The disruption was more pronounced at 1 and 2 μM of **4**, as evidenced by a decrease in tube junctions and formation of short distorted tubes.

Compound **4** was further elucidated whether it blocked the formation of capillary-like tubes and disrupted the already existing tubes of HUVECs as a result of inducing endothelial cell death. Accordingly, the growth of HUVECs treated with **4** was investigated using the SRB assay. HUVECs were plated on 96-well plates without prior coating with Matrigel and then exposed to different concentrations of **4** for 24 h. As shown in Figure 8C, **4** was not able to significantly diminish the proliferation of HUVECs at concentrations that caused antivascular effects or even at a concentration of 10 μM. Thus, the antivascular effects of **4** were not correlated with induction of cytotoxicity in HUVECs, which is consistent with other MTAs,^25,38−40^ suggesting that **4** could be a potential antiangiogenic and vascular disrupting agent. It is worth mentioning that some MTAs, particularly microtubule-destabilizing agents such as combretastatins and vinca alkaloids, displayed both antiangiogenic and vascular disrupting effects in preclinical models and they are still under clinical investigations for these effects.^35,41,42^

**Figure 9:**
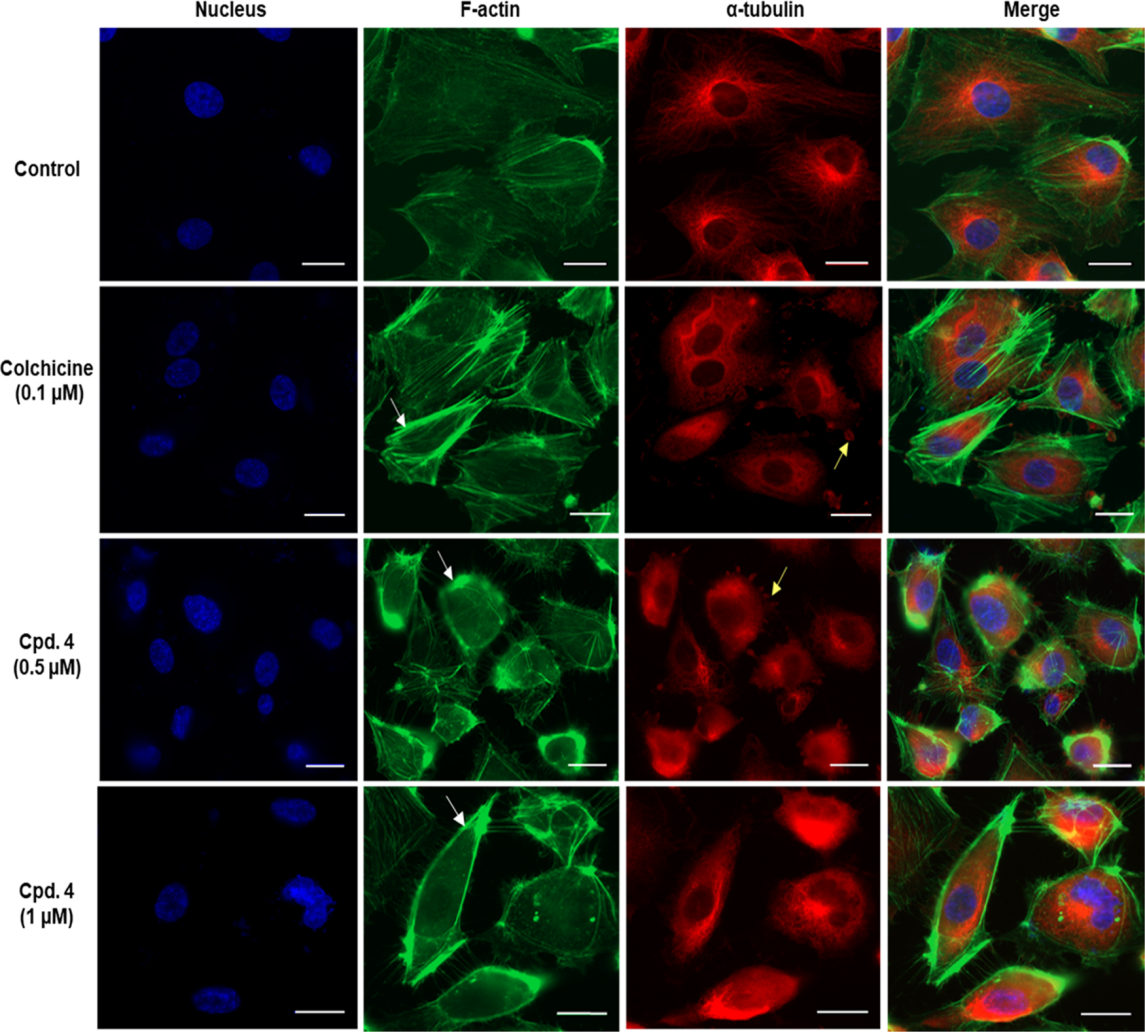
Compound **4** disrupts the cytoskeleton network in endothelial cells. HUVECs were treated with DMSO (control), colchicine (positive control), or 4 for 24 h. Cells were then formaldehyde-fixed and stained for tubulin (anti-α tubulin then CY5 IgG, red), F-actin (phalloidin-Atto 488, green), and nuclei (DAPI, blue). Fluorescent images were acquired at 63× magnification using an inverted microscope. The white arrows indicate actin stress fibers, and the yellow arrows indicate tubulin blebs. The presented images are representatives of three independent experiments. Scale bar = 20 μm.

The vascular disrupting activity demonstrated by MTAs is mainly attributed to structural alterations of the endothelial cytoskeleton, rather than the death of endothelial cells.^43^ Accordingly, the effect of **4** on the cytoskeleton network (actin and microtubules) was explored by means of immunofluorescence. HUVECs were exposed to 24 h treatment with either **4** or colchicine as a positive cytoskeleton-disrupting agent.^44^ The cells were then fixed, stained, and microscopically examined for actin, α-tubulin subunits of the microtubules, and the nuclei. As shown in Figure 9, the actin and microtubules in the control were organized as long filaments around the cell nucleus. Exposure of cells to **4** caused a marked disruption in the cytoskeleton network, similar to colchicine. The disruption was characterized by shortened microtubules with outer blebbing filled with tubulin and thicker and brighter actin fibers (stress fibers). Many rounded and retracted cells were also observed in HUVECs treated with **4**. These alterations were demonstrated previously with combretastatin A4 phosphate and were correlated to microtubule destabilization.^39^ Taken together, **4** induced morphological alterations in endothelial cells are possibly the reason behind the disruption of the preexisting tubes of HUVECs cultured on Matrigel.

## Conclusions

The current study has provided compelling evidence that slight structural modifications on the aryl ring of 3-arylisoquinolinones conferred a remarkable impact on their cytotoxic potential; the *meta* compounds were significantly more effective than the corresponding *para* analogues. The *meta*-substituted 3-arylisoquinolinones exhibited broad-spectrum antiproliferative activity as well as cancer cell selectivity. The findings reveal that microtubules are the biological target of compound **4**. Accordingly, the *meta*-substituted 3-arylisoquinolinones can be classified, for the first time, as MTAs with potential antimitotic, antiangiogenic, and vascular disrupting properties. Indeed, computational docking and MD simulations indicate that compound **4** can interact well with the colchicine-binding pocket of tubulin; from these simulations, **4** is predicted to have a more favorable computed free energy of binding relative to the corresponding *para*-substituted molecule. This appears to arise largely due to the occupation of a tubulin subpocket due to the *meta* orientation of the group; the *para*-substituted compound by contrast is unable to interact with this subpocket and experiences unfavorable steric and electrostatic interactions. We further explore the structure–activity relationship of lead compound **4** by simulating tubulin interactions with putative compounds **md2**–**md8**; these ligands examine other substitutions at the 3, 6, and 7 positions and provide potential future design directions. Overall, these findings should stimulate further investigation of the *in vivo* efficacy of this novel structural class of compounds.

## Experimental Section

### Materials and Instrumentation for the Chemical Synthesis

Chemicals were purchased from Aldrich Chemical Co., Gillingham, U.K. Syntheses were monitored by TLC on precoated 60 F_254_ silica gel aluminum-backed plates (Merck, Darmstadt). Visualization of spots for TLC was performed using a 3% vanillin in 1% H_2_SO_4_/ethanol solution, 1% KMnO_4_ in 7% K_2_CO_3_/10% NaOH solution, and a UV GL-58 Mineral-Light lamp. Flash column grade 40–63 μm silica gel (Apollo scientific, Stockport, U.K.) was used in preparative scale column chromatography. NMR spectra were recorded using Bruker Avance spectrometers equipped with a 5 mm single-axis *Z*-gradient quattro nucleus probe, operating at 300 and 400 MHz for ^1^H and at 75 and 100 MHz for ^13^C. The spectrometer was running TOPSPIN NMR system software (Version 2.0). Chemical shifts (δ) are reported in parts per million (ppm), peak positions relative to Me_4_Si (0.00 ppm) for ^1^H and ^13^C NMR spectra. ^19^F NMR spectra were recorded on a Bruker Avance-400 spectrometer operating at 376 MHz, and chemical shifts were referenced to hexafluorobenzene at 161.7 ppm. Abbreviations used for splitting patterns are: s, singlet; d, doublet; dd, doublet of doublet; t, triplet; q, quartet; p, pentet; m, multiplet. Mass spectra were recorded at the School of Chemistry, University of Manchester using Micromass PLATFORM II (ES) and Thermo Finnigan MAT95XP (Accurate mass and GCMS) instruments. IR spectroscopy was performed on the solid and liquid states using a JASCO Fourier transform infrared spectrophotometer. The purity of final compounds **2–7** is >95% as shown by HPLC-MS (Table S5).

### General Procedure for the Synthesis of Amides (**13**, **14**)

Thionyl chloride (6.0 mmol) was added dropwise to 4-fluoro-2-methylbenzoic acid **9** (1.0 mmol) for the synthesis of **13**, and 5-fluoro-2-methylbenzoic acid **10** for the synthesis of **14** (1.0 mmol) at 0 °C. The reaction mixture was stirred for 30 min at 0 °C and then heated at reflux overnight at 45 °C. Thionyl chloride was removed on a rotary evaporator and CH_2_Cl_2_ (20 mL) was added and stirred at room temperature for 15 min. Diethylamine (8.0 mmol) was added dropwise to the reaction mixture at 0 °C and stirred overnight. Completion of the reaction was monitored by TLC (EtOAc-hexane; 3:7 v/v). The reaction mixture was quenched with water and extracted with EtOAc. The organic layer was washed with 2 M aq. HCl, dried with dry MgSO_4_, and evaporated.

#### *N,N*-Diethyl-4-fluoro-2-methylbenzamide (**13**)

Amide (**13**) was obtained as a viscous oil (0.98 g, 91%). IR 2975 (ArCH), 2875 (CH_3_, CH_2_), 1625 (C=O) cm^–1^. ^1^H NMR (400 MHz, CDCl_3_) δ 7.12 (dd ∼ t, ^3^*J*_HH_ = 7.8 Hz, ^4^*J*_HF_ = 6.0 Hz, 1H, H6), 6.94–6.84 (m, 2H, H3, 5), 3.55 (br s, 2H, NCH_2_), 3.10 (q, ^3^*J*_HH_ = 7.0 Hz, 2H, NCH_2_), 2.27 (s, 3H, ArCH_3_), 1.24 (t, ^3^*J*_HH_ = 7.0 Hz, 3H, NCH_2_CH_3_), 1.02 (t, ^3^*J*_HH_ = 7.0 Hz, 3H, NCH_2_CH_3_); ^13^C-NMR (100 MHz, CDCl_3_) (assignments made using DEPT-135) δ170.1 (C=O), 162.5 (d, ^1^*J*_CF_ = 246 Hz, C4), 136.8 (d, ^3^*J*_CF_ = 7.9 Hz, C2), 133.2 (d, ^4^*J*_CF_ = 3.4 Hz, C1), 127.2 (d, ^3^*J*_CF_ = 8.4 Hz, C6), 117.2 (d, ^2^*J*_CF_ = 21.2 Hz, C3), 112.7 (d, ^2^*J*_CF_ = 21.5 Hz, C5), 42.7 (NCH_2_), 38.9 (NCH_2_), 18.9 (d, ^4^*J*_CF_ = 1.4 Hz, Ar-CH_3_), 14.0 (NCH_2_CH_3_), 12.8 (NCH_2_CH_3_); ^19^F NMR (^1^H-decoupled, 376 MHz, CDCl_3_) δ −116.4 (s, F).

#### *N,N*-Diethyl-5-fluoro-2-methylbenzamide (**14**)

Amide (**14**) was obtained as a viscous oil (0.95 g, 70%). IR 2941 (ArCH), 2830 (CH_3_, CH_2_), 1611 (C=O) cm^–1^. ^1^H NMR (400 MHz, CDCl_3_) δ 7.16 (dd, ^3^*J*_HH_ = 8.4 Hz, ^4^*J*_HF_ = 5.4 Hz, 1H, H3), 6.93 (td, ^3^*J*_HH_ = ^3^*J*_HF_ = 8.4 Hz, ^4^*J*_HH_ = 2.7 Hz 1H, H4), 6.87 (dd, ^3^*J*_HF_ = 8.6 Hz, ^4^*J*_HH_ = 2.6 Hz, 1H, H6), 3.68 (br s, 1H, NCH_2_), 3.45 (br s, 1H, NCH_2_), 3.12 (q, ^3^*J*_HH_ = 7.2 Hz, 2H, NCH_2_), 2.23 (s, 3H, ArCH_3_), 1.25 (t, ^3^*J*_HH_ = 7.5 Hz, 3H, NCH_2_*CH*_*3*_), 1.04 (t, ^3^*J*_HH_ = 7.5 Hz, 3H, NCH_2_CH_3_); ^13^C NMR (100 MHz, CDCl_3_) (assignments made using DEPT-135) δ 169.3 (d, ^4^*J*_CF_ = 2.0 Hz, C=O), 160.8 (d, ^1^*J*_CF_ = 244.0 Hz, C5), 138.4 (d, ^3^*J*_CF_ = 6.6 Hz, C1), 131.8 (d, ^3^*J*_CF_ = 7.8 Hz, C3), 129.5 (d, ^4^*J*_CF_ = 3.5 Hz, C2), 115.3 (d, ^2^J_CF_, = 20.7 Hz, C4), 112.4 (d, ^2^*J*_CF_ = 22.4 Hz, C6), 42.5 (NCH_2_), 38.7 (NCH_2_), 18.0 (Ar-CH_3_), 13.9 (NCH_2_CH_3_), 12.8 (NCH_2_CH_3_); ^19^F NMR (^1^H-decoupled, 376 MHz, CDCl_3_) δ −120.2 (s, F).

### General Procedure for the Synthesis of Isoquinoline-1-ones (**2**–**8**)

A solution of the appropriate benzonitrile (**15–18**) (1.5 mmol) in dry THF (10.0 mL) was added dropwise to a solution of n-BuLi (3.8 mmol, 2.5 M) in dry THF (10 mL) for the synthesis of **2**–**4** or LDA (4.0 mmol, 1.0 M) in dry THF (10 mL) for the synthesis of **5**–**8** at **–**78 °C. The reaction mixture was stirred overnight (15 h). Completion of the reaction was monitored by TLC (MeOH-CH_2_Cl_2_; 1:99 v/v).

#### 6-Fluoro-3-(3-methoxyphenyl)isoquinolin-1(2*H*)-one (**2**)

Compound (**2**) was purified with silica gel column chromatography eluting with CH_2_Cl_2_ to give an off-white solid (**2**) (0.073 g, 57%). Mp 219–221 °C. IR 3128 (NH), 2932 (ArCH), 2842 (CH_3_), 1661 (C=O) cm^–1^. ^1^H NMR (400 MHz, DMSO-*d*_6_) δ 11.58 (br s, 1H, NH), 8.26 (dd, ^3^*J*_HH_ = 8.7 Hz, ^4^*J*_HF_ = 6.3 Hz, 1H, H8), 7.51 (dd, ^3^*J*_HF_ = 10.2 Hz, ^4^*J*_HH_ = 1.5 Hz, 1H, H5), 7.41 (t, ^3^*J*_HH_ = 8.0 Hz, 1H, H5′), 7.37–7.28 (m, 3H, H2′, 6′, 7), 7.03 (br d, ^3^*J*_HH_ = 7.5 Hz, 1H, H4′), 6.95 (br s, 1H, H4), 3.85 (s, 3H, OCH_3_); ^13^C NMR (100 MHz, DMSO-*d*_6_) (assignments made using DEPT-135) δ 164.6 (d, ^1^*J*_CF_ = 247.5 Hz, C6), 162.0 (C3′), 159.4 (C=O), 141.3 (C1′), 140.2 (d, ^3^*J*_CF_ = 10.7 Hz, C10), 134.9 (C3), 130.1 (d, ^3^*J*_CF_ = 10.4 Hz, C8), 129.9 (C5′, 6′), 121.8 (C9), 115.6 (C4′), 114.8 (d, ^2^*J*_CF_ = 23.8 Hz, C7), 111.8 (C2′), 111.2 (d, ^2^*J*_CF_ = 21.8 Hz, C5), 102.7 (d, ^4^*J*_CF_ = 3.2 Hz, C4), 55.3 (OCH_3_); ^19^F NMR (^1^H-decoupled, 376 MHz, DMSO-*d*_6_) δ −109.5 (s, F); ^19^F NMR (^1^H-coupled, 376 MHz, DMSO-*d*_6_) δ −109.5 (td, ^3^*J*_t_ = 9.0 Hz, ^4^*J*_d_ = 6.0 Hz, F). MS(ES) *m*/*e* [M + H]^+^ 270.1. Accurate mass calcd for C_16_H_13_FNO_2_: 270.0925. Found: 270.0920.

#### 6-Fluoro-3-(4-methoxyphenyl)isoquinolin-1(2*H*)-one (**3**)

Compound (**3**) was purified using silica gel column chromatography eluting with CH_2_Cl_2_ to give a pale yellow solid (**3**) (0.054 g, 42%). Mp 215–217 °C. IR 3157 (NH), 2959 (ArCH), 2836 (CH_3_), 1628 (C=O) cm^–1^. ^1^H NMR (400 MHz, DMSO-*d*_6_) δ 11.51 (br s, 1H, NH), 8.24 (dd, ^3^*J*_HH_ = 8.7 Hz, ^4^*J*_HF_ = 6.2 Hz, 1H, H8), 7.74 (br d, ^3^*J*_HH_ = 8.7 Hz, 2H, H2′, 6′), 7.47 (dd, ^3^*J*_HF_ = 10.1 Hz, ^4^*J*_HH_ = 2.3 Hz, 1H, H5), 7.28 (td, ^3^*J*_HF_ = ^3^*J*_HH_ = 8.4 Hz, ^4^*J*_HH_ = 2.2 Hz, 1H, H7), 7.06 (br d, ^3^*J*_HH_ = 8.7 Hz, 2H, H3′, 5′), 6.83 (br s, 1H, H4), 3.82 (s, 3H, OCH_3_); ^13^C NMR (100 MHz, DMSO-*d*_6_) (assignments made using DEPT-135) δ 164.6 (d, ^1^*J*_CF_ = 247.7 Hz, C6), 162.1 (C4′), 160.3 (C=O), 141.3 (C3), 140.5 (d, ^3^*J*_CF_ = 10.8 Hz, C10), 130.1 (d, ^3^*J*_CF_ = 10.1 Hz, C8), 128.1 (C2′, 6′), 125.8 (C1′), 121.4 (C9), 114.4 (d, ^2^*J*_CF_ = 23.5 Hz, C7), 114.2 (C3′, 5′), 110.9 (d, ^2^*J*_CF_ = 21.6 Hz, C5), 101.5 (d, ^4^*J*_CF_ = 3.0 Hz, C4), 55.3 (OCH_3_); ^19^F NMR (^1^H-decoupled, 376 MHz, DMSO-*d*_6_) δ −109.6 (s, F). MS(ES) *m*/*e* [M + H]^+^ 270.1. Accurate mass calcd for C_16_H_13_FNO_2_: 270.0925. Found: 270.0923.

#### 6-Fluoro-3-(3-fluorophenyl)isoquinolin-1(2*H*)-one (**4**)

Compound (**4**) was purified using silica gel column chromatography eluting with CH_2_Cl_2_ to give a yellow solid (**4**) (0.120 g, 43%). Mp 280–283 °C. IR 3128 (NH), 2982 (ArCH), 1632 (C=O) cm^–1^. ^1^H NMR (400 MHz, DMSO-*d*_6_) δ 11.64 (br s, 1H, NH), 8.26 (dd, ^3^*J*_HH_ = 8.7 Hz, ^4^*J*_HF_ = 6.0 Hz, 1H, H8), 7.68–7.62 (m, 2H, H7, 6′), 7.60–7.48 (m, 2H, H5, 5′), 7.37 (dd, ^3^*J*_HH_ = 8.7 Hz, ^4^*J*_HH_ = 2.4 Hz, H2′), 7.31–7.28 (m, 1H, H4′), 7.0 (br s, 1H, H4); ^13^C NMR (100 MHz, (CD_3_)_2_SO) (assignments made using DEPT-135) δ164.6 (d, ^1^*J*_CF_ = 248.0 Hz, C6), 162.2 (d, ^1^*J*_CF_ = 242.6 Hz, C3′), 162.0 (C=O), 140.1 (d, ^4^*J*_CF_ = 3.5 Hz, C3), 140.0 (d, ^3^*J*_CF_ = 4.8 Hz, C10), 135.8 (d, ^3^*J*_CF_ = 8.3 Hz, C1′), 130.9 (d, ^3^*J*_CF_ = 8.6 Hz, C5′), 130.2 (d, ^3^*J*_CF_ = 10.0 Hz, C8), 122.9 (d, ^4^*J*_CF_ = 2.8 Hz, C6′), 122.0 (C9), 116.3 (d, ^2^*J*_CF_ = 21.1 Hz, C7), 115.2 (d, ^2^*J*_CF_ = 23.5 Hz, C4′), 113.7 (d, ^2^*J*_CF_ = 23.5 Hz, C5), 111.4 (d, ^2^*J*_CF_ = 21.5 Hz, C2′), 103.4 (d, ^4^*J*_CF_ = 3.1 Hz, C4); ^19^F NMR (^1^H-decoupled, 376 MHz, DMSO-*d*_6_) δ −109.3 (s, F at C6), −114.7 (s, F at C3′). MS(ES) *m*/*e* [M + H]^+^ 258.1. Accurate mass calcd for C_15_H_10_F_2_NO: 258.0725. Found: 258.0725.

#### 6-Fluoro-3-(4-fluorophenyl)isoquinolin-1(2*H*)-one (**5**)

Compound (**5**) was purified using silica gel column chromatography eluting with CH_2_Cl_2_ to give a white solid (**5**) (0.085 g, 66%). Mp 287–289 °C. IR 3126 (NH), 2924 (ArCH), 1664 (C=O) cm^–1^. ^1^H NMR (400 MHz, DMSO-*d*_6_) δ 11.62 (br s, 1H, NH), 8.26 (dd, ^3^*J*_HH_ = 8.7 Hz, ^4^*J*_HF_ = 6.0 Hz, 1H, H8), 7.83 (dd, ^3^*J*_HH_ = 9.0 Hz, ^4^*J*_HF_ = 5.4 Hz, 2H, H2′, 6′), 7.50 (dd, ^3^*J*_HF_ = 10.0 Hz, ^4^*J*_HH_ = 2.6 Hz 1H, H5), 7.35 (t, ^3^*J*_HH_ = ^3^*J*_HF_ = 8.9 Hz, 1H, H3′, 5′), 7.32 (td, ^3^*J*_HF_ = ^3^*J*_HH_ = 8.7 Hz, ^4^*J*_HH_ = 2.4 Hz, 1H, H7), 6.88 (br s, 1H, H4); ^13^C NMR (100 MHz, DMSO-*d*_6_) (assignments made using DEPT-135) δ 164.8 (d, ^1^*J*_CF_ = 248.0 Hz, C6), 162.9 (d, ^1^*J*_CF_ = 245.6 Hz, C4′), 162.1 (C=O), 140.6 (C3), 140.3 (d, ^3^*J*_CF_ = 10.8 Hz, C10), 130.2 (d, ^3^*J*_CF_ = 10.5 Hz, C8), 130.1 (^4^*J*_CF_ = 1.9 Hz, C1′), 129.2 (d, ^3^*J*_CF_ = 8.5 Hz, C2′, 6′), 121.7 (^4^*J*_CF_ = 1.1 Hz, C9), 115.7 (d, ^2^*J*_CF_ = 21.8 Hz, C3′, 5′), 114.9 (d, ^2^*J*_CF_ = 23.6 Hz, C7), 111.2 (d, ^2^*J*_CF_ = 21.8 Hz, C5), 102.7 (d, ^4^*J*_CF_ = 2.8 Hz, C4); ^19^F NMR (^1^H-decoupled, 376 MHz, DMSO-*d*_6_) δ −109.4 (s, F at C6), −114.1 (s, F at 4′). MS(ES) *m*/*e* [M + H]^+^ 258.1. Accurate mass calcd for C_15_H_10_F_2_NO: 258.0725. Found: 258.0725.

#### Fluoro-3-(3-methoxyphenyl)isoquinolin-1(2*H*)-one (**6**)

Compound (**6**) was purified using silica gel column chromatography eluting with CH_2_Cl_2_ to give a white solid (**6**) (0.070 g, 54%). Mp 229–231 °C. IR 3154 (NH), 2994 (ArCH), 2842 (CH_3_), 1658 (C=O) cm^–1^. ^1^H NMR (400 MHz, DMSO-*d*_6_) δ 11.67 (br s, 1H, NH), 7.86 (dd, ^3^*J*_HF_ = 9.0 Hz, ^4^*J*_HH_ = 2.7 Hz, 1H, H8), 7.83 (dd, ^3^*J*_HH_ = 8.7 Hz, ^4^*J*_HF_ = 5.4 Hz, 1H, H5), 7.64 (td, ^3^*J*_HH_ = ^3^*J*_HF_ = 8.7 Hz, ^4^*J*_HH_ = 2.7 Hz, 1H, H6), 7.44-7.34 (m, 3H, H4′, 5′, 6′), 7.02 (brs, 2H, H2′, 4), 3.85 (s, 3H, OCH_3_); ^13^C NMR (100 MHz, DMSO-*d*_6_) (assignments made using DEPT-135) δ 162.0 (d, ^4^*J*_CF_ = 2.9 Hz, C=O), 160.5 (d, ^1^*J*_CF_ = 243.3, C7), 159.4 (C3′), 139.2 (C3), 135.1 (C1′), 134.8 (d, ^4^*J*_CF_ = 2.9 Hz, C10), 129.9 (C5′), 129.7 (d, ^3^*J*_CF_ = 7.9 Hz, C5), 126.3 (d, ^3^*J*_CF_ = 7.9 Hz, C9), 121.4 (d, ^2^*J*_CF_ = 23.6 Hz, C8), 118.9 (C6′), 115.4 (C4′), 111.7 (C2′), 111.3 (d, ^2^*J*_CF_ = 22.3 Hz, C6), 102.9 (C4), 55.3 (OCH_3_); ^19^F NMR (^1^H-decoupled, 376 MHz, DMSO-*d*_6_) δ −115.9 (s, F); ^19^F NMR (^1^H-coupled, 376 MHz, DMSO-*d*_6_) δ −115.9 (td, ^3^*J*_t_ = 9.0 Hz, ^4^*J*_d_ = 5.3 Hz, F). MS(ES) *m*/*e* [M + H]^+^ 268.1. Accurate mass calcd for C_16_H_11_FNO_2_: 268.0779. Found: 268.0773.

#### 7-Fluoro-3-(4-methoxyphenyl)isoquinolin-1(2*H*)-one (**7**)

Compound (**7**) was purified using silica gel column chromatography eluting with CH_2_Cl_2_ to give a white solid (**7**) (0.075 g, 58%). Mp 272–273 °C. IR 3154 (NH), 2932 (ArCH), 2838 (CH_3_), 1614 (C=O) cm^–1^. ^1^H NMR (400 MHz, DMSO-*d*_6_) δ 11.59 (br s, 1H, NH), 7.84 (dd, ^3^*J*_HF_ = 9.3 Hz, ^4^*J*_HH_ = 2.4 Hz, 1H, H8), 7.79 (dd, ^3^*J*_HH_ = 8.7 Hz, ^4^*J*_HF_ = 3.3 Hz, 1H, H5), 7.74 (br d, ^3^*J*_HH_ = 8.7 Hz, 2H, H2′,6′), 7.61 (td, ^3^*J*_HF_ = ^3^*J*_HH_ = 8.7 Hz, ^4^*J*_HH_ = 2.7 Hz, 1H, H6), 7.04 (br d, ^3^*J*_HH_ = 8.7 Hz, 2H, H3′, 5′), 6.90 (br s, 1H, H4), 3.82 (s, 3H, OCH_3_); ^13^C NMR (100 MHz, DMSO-*d*_6_) (assignments made using DEPT-135) δ 162.1 (d, ^4^*J*_CF_ = 3.3 Hz, C=O), 160.3 (d, ^1^*J*_CF_ = 242.8 Hz, C7), 160.1 (C4′), 139.3 (C3), 135.0 (C10), 129.3 (d, ^3^*J*_CF_ = 7.8 Hz, C5), 128.0 (C2′, 6′), 126.0 (C1′), 125.8 (d, ^3^*J*_CF_ = 7.0 Hz, C9), 121.3 (d, ^2^*J*_CF_ = 23.4 Hz, C8), 114.2 (C3′, 5′), 111.2 (d, ^2^*J*_CF_ = 22.3 Hz, C6), 101.6 (C4), 55.3 (OCH_3_); ^19^F NMR (^1^H-decoupled, 376 MHz, DMSO-*d*_6_) δ −116.6 (s, F). MS(ES) *m*/*e* [M + H]^+^ 270.1. Accurate mass calcd for C_16_H_13_FNO_2_: 270.0925. Found: 270.0920.

#### 7-Fluoro-3-(3-fluorophenyl)isoquinolin-1(2*H*)-one (**8**)

Compound (**8**) was purified using silica gel column chromatography eluting with CH_2_Cl_2_ to give a white solid (**8**) (0.052 g, 54%). Mp 294–296 °C. IR 3155 (NH), 2990 (ArCH), 1660 (C=O) cm^–1^. ^1^H NMR (400 MHz, DMSO-*d*_6_) δ 11.69 (br s, 1H, NH), 7.88–7.82 (m, 1H, H8), 7.81 (dd, ^3^*J*_HH_ = 8.4 Hz, ^4^*J*_HF_ = 5.4 Hz, 3H, H2′, 6′, 5), 7.62 (td, ^3^*J*_HF_ = ^3^*J*_HH_ = 8.7 Hz, ^4^*J*_HH_ = 3.0 Hz, 1H, H6), 7.33 (t, ^3^*J*_HF_ = ^3^*J*_HH_ = 8.9 Hz, 2H, H3′, 5′), 6.94 (br s, 1H, H4); ^13^C NMR (100 MHz, DMSO-*d*_6_) (assignments made using DEPT-135) δ 162.7 (d, ^1^*J*_CF_ = 245.5 Hz, C7), 162.0 (d, ^4^*J*_CF_ = 3.4 Hz, C=O), 160.5 (d, ^1^*J*_CF_ = 243.4 Hz, C4′), 138.3 (C3), 134.8 (d, ^4^*J*_CF_ = 1.6 Hz, C10), 130.3 (d, ^4^*J*_CF_ = 2.9 Hz, C1′), 129.6 (d, ^3^*J*_CF_ = 7.9 Hz, C5), 129.0 (d, ^3^*J*_CF_ = 8.3 Hz, C2′, 6′), 126.2 (d, ^3^*J*_CF_ = 8.3 Hz, C9), 121.4 (d, ^2^*J*_CF_ = 23.4 Hz, C6), 115.7 (d, ^2^*J*_CF_ = 21.8 Hz, C3′, 5′), 111.3 (d, ^2^*J*_CF_ = 22.3 Hz, C8), 102.8 (C4); ^19^F NMR (^1^H-decoupled 376 MHz, DMSO-*d*_6_): δ −114.5 (s, F at C4′), −115.8 (s, F at C7). MS(ES) *m*/*e* [M + H]^+^ 258.1. Accurate mass calcd for C_15_H_10_F_2_NO: 258.0725. Found: 258.0724.

### Computational Docking

Chains A and B of tubulin from its colchicine complex crystal structure (PDB code 4O2B)^45^ were prepared using Molecular Operating Environment (MOE)^46^ and used for computational docking. Docking was performed using the OpenEye software suite.^47^ Omega classic was used to create a 3D structure of the compounds using a maximum number of conformations of 100 for each compound. The colchicine site of tubulin was prepared for docking using the *make_receptor* module. The FRED module was used to dock the compounds using the Chemgauss4 scoring function. The best 20 poses for each compound were visualized using Vida 4.4.0 and MOE 2020.09. This docking protocol was able to closely reproduce the binding mode of colchicine as the top-ranked pose (Figure S25).

### Molecular Dynamics Simulations

Molecular dynamics simulations of tubulin–ligand complexes were conducted using the AMBER 19 package.^45^ Atomic partial charges of the ligands were assigned via the AM1-BCC method implemented in the *antechamber* module of AMBER. The *gaff2* and *ff14SB* force fields were used to describe the ligands and the receptor, respectively.^48,49^ The GTP cofactor was modeled using the parameters of Meagher et al.^50^ and *gaff2*. The systems were solvated in an octahedral TIP3P water box^51^ that extends at least 15 Å from the protein–ligand surface. Sodium and chloride counterions were added to neutralize the system and model a salt concentration of 0.15 mM. This led to ∼43,000 water molecules for each simulation system. The generated topology file was edited with the *parmed* module of AMBER 19 to repartition the mass of heavy atoms into the bonded hydrogen atoms. The new topology file was designed to use hydrogen mass repartitioning (HMR)^52^ in which the time step of the simulation could be increased to 4 fs. The nonbonded cutoff of 9.0 Å was used, along with the particle mesh Ewald (PME) method for long-range electrostatic interactions.

MD simulations were performed using the *pmemd.cuda* module of AMBER 19. For simulation of compound **4** bound to tubulin, the system was energy-minimized and then gradually heated from 0 to 300 K over 500 ps in the NVT ensemble using the Langevin thermostat.^53^ Covalent bonds to hydrogen were restrained by the SHAKE algorithm. Water and ions were relaxed over 5 ns while the protein–ligand complex was restrained with a weight of 10 kcal/(mol Å). The restraints were released gradually over 5 ns, and the system was equilibrated for 10 ns in an NPT ensemble at 300 K and 1 atm using the Berendsen barostat.^54^ Production simulations were performed for 20 ns in an NPT ensemble, during which the snapshots were sampled every 10 ps. For simulation of the remaining compounds in complex with tubulin, starting from the 30 ns MD pose of **4**/tubulin, these compounds were reequilibrated for 20 ns, followed by production dynamics for 10 ns.

For all compound-protein systems, the MM/GBSA method was applied to the final 10 ns to compute approximate binding free energies. These calculations were performed using the MMPBSA.py tool of AMBER 19. The internal and external dielectric constants were set to 1.0 and 80.0, respectively. The ionic strength was set to 0.15 mM. MM/GBSA calculations were performed using 100 snapshots/compound. The electrostatic contribution to binding free energy Δ*G*_el_ was a sum of electrostatic protein–ligand and solvation components; the nonelectrostatic contribution Δ*G*_nonel_ was a sum of protein–ligand van der Waals and nonelectrostatic solvation terms.

### Cell Culture

All cell lines used in the study were human-derived and purchased from ATCC (Manassas, USA). Breast adenocarcinoma MCF-7 and MDA-MB-231 cell lines were cultured in DMEM. Hepatocellular carcinoma HepG2 and SNU423 cell lines were grown in MEME combined with 1% (v/v) nonessential amino acids and RPMI supplemented with 2 mM glutamine, respectively. Lung adenocarcinoma A549 and colorectal carcinoma HCT116 cells were grown in RPMI (+2 mM glutamine). All previous media were further supplemented with 10% heat-inactivated fetal bovine serum. The immortalized, normal liver THLE-3 cells were grown in BEGM (Lonza, Cat# CC-3170) onto precoated vessels according to ATCC’s instructions. Human umbilical vein endothelial cells (HUVECs) were cultured in EGM-2 (Lonza, Cat# CC-3162). All of the cells were maintained at 37 °C and 5% CO_2_ in a humidified incubator.

### Preparation of Compounds for Cell Treatment

The stock solutions of compounds **2**–**8** were made in DMSO at 40 mM and stored in single-use aliquots at −20 °C. Directly before each experiment, compounds were diluted in culture medium to the required concentrations where DMSO was ≤0.3%.

### SRB Assay

Screening of the antiproliferative activity of 3-arylisoquinolinones was performed using the SRB assay as previously described.^56^ Exponentially growing HepG2, SNU423, MDA-MB-231, MCF-7, A549, HCT116, THLE-3, and HUVECs were seeded in 96-well plates at densities of 3000, 2000, 1500, 2500, 1000, 1000, 4000, and 4000 cells/well, respectively. These densities were experimentally selected so that the DMSO-treated cells (control) were approximately 80–90% confluent at the assay’s endpoint, and their optical density (OD) values fell within the assay’s linear range. After overnight incubation, the cells were treated with a range of concentrations of the respective compounds for 96 h, 24 h followed by 72 h compound-free incubation or 24 h (for HUVECs only). After treatment, cell monolayers were fixed with 10% (w/v) trichloroacetic acid for 1 h at 4 °C, washed, and dried. Then, the cells were exposed to 0.4% (w/v) SRB dye (prepared in 1% (v/v) acetic acid; Botium, Cat# 80100) for 15 min followed by several 1% acetic acid washing and then drying. Finally, the cellular protein-bound SRB was thoroughly dissolved in 10 mM Tris base, and the OD values were read at 510 nm using a plate reader (μQuant Microplate Spectrophotometer, Biotek, U.K.) coupled with Gen5 software (BioTek, U.K.). Compound-induced changes in cell growth were inferred from changes in OD values. Cell growth was calculated using the following formula: cell growth (%) = (OD_test_/OD_control_) × 100. The IC_50_ value, the concentration at which a compound inhibits cell growth by 50% of that of the control, was determined graphically from dose–response curves created using the four-parameter nonlinear regression analysis in GraphPad Prism 8.

### Clonogenic Assay

The ability of potential anticancer agents to prevent unlimited cell division and induce reproductive cell death was assessed using clonogenic assay as previously described.^57^ In brief, HepG2 cells were seeded into six-well plates at a density of 1000 single cells/well and incubated for 10–12 h to adhere. The cells were then treated with DMSO (control) or a range of concentrations of **2–6** for 14 days. Afterward, the produced colonies were washed with phosphate-buffered saline (PBS), fixed with 70% ethanol for 20 min, and stained with 0.5% (w/v) methylene blue (prepared in 70% methanol). Following washing with water and drying, colonies (≥50 cells) were manually counted. The colony-forming efficiency (CFE) of cells was calculated by dividing the number of colonies formed by the number of cells seeded for each condition. The surviving fraction was determined by dividing the CFE of the treated cells by CFE of the control cells. A survival curve was generated by plotting the log of drug concentrations versus surviving fraction.

### Tubulin Polymerization Assay

The cell-free tubulin polymerization experiment was performed using the Tubulin Polymerization Assay Kit according to the manufacturer’s instructions (Millipore, Cat# 17-10194). The light absorbed by microtubules, which were formed upon tubulin polymerization, is directly proportional to the polymer mass. Briefly, polymerization buffer containing 1 mM GTP was used to dilute the purified bovine tubulin (making 60 μM), DMSO (negative control), paclitaxel (tubulin-polymerizing agent), nocodazole (tubulin-depolymerizing agent), as well as **4**. In a half area, 96-well plate kept on ice, tubulin was gently mixed with DMSO or the test agents so that the final concentrations of paclitaxel and nocodazole were 10 μM, and those of **4** were 5, 10, and 20 μM. Immediately, the plate was placed in a 37 °C-prewarmed plate reader (BMG LABTECH FLUOstar Omega, Germany) and the absorbance was measured at 1 min intervals for 60 min at a wavelength of 350 nm. The delta absorbance (Δ*A*) was calculated by subtracting the absorbance values of zero time from those of the subsequent time points and then plotted versus time.

### Cell Cycle Analysis

HepG2 cells (5 × 10^5^) were seeded into 6 cm cell culture dishes and allowed to attach overnight. Then, the cells were exposed to DMSO, **4** (1, 2, 5 μM), or **5** (25 μM) as two sets. One set was subjected to 24 h treatment, whereas the other was treated for 24 h followed by a 24 h compound-free period. Floating and adhesive cells were then collected, washed with PBS, and fixed by vortexing with ice-cold 70% ethanol and following shaking onto ice for 30 min. The ethanol–cell suspension was washed twice with PBS and centrifuged at a high speed (2000*g*, 10 min) after each wash. Next, the cell pellet was incubated with DNA staining buffer made of 400 μL of PBS, 50 μL of 1 mg/mL RNase A (Thermo Fisher Scientific, Cat# EN0531), and 50 μL of 400 μg/mL propidium iodide (PI; Molecular Probes, Cat# P3566) for 30 min at 37 °C in the dark. Finally, the PI fluorescence data of 10 000 single cells were acquired (see Figure S26 for gating) using an FACSCanto II flow cytometer (BD Biosciences; Flow Cytometry Facility, University of Manchester). Cell cycle profiles were analyzed using FlowJo_V10 software, where the Watson model was applied on histograms to calculate the percentage of cell population in each cell cycle phase.

### Annexin V/PI Apoptosis Assay

SNU423 (2 × 10^5^) cells were seeded into 6 cm dishes and incubated overnight. The culture medium was then changed with a fresh one (to get rid of any floating, potentially dead cells) followed by 24 h treatment with **4** (0.5, 1 μM). Floating and attached cells were then collected, washed with cold PBS, and washed with Annexin V binding buffer (ABB; BD Pharmingen, Cat# 556454). Next, the cell pellets were incubated with 2% (v/v) APC-conjugated Annexin V (prepared in ABB; BD Pharmingen, Cat# 550474) for 10 min at RT in the dark. Following washing with ABB, the cell pellets were resuspended in 300 μL of 0.5 μg/mL PI (prepared in ABB), kept on ice, and protected from light. Finally, the cells were promptly analyzed with flow cytometry, where the fluorescence data were collected from 10 000 single cells following gating (see Figure S27) and plotted onto quadrant graphs to determine the percentages of different cell populations. The bottom left and top left quadrants indicate live (Annexin Vl^ow^/PI^low^) and necrotic (Annexin Vl^ow^/PI^high^) cells, respectively. Further, the bottom right and top right quadrants represent early apoptotic (Annexin Vl^high^/PI^low^) and late apoptotic/necrotic (Annexin Vl^high^/PI^high^) cells, respectively.

### Cytoskeleton Immunofluorescence

HUVECs (2 × 10^4^) were allowed to adhere overnight into eight-well glass slides (Ibidi, Cat# 80841) followed by 24 h treatment with colchicine (0.1 μM, positive control) and **4** (0.5, 1 μM). Throughout the next steps, all of the reagents were removed by washing, flicking, and gently dabbing the slide against a tissue paper. The cells were then washed with PBS containing calcium and magnesium (Sigma, Cat# D1283), fixed with 10% formalin solution (Sigma, Cat# HT5014) for 15 min, and permeabilized with 0.2% (v/v) Triton X-100 for 5 min. Next, the cells were exposed to 1 h incubation with blocking buffer made of 10% (v/v) normal goat serum (Vector Laboratories, Cat# S-1000), 1% (w/v) bovine serum albumin (Sigma, Cat# A9418), and 0.3 M glycine in PBS-T (0.05% Tween-20 in PBS). The cells were then incubated with rabbit α-tubulin primary antibody (1:1000 in the blocking buffer, Abcam, Cat# ab52866) for 2 h at RT after three times washing with PBS-T, each for 5 min. Afterward, the cells were co-incubated with cyanine 5 goat anti-rabbit IgG (1:1000, Invitrogen, Cat# A10523) and the F-actin specific stain Phalloidin-Atto 488 (1:500, Sigma, Cat# 49409) for 1 h in the dark at RT and then washed with PBS-T. The nuclei were stained with DAPI (1 μg/mL in PBS) for 5 min followed by mounting coverslips on slides using Dako mounting medium (Cat# S3023). Finally, the cytoskeleton was visualized at 63x, oil-based magnification using fluorescence, inverted microscope (Leica DMI6000B, Leica Microsystems). Images were then processed using Fiji ImageJ software.

### Endothelial Cell Tube Formation Assay

Matrigel (Corning, Cat# 356237) was gently added into 96-well plates at a volume of 70 μL/well and allowed to gel at 37 °C for 30 min. A mixture of HUVECs (25 × 10^3^) and DMSO, **4** (0.5, 1, 2 μM), or **5** (25 μM) was then dispensed over the gels and incubated for 16 h. Next, the wells were filled with PBS and covered with a coverslip to diminish the meniscus effect, allowing proper examination of the endothelial tube networks under a light microscope (Optika XDS-3, Italy). Bright-field images of 4x magnification were captured and then processed using Fiji ImageJ software. For the assessment of endothelial tube disruption, HUVECs at a density of 25 × 10^3^ cells/well were seeded onto Matrigel and incubated for 4 h to align into tubes. The cells were then treated with DMSO, **4**, or **5** for 16 h prior to the microscopic examination as mentioned above.

### Statistics

Statistical analyses were carried out using GraphPad Prism version 8.0 (San Diego, CA). The data were presented as the mean ± SEM of a minimum of three independent experiments. One-way analysis of variance (ANOVA) followed by Dunnett’s or Tukey’s multiple comparisons test was employed to determine the significance level of differences among groups (versus control in Dunnett’s). Differences with *p* < 0.05 were considered statistically significant.

## Supporting Information Available

^1^H, ^13^C, and ^19^F NMR spectra of all compounds; dose–response curves for 3-arylisoquinolinones in a panel of six cancer cell lines;NCI one-dose and five-dose screens of 3-arylisoquinolinones; potential energy surfaces for 3-arylisoquinolinones; cell gating for cell cycle and apoptosis analyses by flow cytometry; COMPARE analysis results of compound **4** against the Standard NCI Database; tables from the computational docking studies; and HPLC-MS purity analysis of compounds **2**–**7**(PDF)
Molecular formula strings and some biology data (CSV)

## Abbreviations Used

ABB: Annexin V binding buffer
CFE: colony-forming efficiency
CS: compare solution
HUVECs: human umbilical vascular endothelial cells
IC_50_: inhibitory concentration 50
MTAs: microtubule-targeting agents
NCI: National Cancer Institute
NQO2: *N*-ribosyl dihydronicotinamide (NRH): quinone oxidoreductase 2
OD: optical density
PBS: phosphate-buffered saline
RT: room temperature
SRB: sulforhodamine B
