# Supplementary material for: SAR of Novel 3-Arylisoquinolinones: meta-Substitution on the Aryl Ring Dramatically Enhances Antiproliferative Activity through Binding to Microtubules
Source: J Med Chem. 2022 Mar 15;65(6):4783–97. doi: 10.1021/acs.jmedchem.1c01936 (PMC9098178; doi:10.1021/acs.jmedchem.1c01936)
Supplement: Supplementary file 1 — jm1c01936_si_001.pdf [file jm1c01936_si_001.pdf]

Supplementary Information

SAR of Novel 3-Arylisoquinolinones: *meta*- Substitution on the Aryl Ring Dramatically Enhances Antiproliferative Activity through Binding to Microtubules

Mai A Elhemely[a, b], Asma A Belgath[a], Sherihan El-Sayed[a,c], Kepa K Burusco[a], Manikandan Kadirvel[a], Annalisa Tirella[a,d], Katherine Finegan[a], Richard A Bryce[a], Ian J Stratford[a] and Sally Freeman[a].

[a]Division of Pharmacy & Optometry, School of Health Sciences, Faculty of Biology, Medicine & Health, University of Manchester, Manchester, M13 9PT, UK.
[b]Department of Pharmacology and Toxicology, Faculty of Pharmacy, Beni-Suef University, Beni-Suef 62514, Egypt.
[c] Department of Medicinal Chemistry, Faculty of Pharmacy, Zagazig University, Zagazig, Egypt.
[d] BIOtech Center for Biomedical Technologies, Department of Industrial Engineering, University of Trento, Via delle Regole 101, Trento 38123, Italy.

Table of Contents

Figure S2. ^{13}C NMR (125 MHz, DMSO-d_6) spectrum of 2 (*meta*-OMe). 3
Figure S3. ^{19}F NMR (376 MHz, DMSO-d_6) spectrum of 2 (*meta*-OMe). 4
Figure S4. ^{1}H NMR (300 MHz, DMSO-d_6) spectrum of 3 (*para*-OMe). 4
Figure S5. ^{13}C NMR (125 MHz, DMSO-d_6) spectrum of 3 (*para*-OMe). 5
Figure S6. ^{19}F NMR (376 MHz, DMSO-d_6) spectrum of 3 (*para*-OMe). 5
Figure S7. ^{1}H NMR (300 MHz, DMSO-d_6) spectrum of 4 (*meta*-F). 6
Figure S8. ^{13}C NMR (125 MHz, DMSO-d_6) spectrum of 4 (*meta*-F). 6
Figure S9.^{19}F NMR (376 MHz, DMSO-d_6) spectrum of 4 (*meta*-F). 7
Figure S10. ^{1}H NMR (300 MHz, DMSO-d6) spectrum of 5 (*para*-F). 7
Figure S11. ^{13}C NMR (125 MHz, DMSO-d_6) spectrum of 5 (*para*-F). 8
Figure S12. ^{19}F NMR (376 MHz, DMSO-d_6) spectrum of 5 (*para*-F). 8
Figure S13. ^{1}H NMR (300 MHz, DMSO-d_6) spectrum of 6 (*meta*-OMe). 9
Figure S14. ^{13}C NMR (125 MHz, DMSO-d_6) spectrum of 6 (*meta*-OMe). 9
Figure S15. ^{19}F NMR (376 MHz, DMSO-d_6) spectrum of 6 (*meta*-OMe). 10
Figure S16. ^{1}H NMR (300 MHz, DMSO-d_6) spectrum of 7 (*para*-OMe). 10
Figure S17. ^{13}C NMR (125 MHz, DMSO-d_6) spectrum of 7 (*para*-OMe). 11
Figure S18. ^{19}F NMR (376 MHz, DMSO-d_6) spectrum of 7 (*para*-OMe). 11
Figure S19. ^{1}H NMR (300 MHz, DMSO-d_6) spectrum of 8 (*meta*-F). 12

Figure S20. ^{13}C NMR (1mm25 MHz, DMSO-d_6) spectrum of 8 (*meta*-F). 12
Figure S21. ^{19}F NMR (376 MHz, DMSO-d_6) spectrum of 8 (*meta*-F) 13
Figure S22. Dose-response curves for 3-arylisoquinolinones in a panel of six cancer cell lines. 14
Figure S23. NCI One-Dose Mean graphs for 3-arylisoquinolinones 4, 5 and 7........ 15
Figure S24. NCI Five-Dose response curves and mean graphs for 3-arylisoquinolinone 4 (NSC number 795055).. 18
Figure S25. Superposition of top-scoring computationally docked (cyan) and crystallographic pose (grey) of colchicine in tubulin: (a) overview and (b) detail. 22
Figure S26. Gating of cells for cell cycle analysis by flow cytometry. 23
Figure S27. Gating and quadrant setting for Annexin-V/PI apoptosis assay. 23
Table S1. The COMPARE analysis results for 4 against the Standard NCI Database (Top 5 results). 24
Table S2. Docking scores for compounds to tubulin using the FRED docking software with the Chemgauss4 scoring function. 24
Table S3. Average distance dXO and standard deviation (in Å) between ligand atom X=F,Cl and tubulin backbone O atom of Val238. 24
Table S4. Final structure from MD simulation of ligand-tubulin complexes in explicit aqueous solvent. Surface of site shown in grey. 25
Table S5. HPLC-MS purity analysis of compounds 2, 3, 4, 5, 6 and 7 26
Table S6. Molecular Formula Strings of compounds 2-8... 26
References 27

Figure S1. ^{1}H NMR (300 MHz, DMSO-d$_6$) spectrum of **2** (***meta*-OMe**)

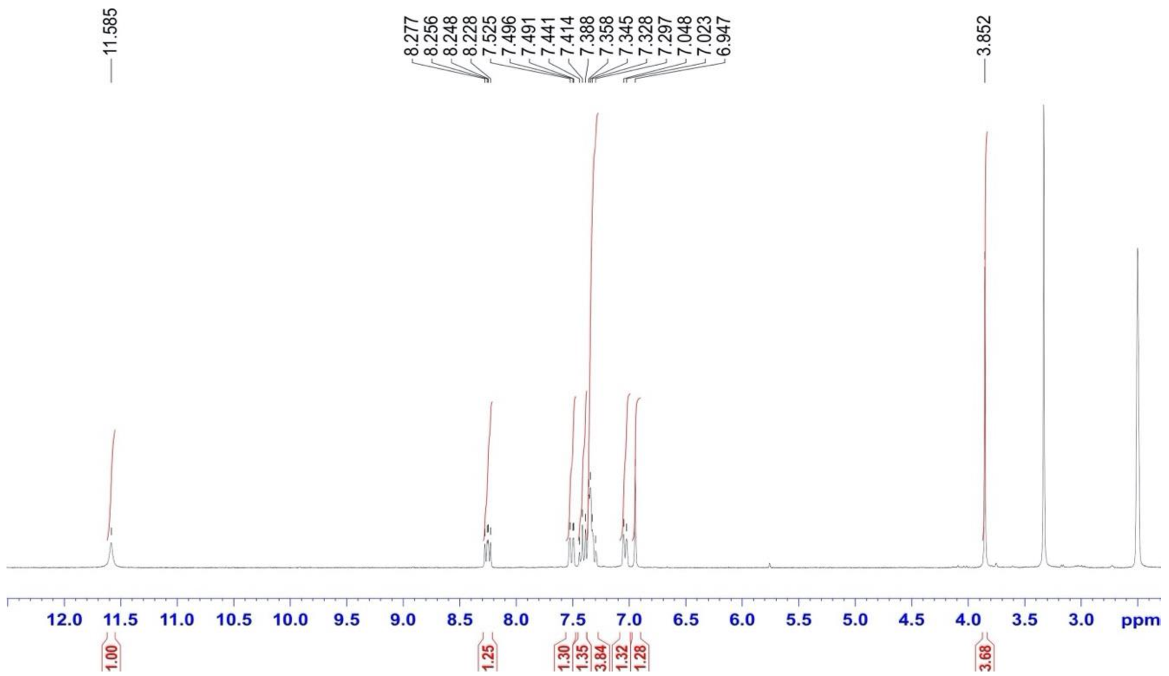

Figure S2. ^{13}C NMR (125 MHz, DMSO-d$_6$) spectrum of **2** (*meta*-OMe).

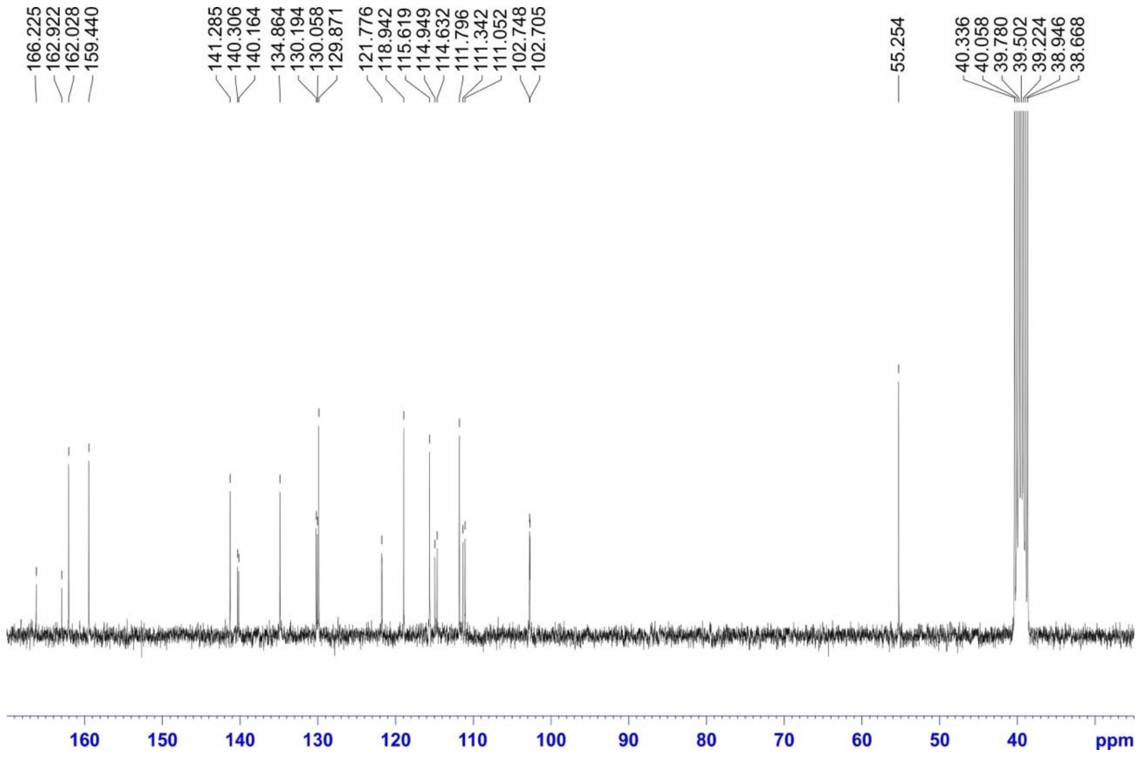

Figure S3. ^{19}F NMR (376 MHz, DMSO-d_6) spectrum of **2** (*meta*-OMe).

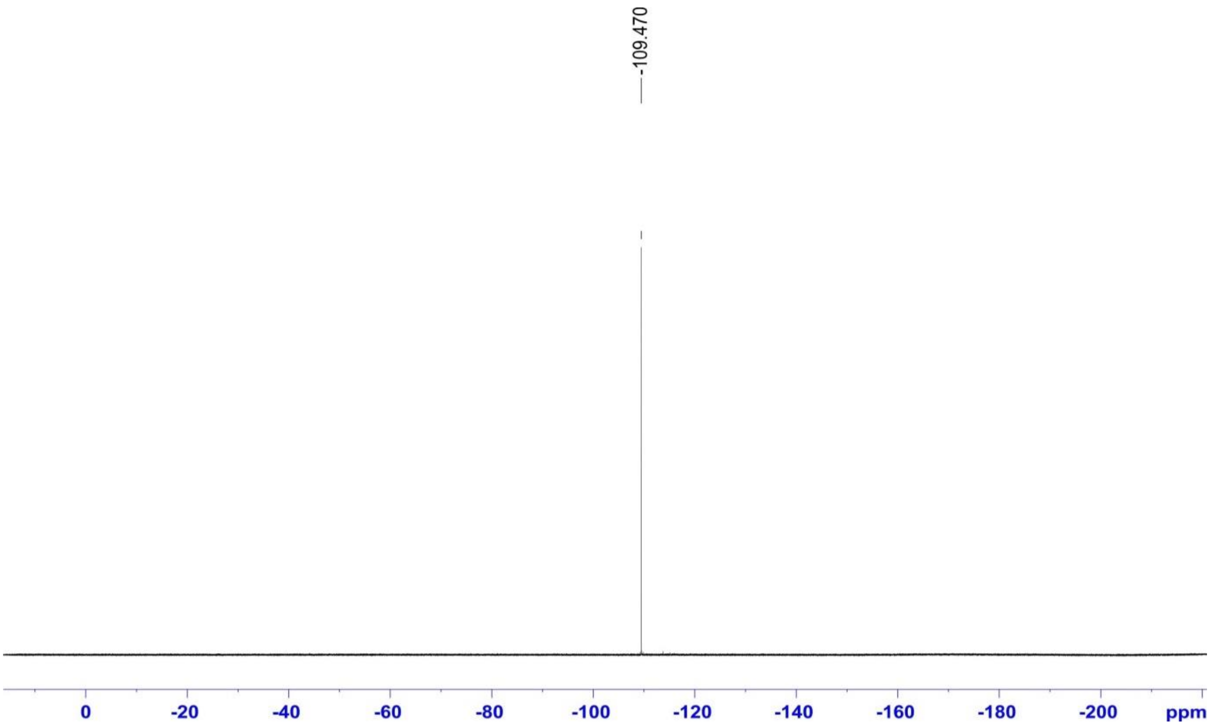

Figure S4. ^{1}H NMR (300 MHz, DMSO-d_6) spectrum of **3** (*para*-OMe).

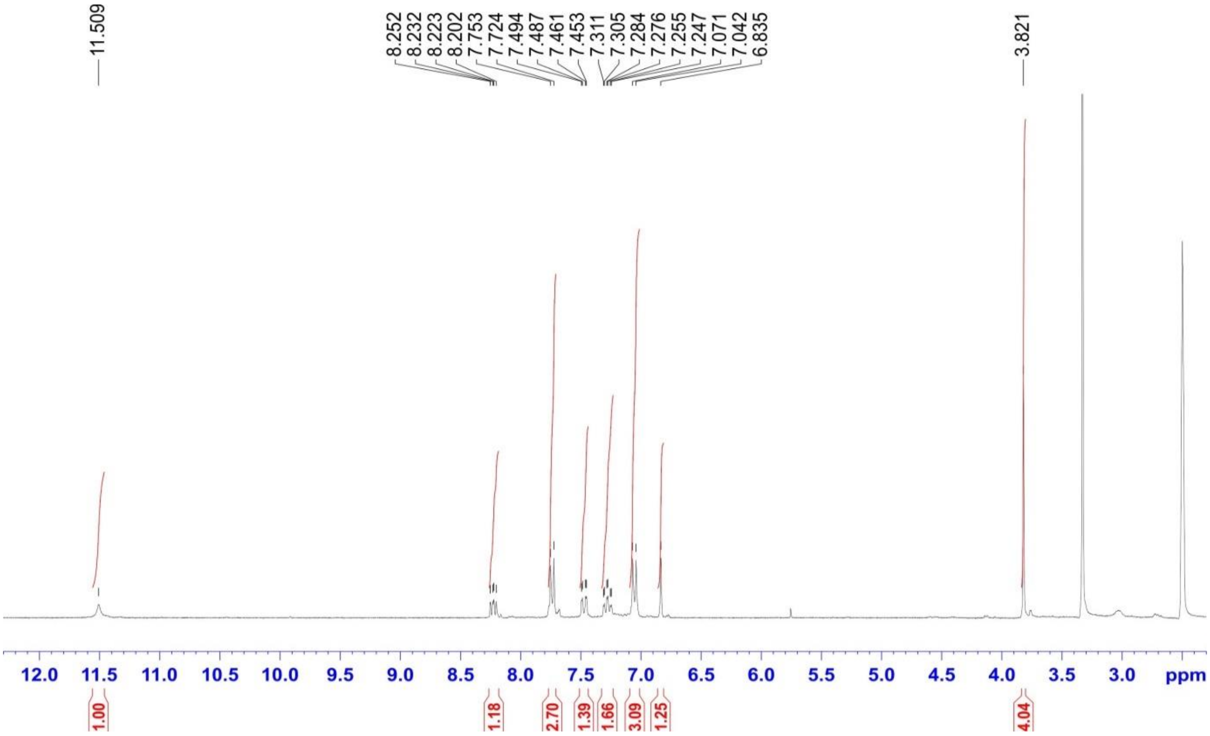

Figure S5. ^{13}C NMR (125 MHz, DMSO-d_6) spectrum of **3** (*para*-OMe).

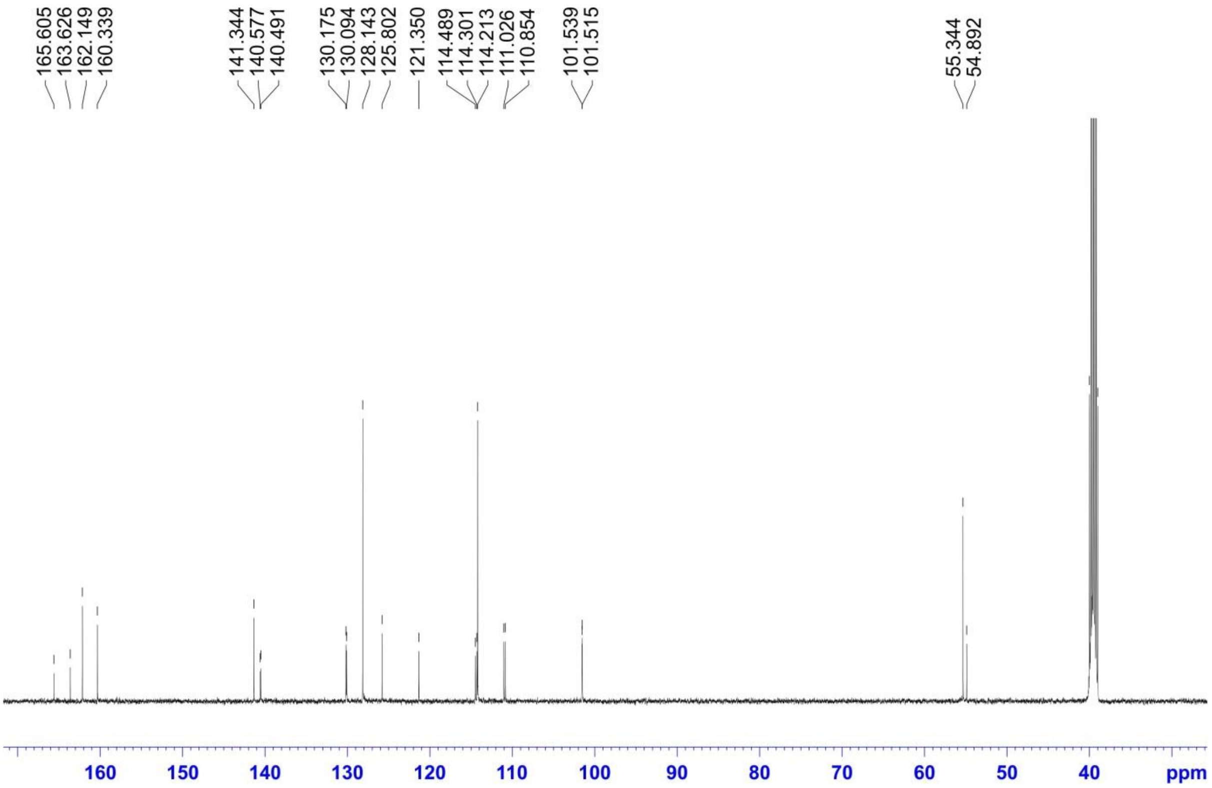

Figure S6. ^{19}F NMR (376 MHz, DMSO-d_6) spectrum of **3** (*para*-OMe).

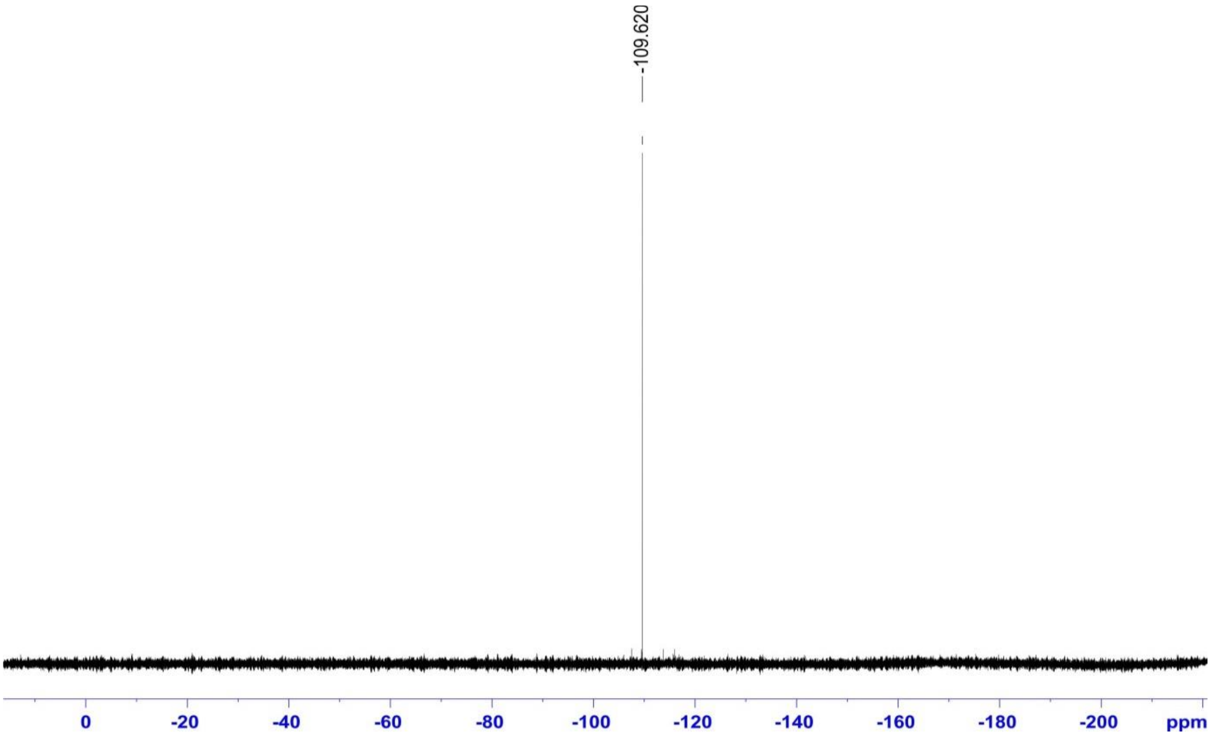

Figure S7. ^{1}H NMR (300 MHz, DMSO-d_6) spectrum of **4** (*meta*-F).

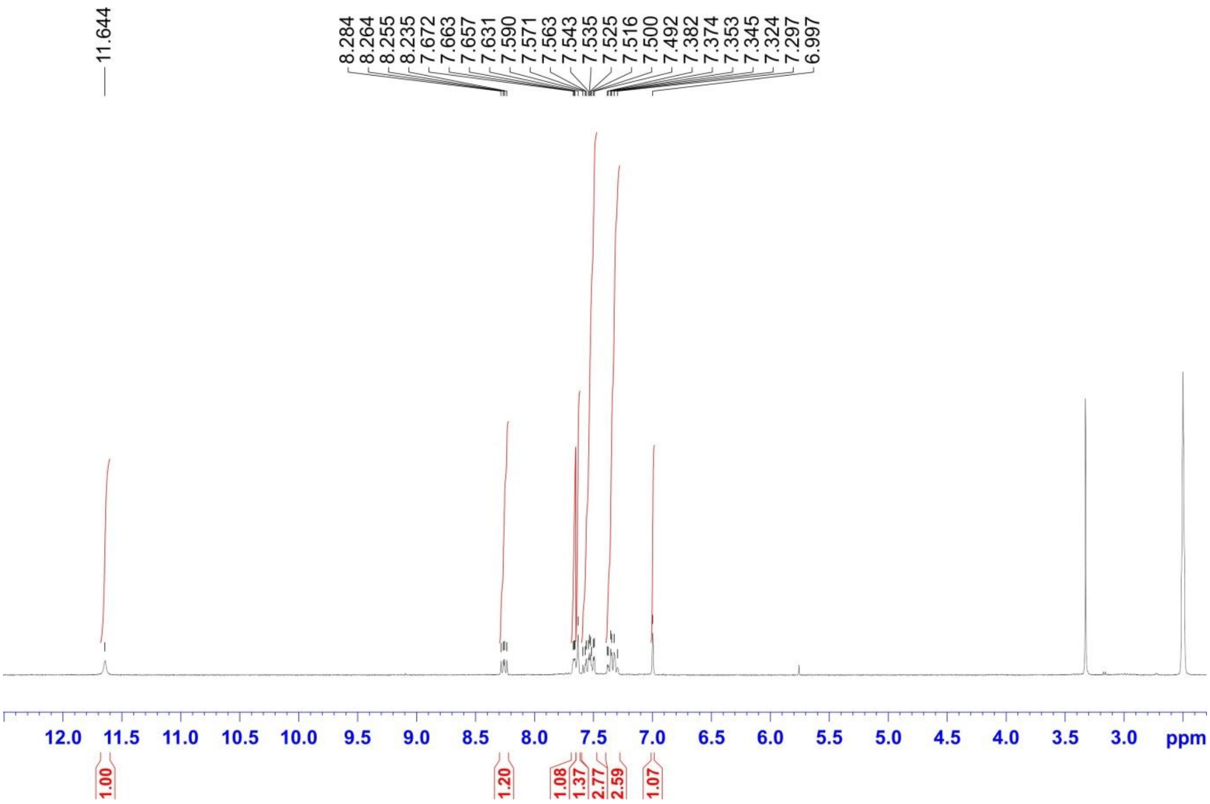

Figure S8. ^{13}C NMR (125 MHz, DMSO-d_6) spectrum of **4** (*meta*-F).

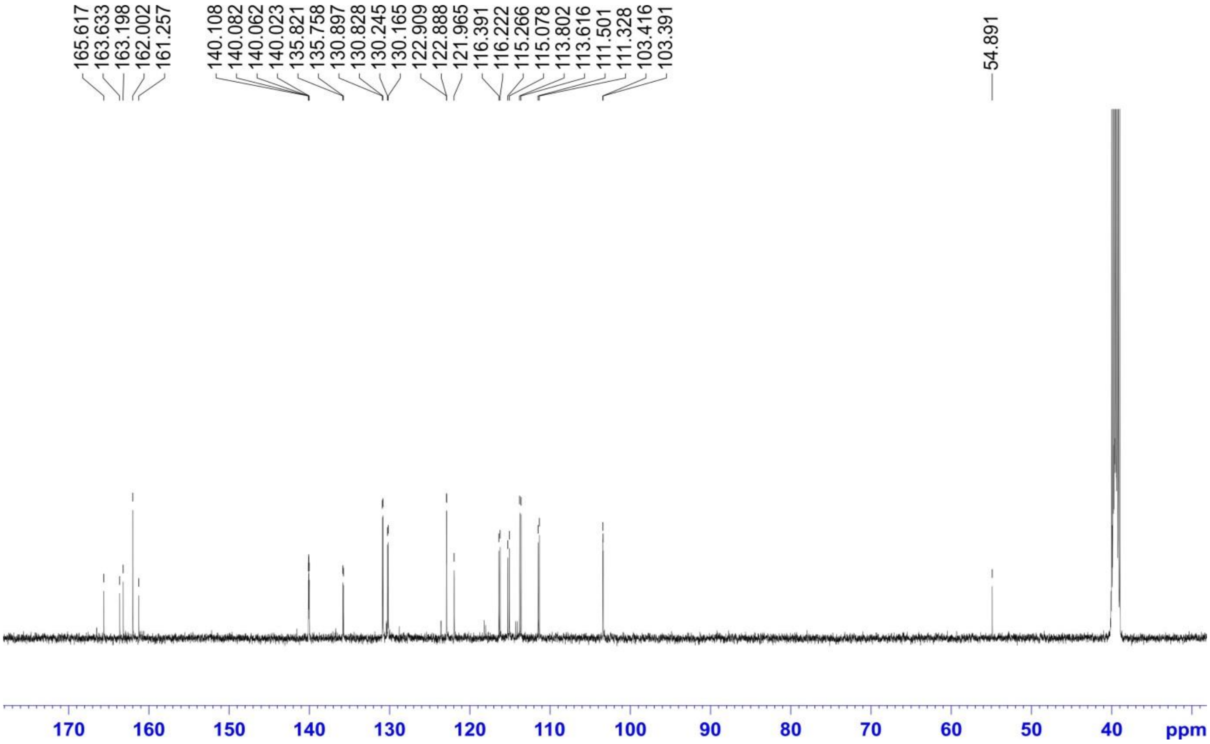

Figure S9.^{19}F NMR (376 MHz, DMSO-d6) spectrum of **4** (*meta*-F).

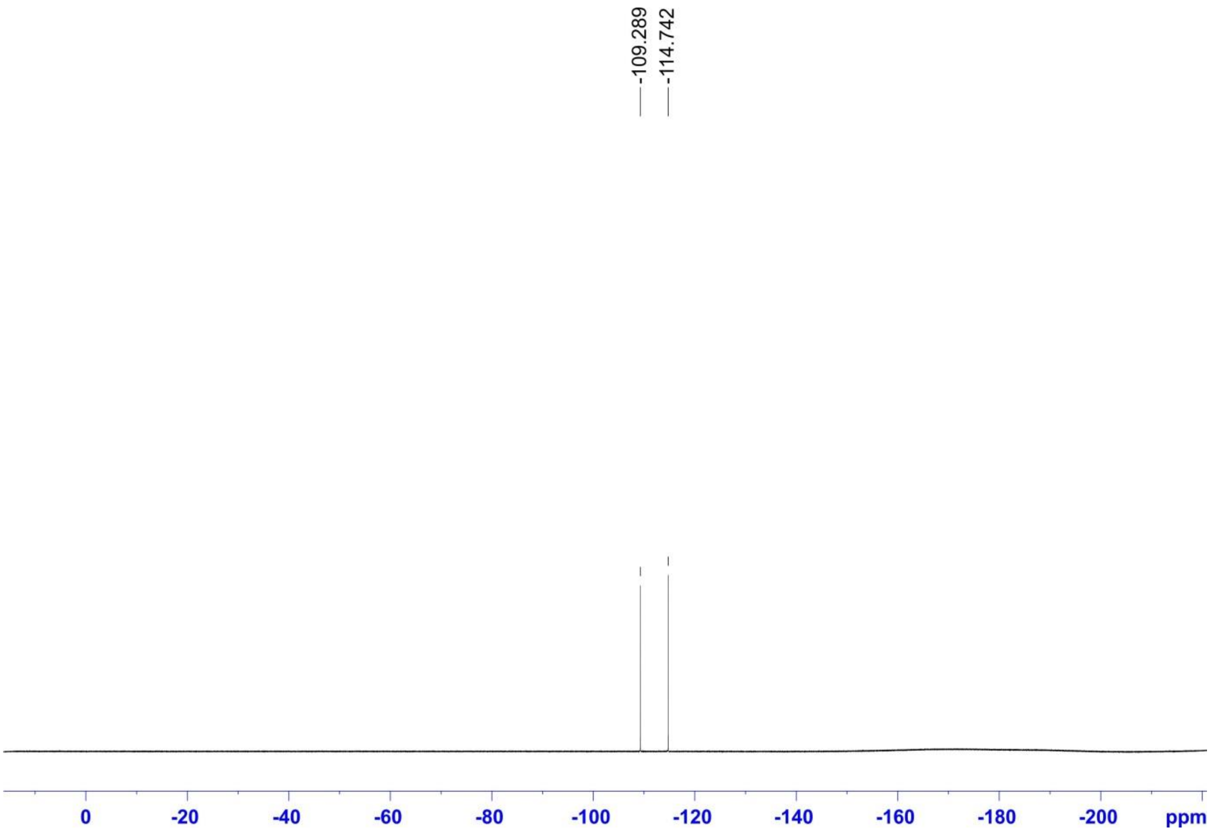

Figure S10. ^{1}H NMR (300 MHz, DMSO-d6) spectrum of **5** (*para*-F).

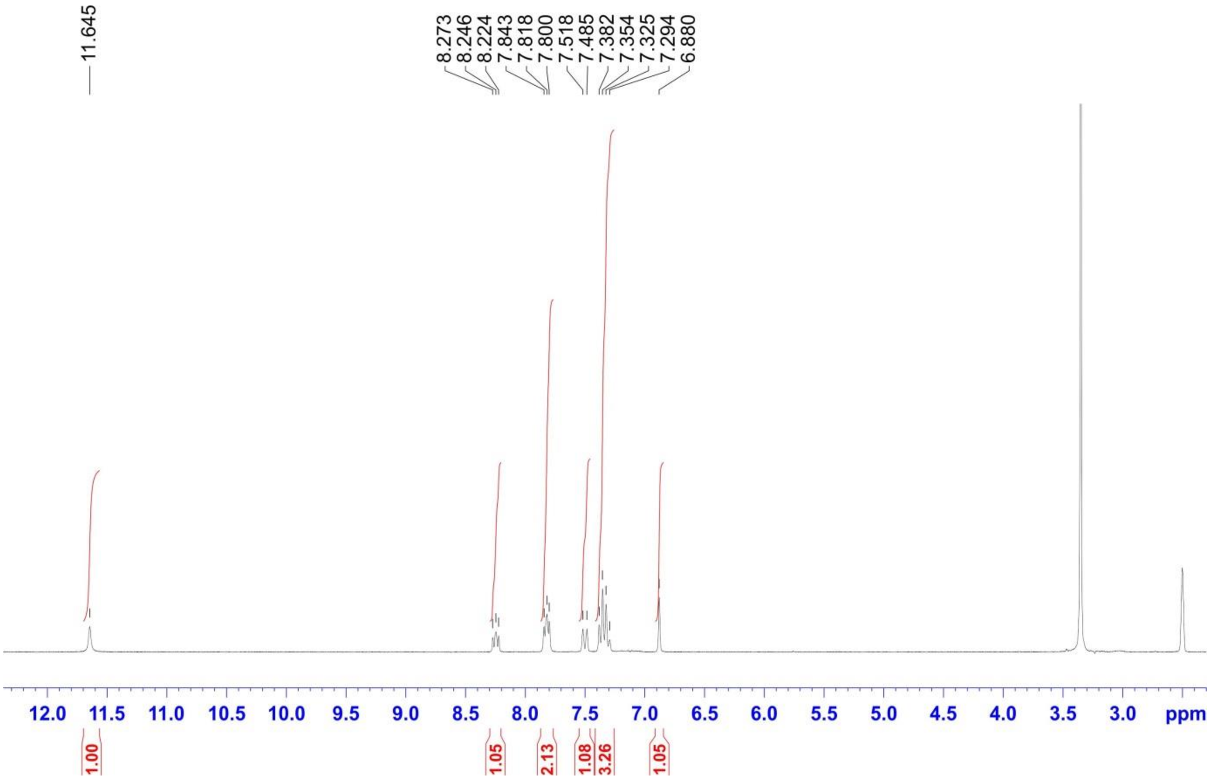

Figure S11. ^{13}C NMR (125 MHz, DMSO-d$_6$) spectrum of **5** (*para*-F).

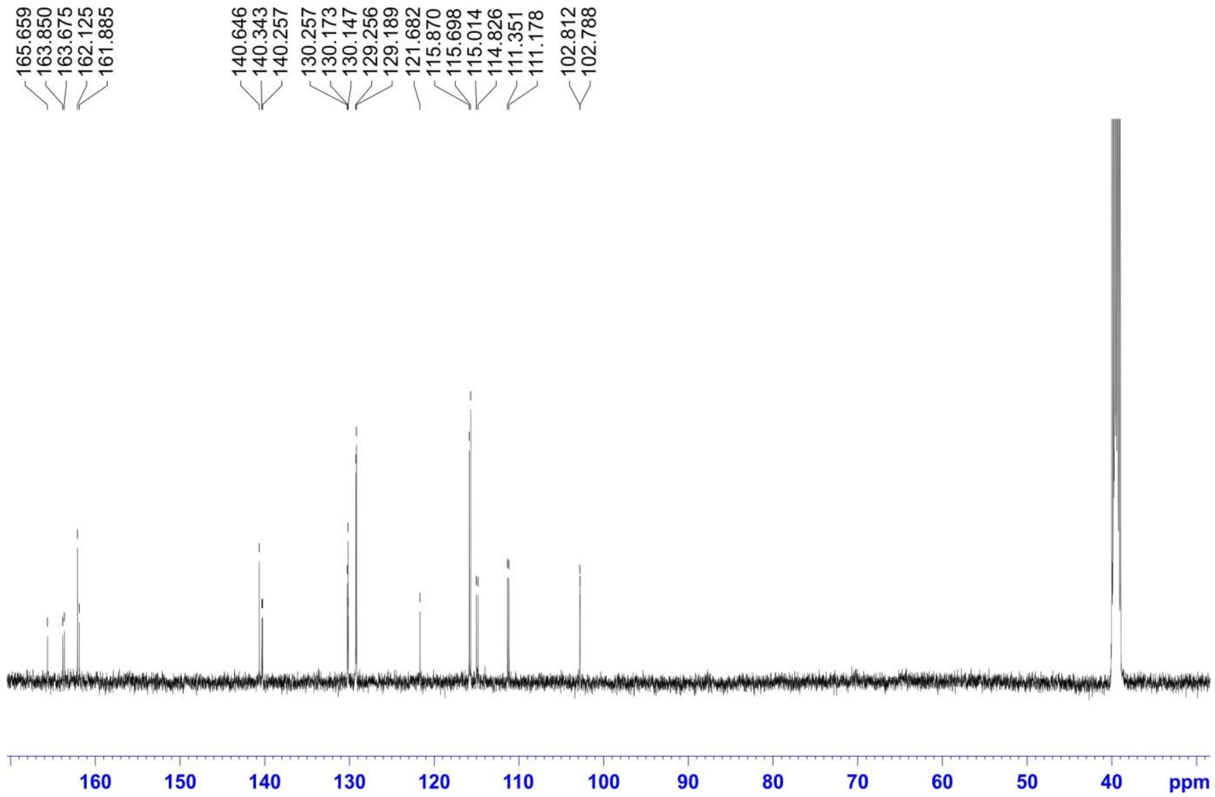

Figure S12. ^{19}F NMR (376 MHz, DMSO-d$_6$) spectrum of **5** (*para*-F).

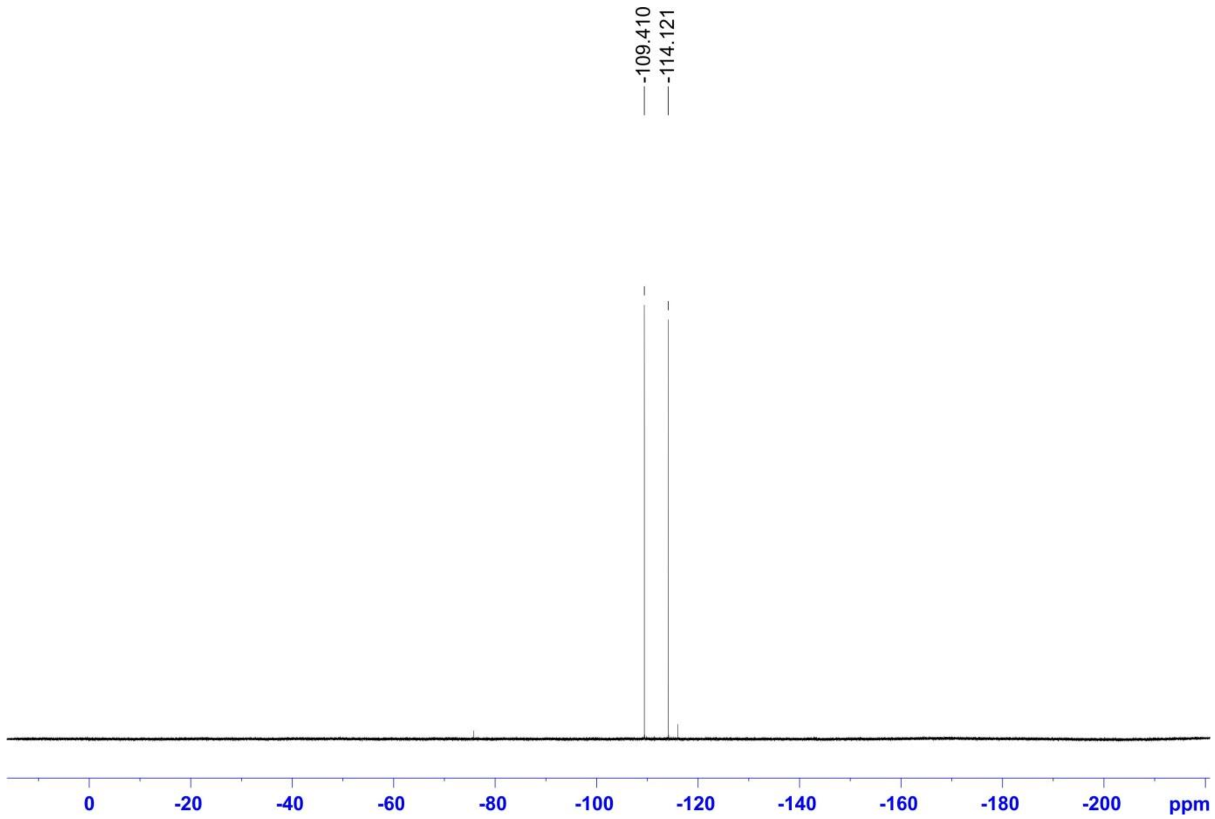

Figure S13. ^{1}H NMR (300 MHz, DMSO-d$_6$) spectrum of **6** (*meta*-OMe).

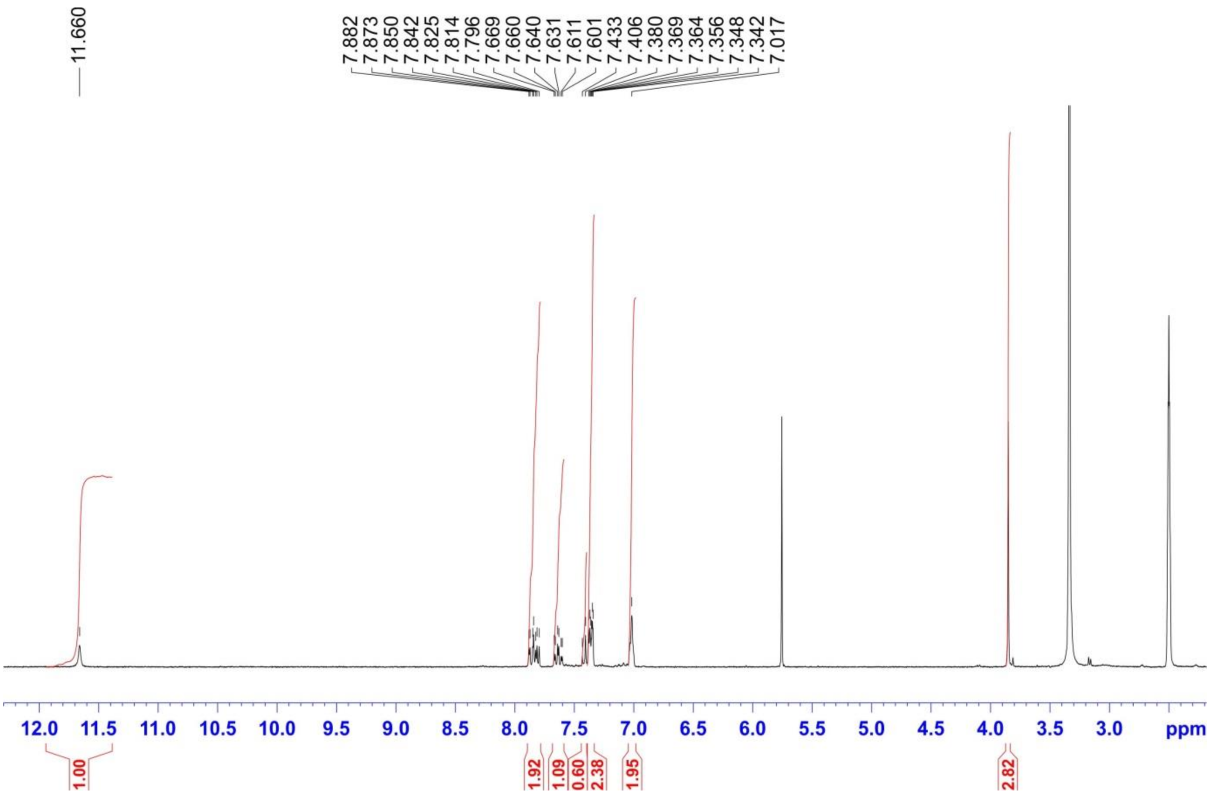

Figure S14. ^{13}C NMR (125 MHz, DMSO-d$_6$) spectrum of **6** (*meta*-OMe).

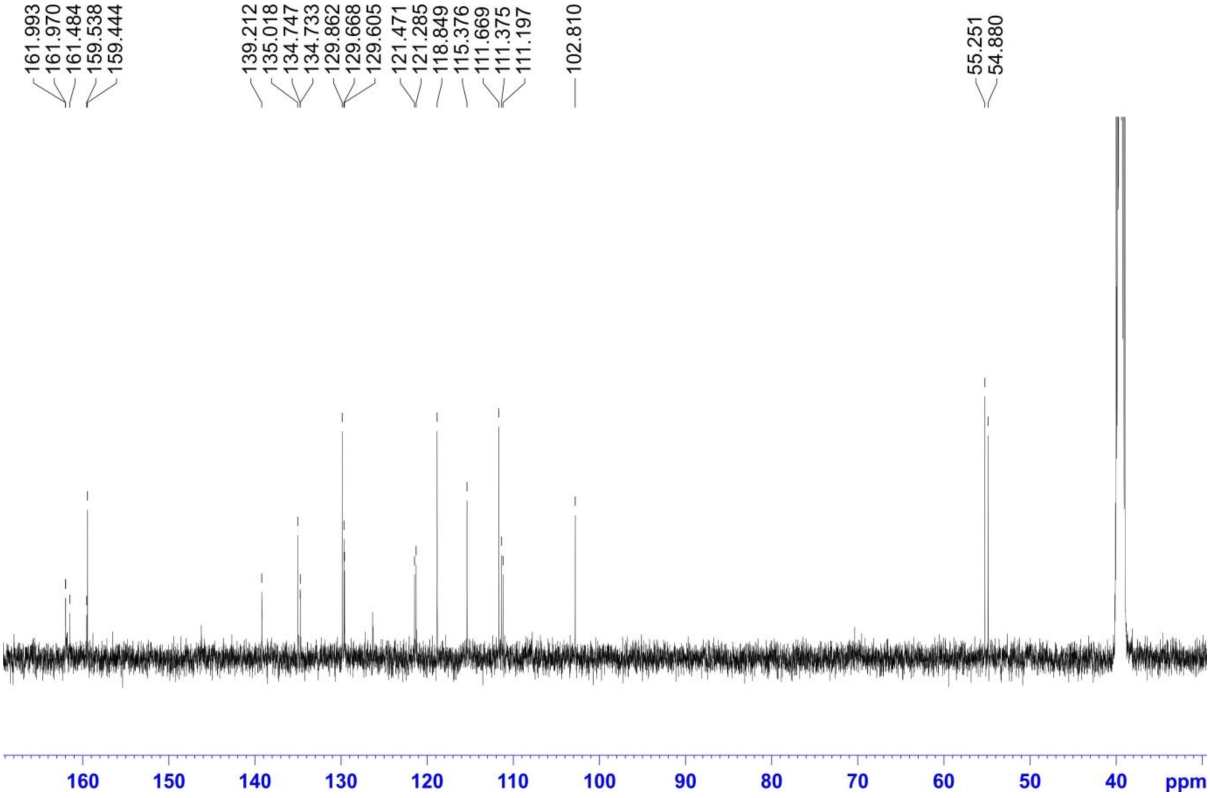

Figure S15. ^{19}F NMR (376 MHz, DMSO-d$_6$) spectrum of **6** (*meta*-OMe).

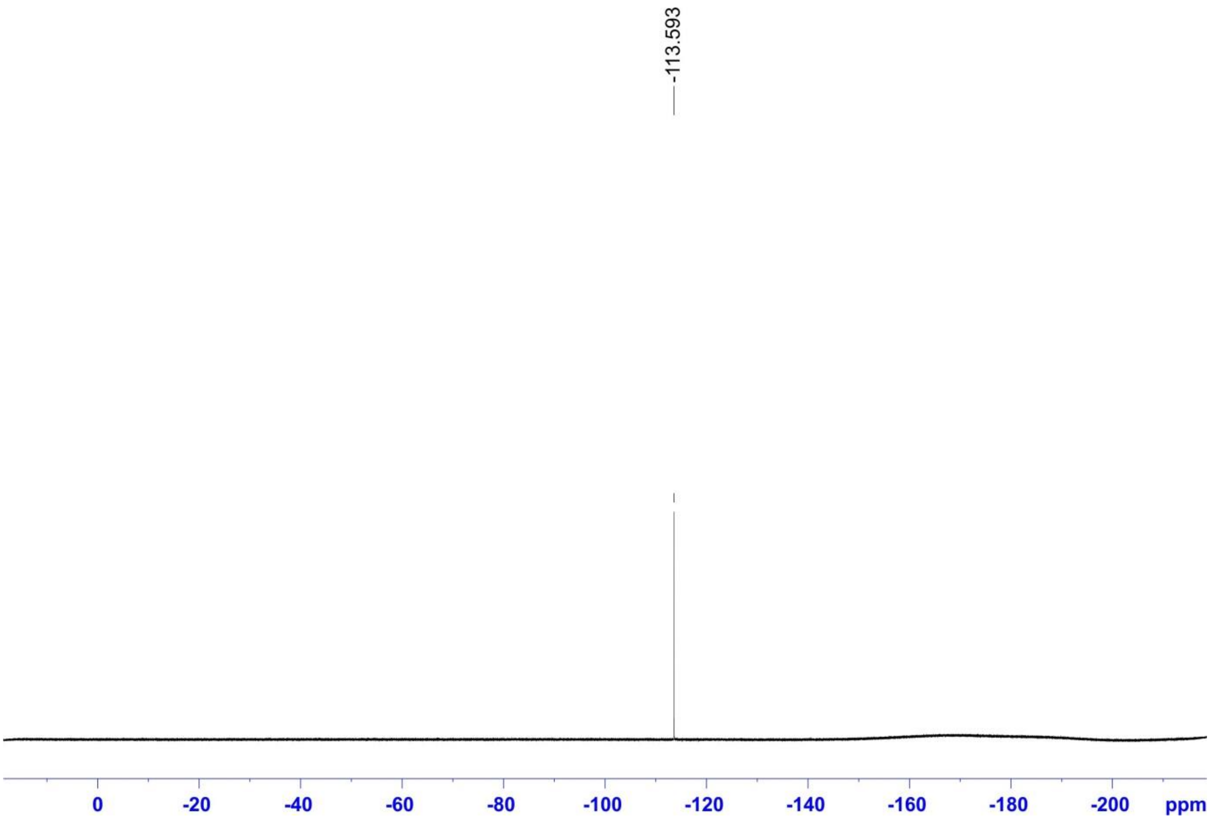

Figure S16. ^{1}H NMR (300 MHz, DMSO-d$_6$) spectrum of **7** (*para*-OMe).

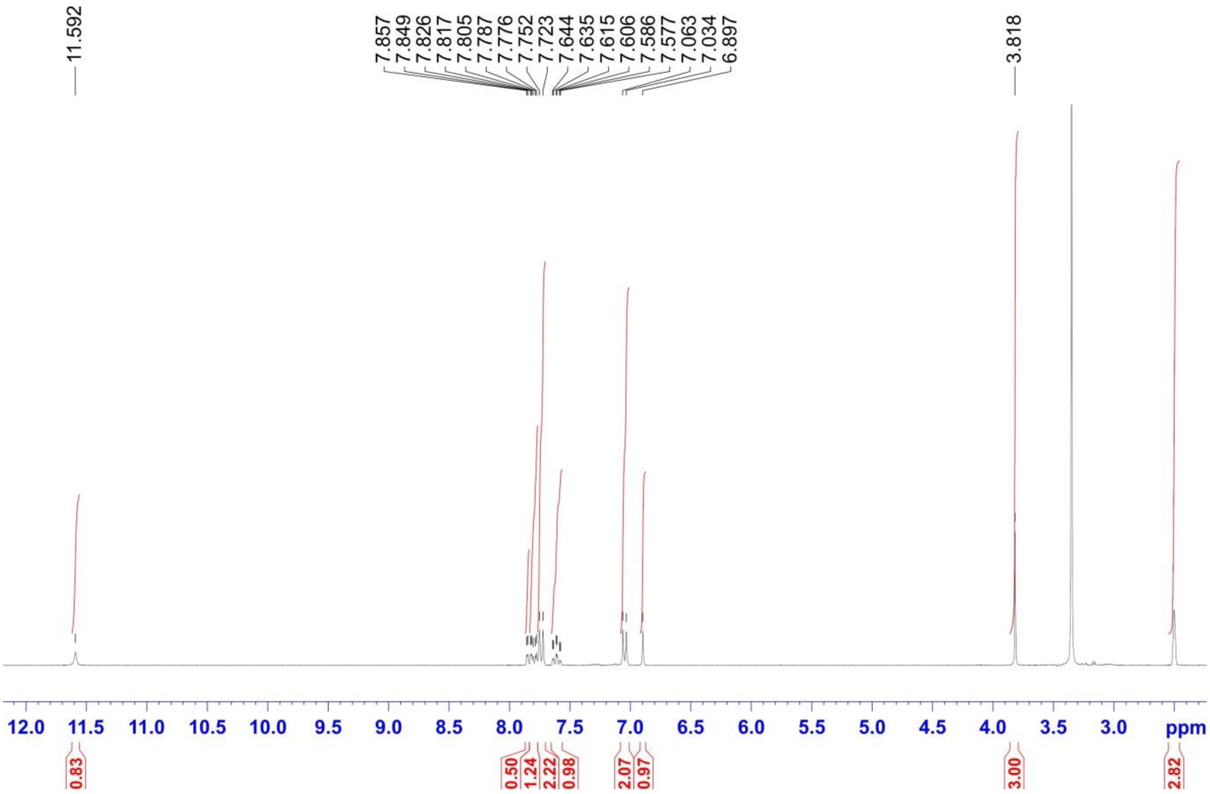

Figure S17. ^{13}C NMR (125 MHz, DMSO-d$_6$) spectrum of **7** (*para*-OMe).

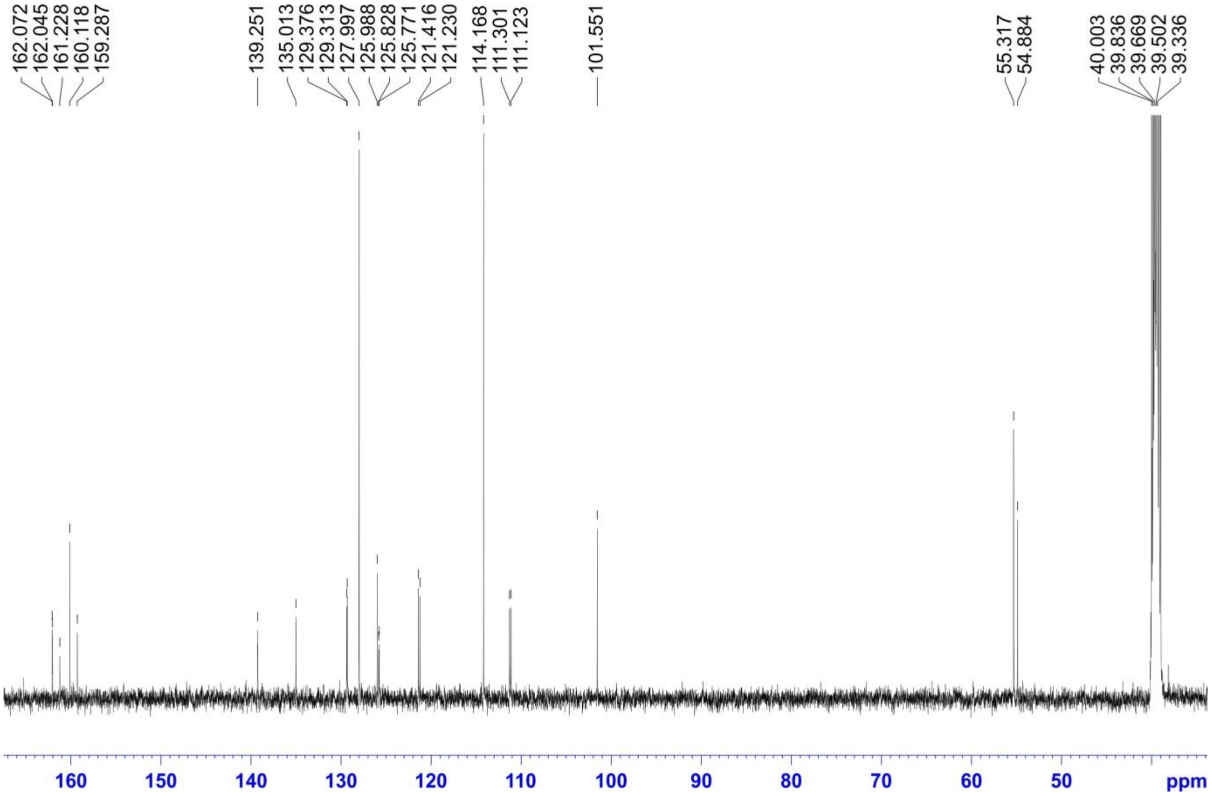

Figure S18. ^{19}F NMR (376 MHz, DMSO-d$_6$) spectrum of **7** (*para*-OMe).

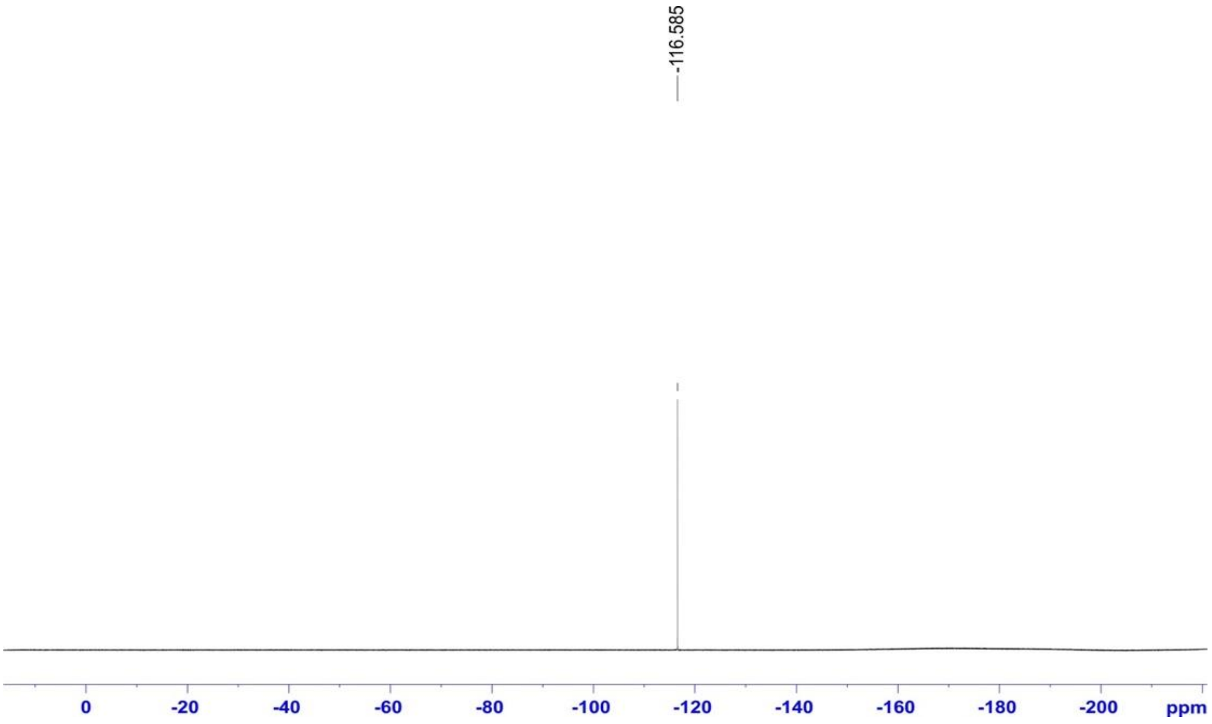

Figure S19. ^{1}H NMR (300 MHz, DMSO-d_6) spectrum of **8** (*meta*-F).

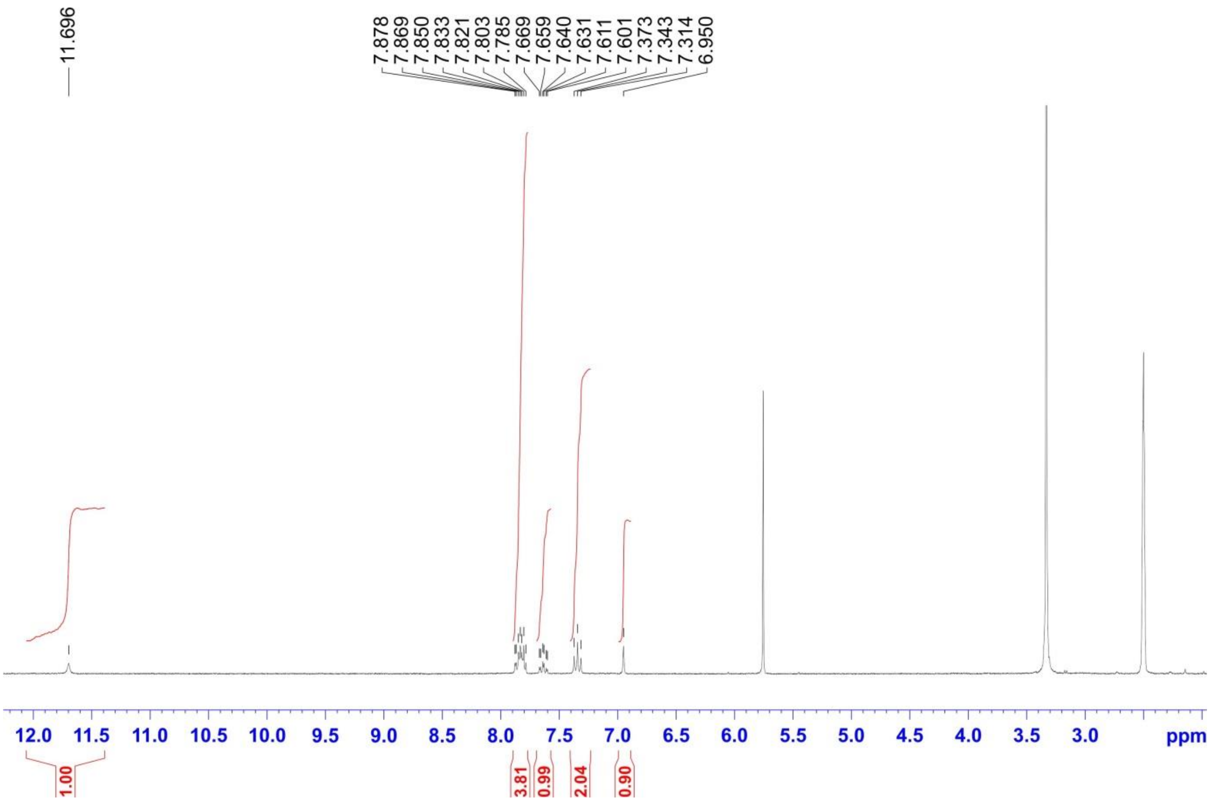

Figure S20. ^{13}C NMR (1mm25 MHz, DMSO-d_6) spectrum of **8** (*meta*-F).

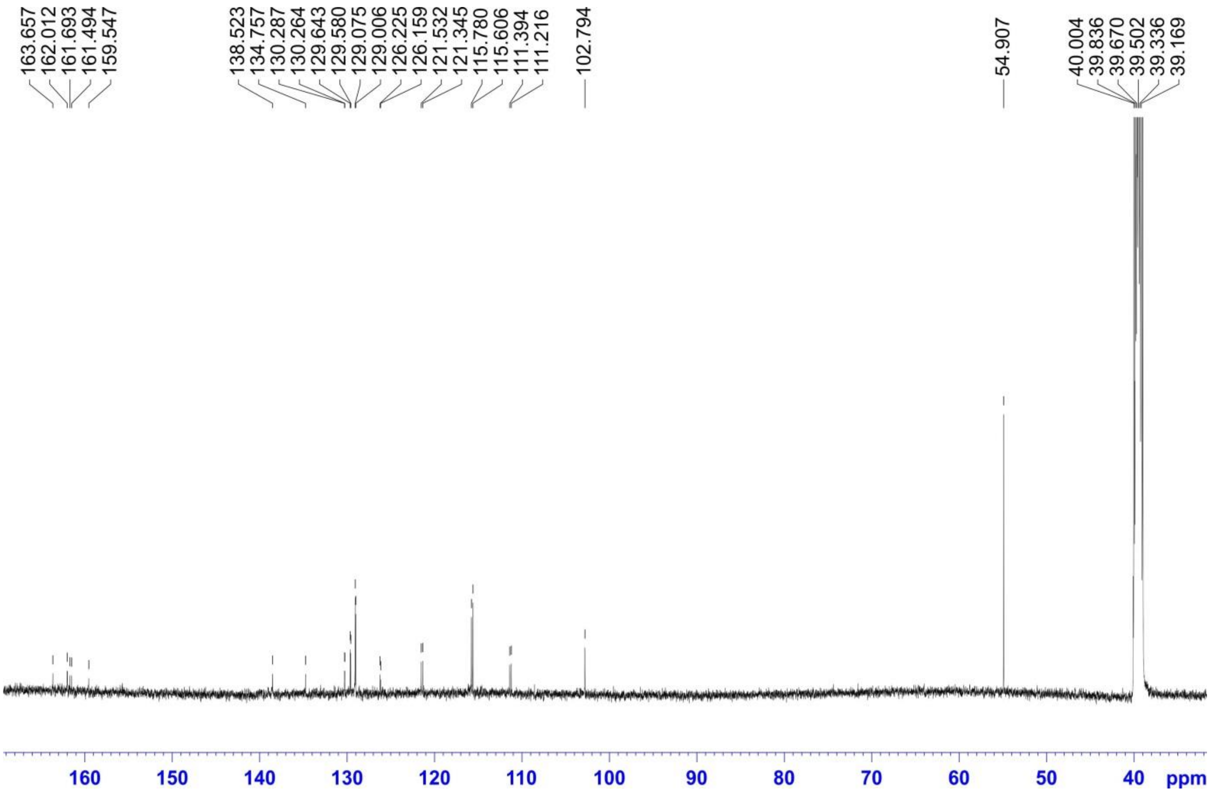

Figure S21. ^{19}F NMR (376 MHz, DMSO-d$_6$) spectrum of **8** (*meta*-F)

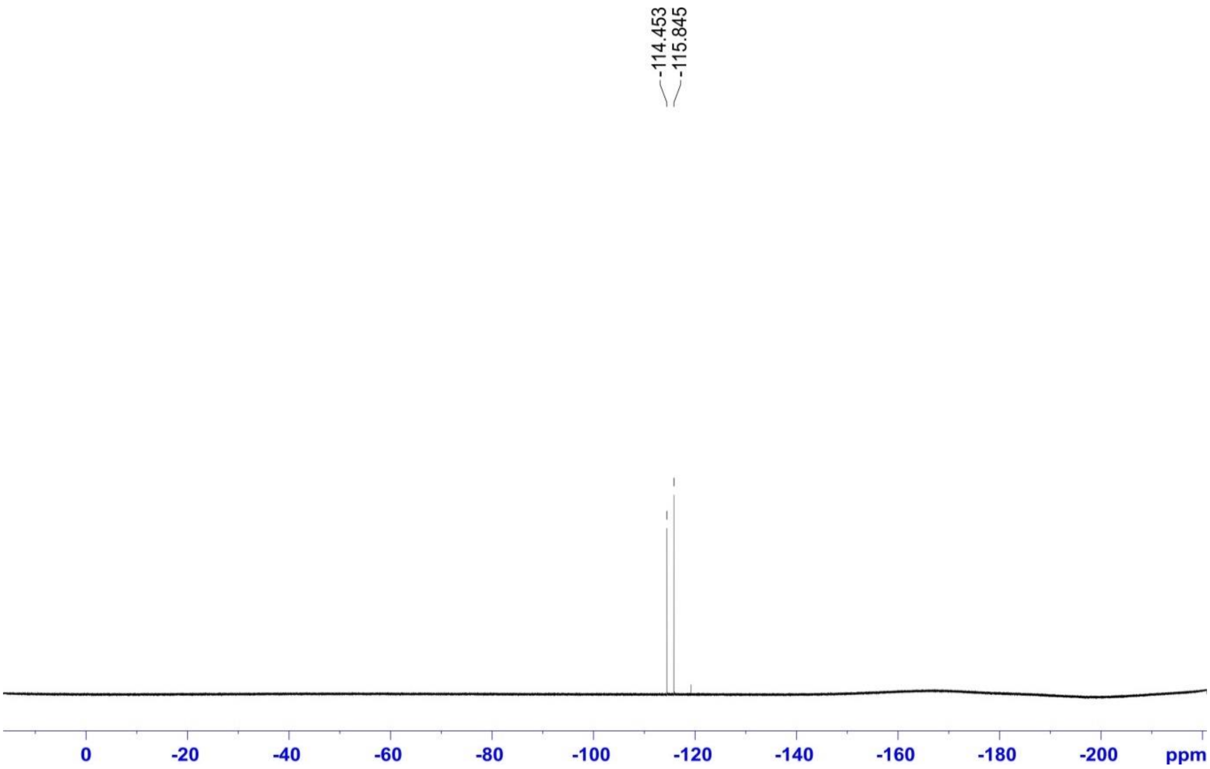

Figure S22. Dose-response curves for 3-arylisoquinolinones in a panel of six cancer cell lines.

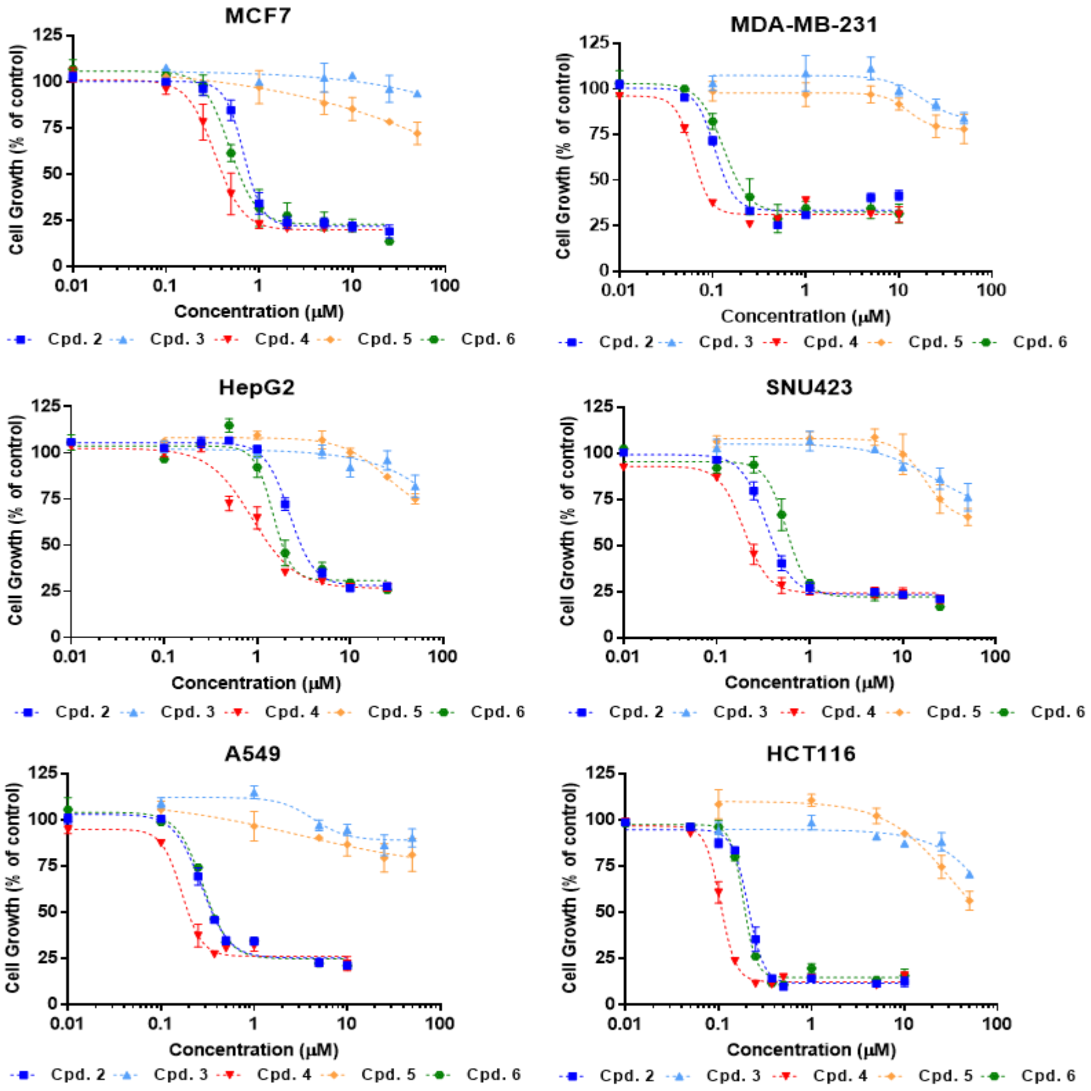

MCF-7, MDA-MB-231, HepG2, SNU423, A549 and HCT116 cells were treated in 96-well plates with DMSO (control) or a range of concentrations of compounds **2–6** for 96 h followed by SRB assay. Non-linear regression dose-response curves depict cell growth as a percentage of that of the control cells using optical density values. Points on each graph represent mean ± SEM from at least three independent experiments.

Figure S23. NCI One-Dose Mean graphs for 3-arylisoquinolinones **4**, **5** and **7**

In NCI one-dose screen, the 59 cell lines (classified into 9 groups) were exposed to **4**, **5** and **7** at a single dose of 10 µM for 48 h followed by SRB assay. The one-dose data were reported as a mean graph of the growth percentage of treated cells, as relative to the cell number of both the no-drug control and the zero time (before adding the test agent). Values between 0 and 100 indicate growth inhibition, whereas values less than 0 indicate lethality. For instance, a value of 40 means 60% growth inhibition; 0, no growth throughout the experiment; -40, 40% lethality; and -100, all cells are dead.[1]

A) One-dose mean graph for 3-arylisoquinolinone **4** (NSC number 795055)

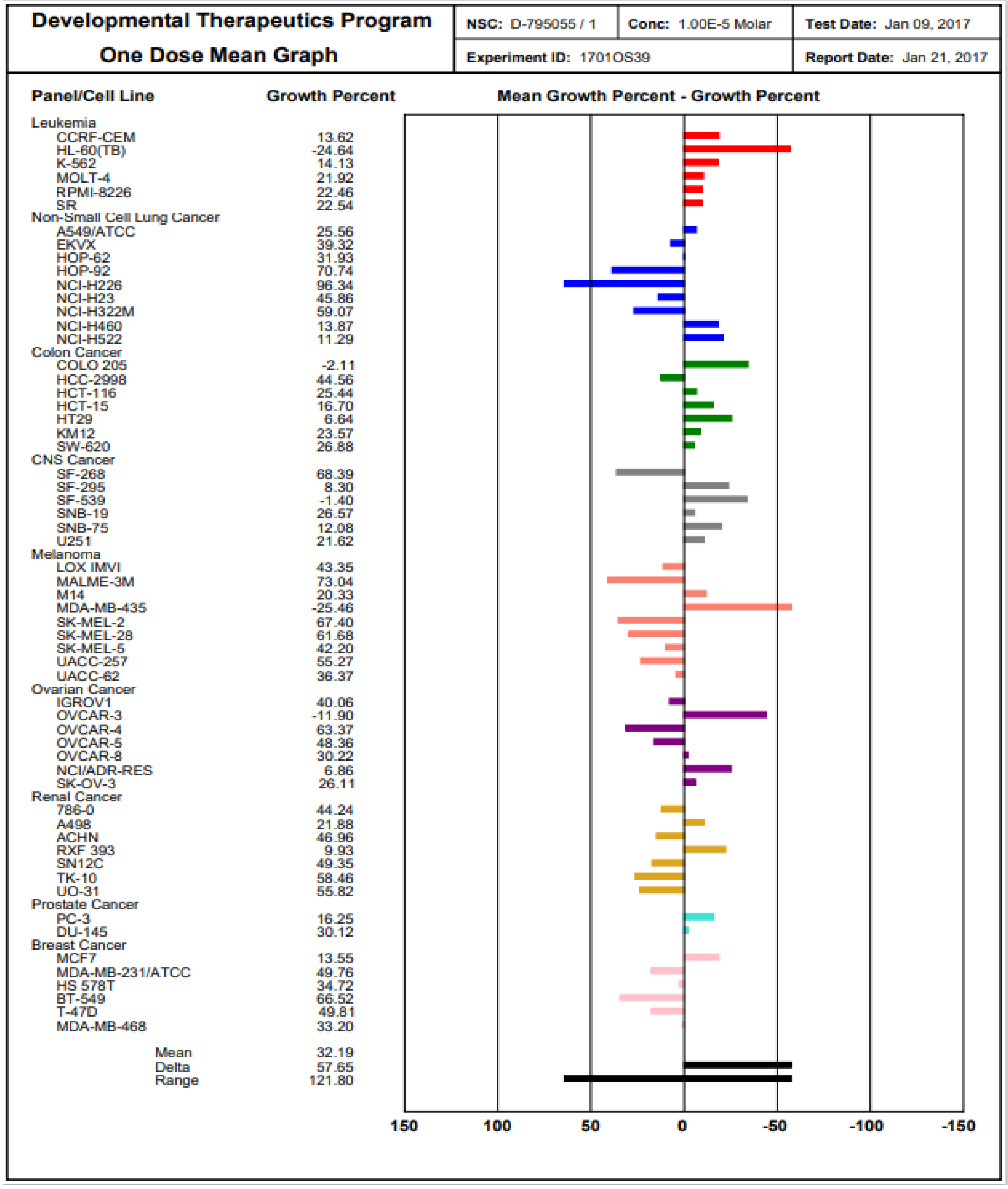

B) One-dose mean graph for 3-arylisoquinolinone **5** (NSC number 795052)

Developmental Therapeutics Program One Dose Mean Graph	NSC: D-795052 / 1	Conc: 1.00E-5 Molar	Test Date: Jan 09, 2017
	Experiment ID: 1701OS39		Report Date: Jan 21, 2017

Panel/Cell Line	Growth Percent
Leukemia	
CCRF-CEM	91.50
HL-60(TB)	68.85
K-562	72.88
MOLT-4	85.08
RPMI-8226	95.86
SR	79.74
Non-Small Cell Lung Cancer	
A549/ATCC	55.76
EKVX	98.02
HOP-62	76.45
HOP-92	93.36
NCI-H226	76.87
NCI-H23	92.42
NCI-H322M	91.84
NCI-H460	54.01
NCI-H522	72.97
Colon Cancer	
COLO 205	90.71
HCC-2998	111.30
HCT-116	58.99
HCT-15	89.65
HT29	75.75
KM12	83.24
SW-620	96.45
CNS Cancer	
SF-268	86.48
SF-295	85.43
SF-539	97.25
SNB-19	82.74
SNB-75	93.46
U251	69.92
Melanoma	
LOX IMVI	98.59
MALME-3M	83.36
M14	98.19
MDA-MB-435	76.63
SK-MEL-2	86.91
SK-MEL-28	110.02
SK-MEL-5	90.42
UACC-257	72.20
UACC-62	91.62
Ovarian Cancer	
IGROV1	87.78
OVCAR-3	99.94
OVCAR-4	68.15
OVCAR-5	100.78
OVCAR-8	61.42
NCI/ADR-RES	78.66
SK-OV-3	68.69
Renal Cancer	
786-0	54.72
A498	68.96
ACHN	97.12
RXF 393	71.68
SN12C	82.70
TK-10	96.63
UO-31	96.97
Prostate Cancer	
PC-3	86.16
DU-145	87.38
Breast Cancer	
MCF7	76.50
MDA-MB-231/ATCC	85.00
HS 578T	93.36
BT-549	87.39
T-47D	48.86
MDA-MB-468	85.79
Mean	83.38
Delta	34.52
Range	62.44

Mean Growth Percent - Growth Percent

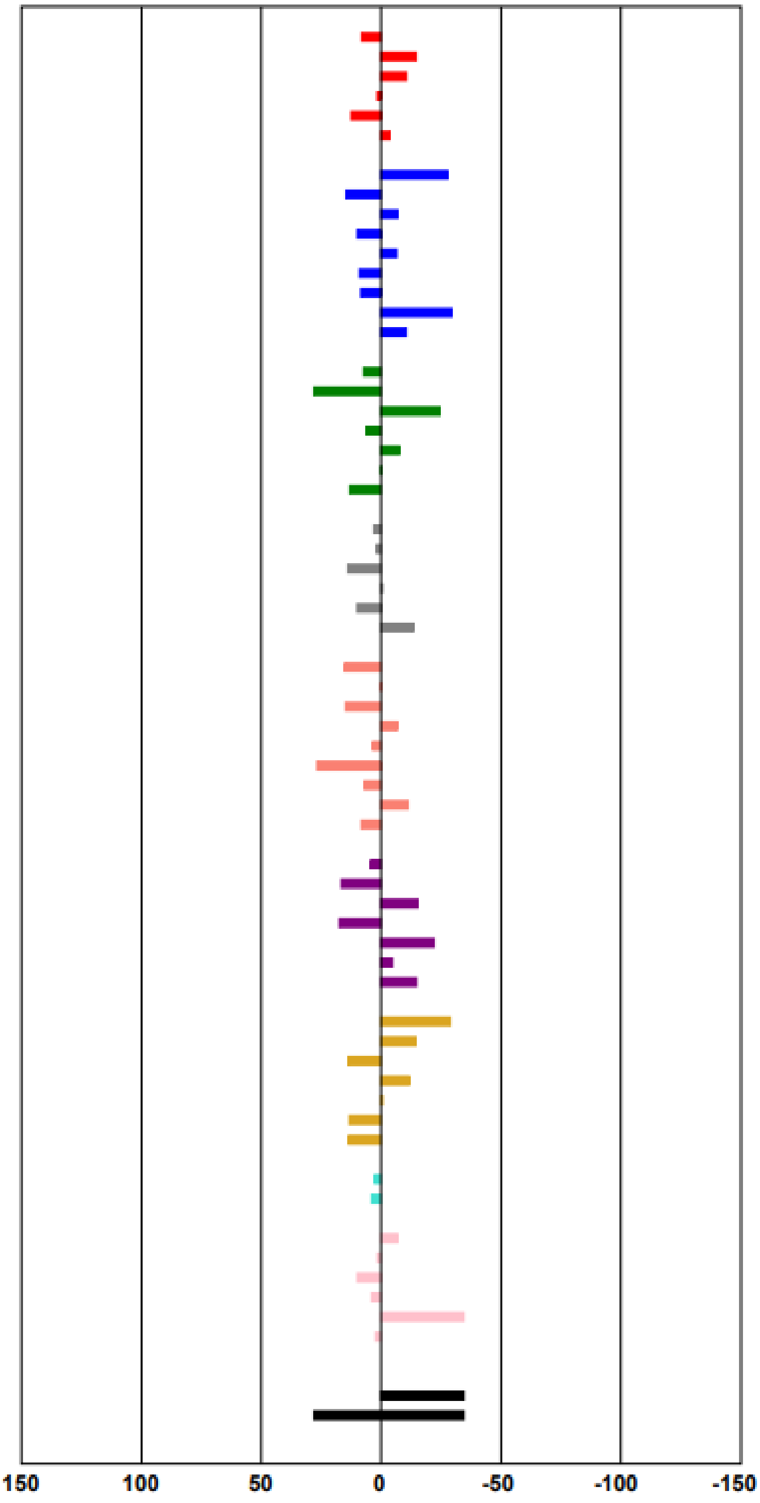

C) One-dose mean graph for 3-arylisoquinolinone **7** (NSC number 795054)

Developmental Therapeutics Program One Dose Mean Graph	**NSC:** D-795054 / 1	**Conc:** 1.00E-5 Molar	**Test Date:** Jan 09, 2017
	**Experiment ID:** 1701OS39		**Report Date:** Jan 21, 2017

Panel/Cell Line	Growth Percent
**Leukemia**	
CCRF-CEM	101.31
HL-60(TB)	87.45
K-562	86.29
MOLT-4	94.89
RPMI-8226	99.07
SR	87.76
**Non-Small Cell Lung Cancer**	
A549/ATCC	38.31
EKVX	82.07
HOP-62	91.23
HOP-92	102.73
NCI-H226	72.84
NCI-H23	80.80
NCI-H322M	71.36
NCI-H460	50.57
NCI-H522	76.67
**Colon Cancer**	
COLO 205	97.13
HCC-2998	98.20
HCT-116	97.09
HCT-15	102.70
HT29	75.64
KM12	99.67
SW-620	102.63
**CNS Cancer**	
SF-268	95.09
SF-295	96.72
SF-539	98.87
SNB-19	91.46
SNB-75	94.30
U251	67.53
**Melanoma**	
LOX IMVI	100.93
MALME-3M	104.04
M14	101.16
MDA-MB-435	108.78
SK-MEL-2	92.62
SK-MEL-28	107.55
SK-MEL-5	98.85
UACC-257	80.60
UACC-62	102.52
**Ovarian Cancer**	
IGROV1	82.05
OVCAR-3	87.39
OVCAR-4	88.01
OVCAR-5	98.16
OVCAR-8	74.59
NCI/ADR-RES	85.64
SK-OV-3	80.43
**Renal Cancer**	
786-0	99.72
A498	41.19
ACHN	107.32
RXF 393	90.95
SN12C	100.87
TK-10	93.39
UO-31	70.31
**Prostate Cancer**	
PC-3	93.09
DU-145	100.15
**Breast Cancer**	
MCF7	42.59
MDA-MB-231/ATCC	96.26
HS 578T	107.06
BT-549	91.91
T-47D	74.52
MDA-MB-468	74.58
Mean	88.43
Delta	50.12
Range	70.47

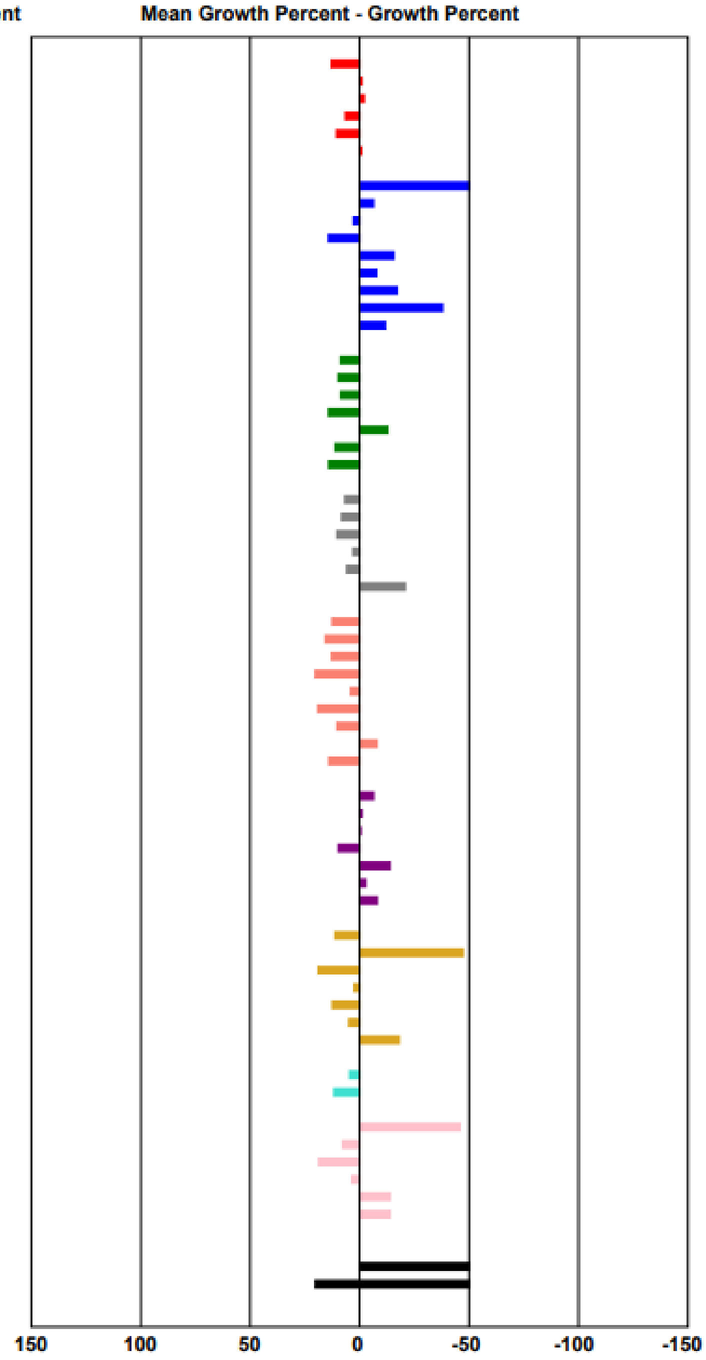

Figure S24. NCI Five-Dose response curves and mean graphs for 3-arylisoquinolinone **4** (NSC number 795055).

The 59 cell lines panel was treated with **4,** that demonstrated marked growth inhibition in the One-Dose Screen, at five concentrations for 48 h followed by SRB assay. In mean graphs, the zero value on the vertical line denotes Log_{10} of the mean values of each of the GI_{50}, TGI and LC_{50}. The graph pattern is plotted as bars from positive and negative values called deltas and calculated by a specific way.[2] The positive value bars on the right of the vertical line are below the mean value and represent cell lines that are more sensitive to **4**. The negative bars on the left of the vertical line exceed the mean and represent cells that are more resistant to **4.**

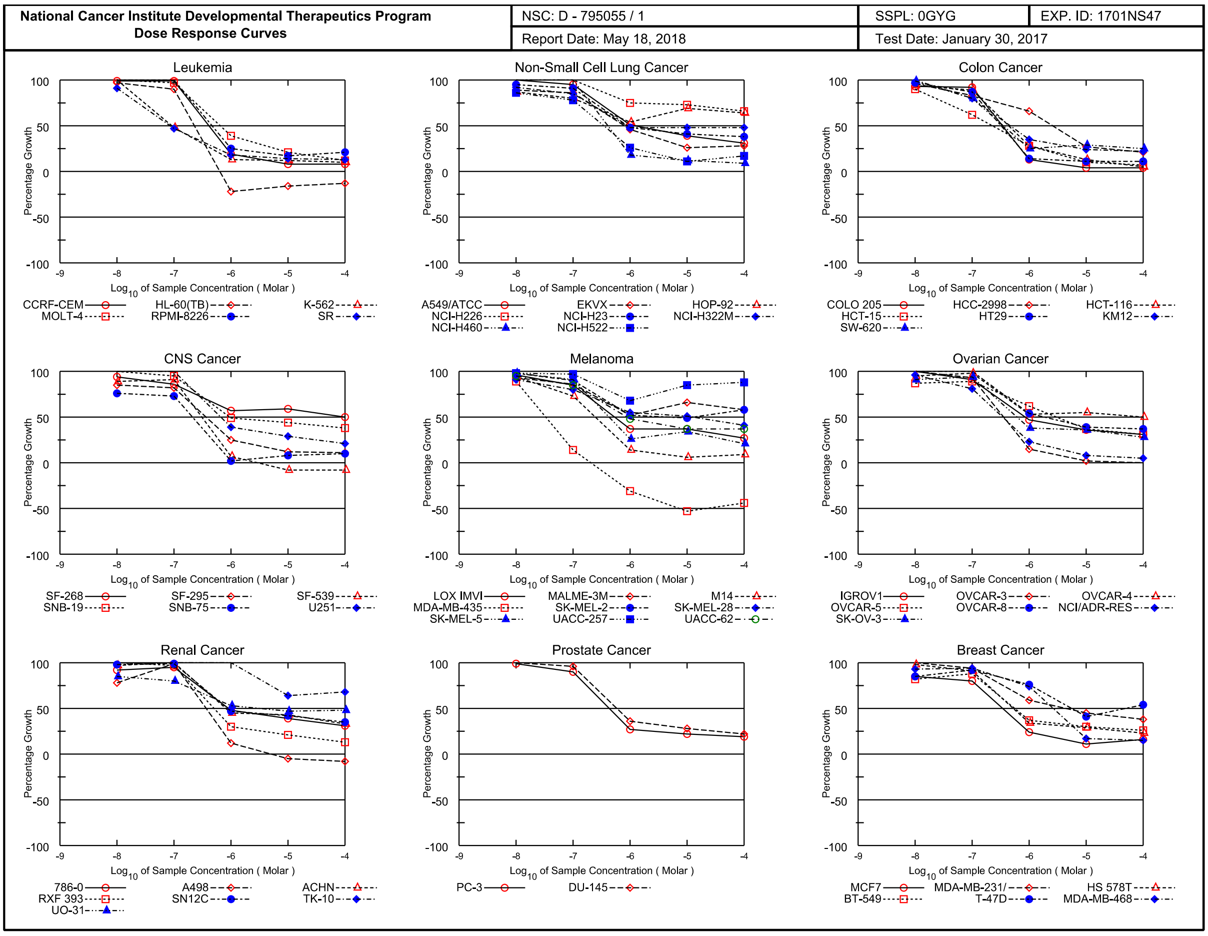

National Cancer Institute Developmental Therapeutics Program
In-Vitro Testing Results

NSC : D - 795055 / 1	Experiment ID : 1701NS47	Test Type : 08	Units : Molar
Report Date : May 18, 2018	Test Date : January 30, 2017	QNS :	MC :
COMI : AB00107-2	Stain Reagent : SRB Dual-Pass Related	SSPL : 0GYG	

			Log10 Concentration												
	Time		Mean Optical Densities					Percent Growth							
Panel/Cell Line	Zero	Ctrl	-8.0	-7.0	-6.0	-5.0	-4.0	-8.0	-7.0	-6.0	-5.0	-4.0	GI50	TGI	LC50
Leukemia															
CCRF-CEM	0.623	3.052	3.039	3.018	1.083	0.806	0.814	99	99	19	8	8	4.07E-7	> 1.00E-4	> 1.00E-4
HL-60(TB)	0.842	3.003	2.935	2.794	0.659	0.711	0.736	97	90	-22	-16	-13	2.29E-7	6.40E-7	> 1.00E-4
K-562	0.226	2.265	2.404	1.202	0.491	0.473	0.424	107	48	13	12	10	9.20E-8	> 1.00E-4	> 1.00E-4
MOLT-4	0.803	3.059	3.119	2.997	1.689	1.276	1.080	103	97	39	21	12	6.52E-7	> 1.00E-4	> 1.00E-4
RPMI-8226	0.767	2.762	2.795	2.778	1.260	1.110	1.193	102	101	25	17	21	4.65E-7	> 1.00E-4	> 1.00E-4
SR	0.367	1.149	1.077	0.733	0.508	0.474	0.466	91	47	18	14	13	8.45E-8	> 1.00E-4	> 1.00E-4
Non-Small Cell Lung Cancer															
A549/ATCC	0.524	2.427	2.437	2.340	1.487	1.266	1.120	101	95	51	39	31	1.12E-6	> 1.00E-4	> 1.00E-4
EKVX	0.747	2.417	2.228	2.187	1.511	1.175	1.208	89	86	46	26	28	7.85E-7	> 1.00E-4	> 1.00E-4
HOP-92	1.233	1.963	1.866	1.816	1.627	1.740	1.700	87	80	54	69	64	> 1.00E-4	> 1.00E-4	> 1.00E-4
NCI-H226	1.055	2.320	2.317	2.365	1.998	1.973	1.885	100	104	75	73	66	> 1.00E-4	> 1.00E-4	> 1.00E-4
NCI-H23	0.661	2.036	1.961	1.908	1.326	1.226	1.181	95	91	48	41	38	9.13E-7	> 1.00E-4	> 1.00E-4
NCI-H322M	1.063	2.431	2.325	2.220	1.717	1.716	1.723	92	85	48	48	48	8.72E-7	> 1.00E-4	> 1.00E-4
NCI-H460	0.337	3.180	3.271	3.214	0.857	0.665	0.604	103	101	18	12	9	4.14E-7	> 1.00E-4	> 1.00E-4
NCI-H522	1.256	2.940	2.701	2.565	1.701	1.448	1.550	86	78	26	11	17	3.47E-7	> 1.00E-4	> 1.00E-4
Colon Cancer															
COLO 205	0.670	2.494	2.364	2.356	0.902	0.746	0.752	93	92	13	4	4	3.41E-7	> 1.00E-4	> 1.00E-4
HCC-2998	0.631	2.321	2.249	2.038	1.743	1.085	0.993	96	83	66	27	21	2.54E-6	> 1.00E-4	> 1.00E-4
HCT-116	0.243	2.276	2.146	2.046	0.806	0.517	0.344	94	89	28	13	5	4.31E-7	> 1.00E-4	> 1.00E-4
HCT-15	0.225	1.651	1.511	1.110	0.621	0.365	0.330	90	62	28	10	7	2.25E-7	> 1.00E-4	> 1.00E-4
HT29	0.362	2.372	2.320	2.115	0.652	0.575	0.588	97	87	14	11	11	3.24E-7	> 1.00E-4	> 1.00E-4
KM12	0.508	2.832	2.870	2.361	1.331	1.063	1.027	102	80	35	24	22	4.69E-7	> 1.00E-4	> 1.00E-4
SW-620	0.331	2.202	2.175	1.835	0.800	0.872	0.792	99	80	25	29	25	3.54E-7	> 1.00E-4	> 1.00E-4
CNS Cancer															
SF-268	0.709	2.362	2.259	2.134	1.655	1.678	1.541	94	86	57	59	50	> 1.00E-4	> 1.00E-4	> 1.00E-4
SF-295	0.631	2.330	2.082	2.021	1.056	0.836	0.824	85	82	25	12	11	3.63E-7	> 1.00E-4	> 1.00E-4
SF-539	0.873	2.546	2.359	2.392	0.996	0.800	0.805	89	91	7	-8	-8	3.08E-7	2.92E-6	> 1.00E-4
SNB-19	0.758	2.440	2.459	2.358	1.588	1.499	1.405	101	95	49	44	38	9.67E-7	> 1.00E-4	> 1.00E-4
SNB-75	0.792	1.717	1.492	1.469	0.807	0.871	0.886	76	73	2	8	10	2.11E-7	> 1.00E-4	> 1.00E-4
U251	0.534	2.297	2.350	2.294	1.213	1.042	0.908	103	100	39	29	21	6.50E-7	> 1.00E-4	> 1.00E-4
Melanoma															
LOX IMVI	0.364	2.745	2.641	2.395	1.248	1.239	0.996	96	85	37	37	27	5.40E-7	> 1.00E-4	> 1.00E-4
MALME-3M	0.701	1.129	1.099	1.069	0.922	0.983	0.948	93	86	52	66	58	> 1.00E-4	> 1.00E-4	> 1.00E-4
M14	0.514	1.911	1.825	1.540	0.706	0.599	0.646	94	73	14	6	9	2.47E-7	> 1.00E-4	> 1.00E-4
MDA-MB-435	0.454	2.605	2.370	0.753	0.316	0.212	0.252	89	14	-31	-53	-44	3.31E-8	2.06E-7	.
SK-MEL-2	1.090	2.527	2.494	2.387	1.840	1.796	1.927	98	90	52	49	58	.	> 1.00E-4	> 1.00E-4
SK-MEL-28	0.836	2.221	2.096	1.947	1.595	1.538	1.406	91	80	55	51	41	1.18E-5	> 1.00E-4	> 1.00E-4
SK-MEL-5	0.816	3.253	3.213	3.046	1.462	1.638	1.317	98	91	26	34	21	4.35E-7	> 1.00E-4	> 1.00E-4
UACC-257	1.087	2.259	2.238	2.229	1.883	2.089	2.123	98	97	68	85	88	> 1.00E-4	> 1.00E-4	> 1.00E-4
UACC-62	0.929	2.843	2.753	2.551	1.851	1.639	1.636	95	85	48	37	37	8.89E-7	> 1.00E-4	> 1.00E-4
Ovarian Cancer															
IGROV1	0.481	1.947	1.985	1.815	1.165	1.007	0.937	103	91	47	36	31	8.40E-7	> 1.00E-4	> 1.00E-4
OVCAR-3	0.459	1.894	1.923	1.800	0.678	0.486	0.466	102	93	15	2	.	3.60E-7	> 1.00E-4	> 1.00E-4
OVCAR-4	0.710	1.563	1.518	1.544	1.163	1.177	1.133	95	98	53	55	50	8.27E-5	> 1.00E-4	> 1.00E-4
OVCAR-5	0.789	1.861	1.719	1.746	1.456	1.185	1.118	87	89	62	37	31	3.04E-6	> 1.00E-4	> 1.00E-4
OVCAR-8	0.593	2.546	2.569	2.552	1.647	1.348	1.319	101	100	54	39	37	1.81E-6	> 1.00E-4	> 1.00E-4
NCI/ADR-RES	0.529	1.976	1.913	1.704	0.868	0.652	0.605	96	81	23	8	5	3.47E-7	> 1.00E-4	> 1.00E-4
SK-OV-3	1.180	2.523	2.406	2.439	1.692	1.665	1.551	91	94	38	36	28	6.11E-7	> 1.00E-4	> 1.00E-4
Renal Cancer															
786-0	0.745	2.662	2.500	2.566	1.667	1.490	1.341	92	95	48	39	31	9.10E-7	> 1.00E-4	> 1.00E-4
A498	1.461	2.138	1.986	2.134	1.541	1.391	1.349	78	99	12	-5	-8	3.66E-7	5.13E-6	> 1.00E-4
ACHN	0.385	1.621	1.583	1.620	0.947	0.915	0.799	97	100	45	43	33	8.24E-7	> 1.00E-4	> 1.00E-4
RXF 393	0.888	1.526	1.524	1.508	1.081	1.023	0.971	100	97	30	21	13	5.06E-7	> 1.00E-4	> 1.00E-4
SN12C	0.554	2.192	2.159	2.180	1.323	1.241	1.135	98	99	47	42	35	8.73E-7	> 1.00E-4	> 1.00E-4
TK-10	0.939	1.885	1.867	2.037	1.917	1.544	1.582	98	116	103	64	68	> 1.00E-4	> 1.00E-4	> 1.00E-4
UO-31	0.610	1.803	1.622	1.563	1.245	1.166	1.183	85	80	53	47	48	3.07E-6	> 1.00E-4	> 1.00E-4
Prostate Cancer															
PC-3	0.671	2.758	2.734	2.550	1.233	1.135	1.058	99	90	27	22	19	4.31E-7	> 1.00E-4	> 1.00E-4
DU-145	0.393	1.901	1.902	1.839	0.931	0.813	0.718	100	96	36	28	22	5.78E-7	> 1.00E-4	> 1.00E-4
Breast Cancer															
MCF7	0.440	2.382	2.091	1.988	0.909	0.662	0.752	85	80	24	11	16	3.43E-7	> 1.00E-4	> 1.00E-4
MDA-MB-231/ATCC	0.663	2.062	2.110	1.975	1.491	1.296	1.196	103	94	59	45	38	4.56E-6	> 1.00E-4	> 1.00E-4
HS 578T	1.019	1.979	1.957	1.895	1.343	1.297	1.240	98	91	34	29	23	5.21E-7	> 1.00E-4	> 1.00E-4
BT-549	1.227	2.158	1.994	2.042	1.568	1.504	1.473	82	88	37	30	26	5.46E-7	> 1.00E-4	> 1.00E-4
T-47D	0.976	2.085	1.924	1.994	1.817	1.430	1.579	85	92	76	41	54	.	> 1.00E-4	> 1.00E-4
MDA-MB-468	0.982	1.890	1.828	1.833	1.652	1.136	1.117	93	94	74	17	15	2.62E-6	> 1.00E-4	> 1.00E-4

National Cancer Institute Developmental Therapeutics Program<br>Mean Graphs	NSC : D - 795055/1	Units :Molar	SSPL :0GYG	EXP. ID :1701NS47
	Report Date :May 18, 2018		Test Date :January 30, 2017	

Panel/Cell Line	Log$_{10}$GI50	Log$_{10}$TGI	Log$_{10}$LC50
**Leukemia**			
CCRF-CEM	-6.39	> -4.00	> -4.00
HL-60(TB)	-6.64	-6.19	> -4.00
K-562	-7.04	> -4.00	> -4.00
MOLT-4	-6.19	> -4.00	> -4.00
RPMI-8226	-6.33	> -4.00	> -4.00
SR	-7.07	> -4.00	> -4.00
**Non-Small Cell Lung Cancer**			
A549/ATCC	-5.95	> -4.00	> -4.00
EKVX	-6.11	> -4.00	> -4.00
HOP-92	> -4.00	> -4.00	> -4.00
NCI-H226	> -4.00	> -4.00	> -4.00
NCI-H23	-6.04	> -4.00	> -4.00
NCI-H322M	-6.06	> -4.00	> -4.00
NCI-H460	-6.38	> -4.00	> -4.00
NCI-H522	-6.46	> -4.00	> -4.00
**Colon Cancer**			
COLO 205	-6.47	> -4.00	> -4.00
HCC-2998	-5.59	> -4.00	> -4.00
HCT-116	-6.37	> -4.00	> -4.00
HCT-15	-6.65	> -4.00	> -4.00
HT29	-6.49	> -4.00	> -4.00
KM12	-6.33	> -4.00	> -4.00
SW-620	-6.45	> -4.00	> -4.00
**CNS Cancer**			
SF-268	> -4.00	> -4.00	> -4.00
SF-295	-6.44	> -4.00	> -4.00
SF-539	-6.51	-5.53	> -4.00
SNB-19	-6.01	> -4.00	> -4.00
SNB-75	-6.68	> -4.00	> -4.00
U251	-6.19	> -4.00	> -4.00
**Melanoma**			
LOX IMVI	-6.27	> -4.00	> -4.00
MALME-3M	> -4.00	> -4.00	> -4.00
M14	-6.61	> -4.00	> -4.00
MDA-MB-435	-7.48	-6.69	
SK-MEL-2		> -4.00	> -4.00
SK-MEL-28	-4.93	> -4.00	> -4.00
SK-MEL-5	-6.36	> -4.00	> -4.00
UACC-257	> -4.00	> -4.00	> -4.00
UACC-62	-6.05	> -4.00	> -4.00
**Ovarian Cancer**			
IGROV1	-6.08	> -4.00	> -4.00
OVCAR-3	-6.44	> -4.00	> -4.00
OVCAR-4	-4.08	> -4.00	> -4.00
OVCAR-5	-5.52	> -4.00	> -4.00
OVCAR-8	-5.74	> -4.00	> -4.00
NCI/ADR-RES	-6.46	> -4.00	> -4.00
SK-OV-3	-6.21	> -4.00	> -4.00
**Renal Cancer**			
786-0	-6.04	> -4.00	> -4.00
A498	-6.44	-5.29	> -4.00
ACHN	-6.08	> -4.00	> -4.00
RXF 393	-6.30	> -4.00	> -4.00
SN12C	-6.06	> -4.00	> -4.00
TK-10	> -4.00	> -4.00	> -4.00
UO-31	-5.51	> -4.00	> -4.00
**Prostate Cancer**			
PC-3	-6.37	> -4.00	> -4.00
DU-145	-6.24	> -4.00	> -4.00
**Breast Cancer**			
MCF7	-6.47	> -4.00	> -4.00
MDA-MB-231/ATCC	-5.34	> -4.00	> -4.00
HS 578T	-6.28	> -4.00	> -4.00
BT-549	-6.26	> -4.00	> -4.00
T-47D		> -4.00	> -4.00
MDA-MB-468	-5.58	> -4.00	> -4.00
_MID	-5.96	-4.13	-4.0
Delta	1.52	2.56	-0
Range	3.48	2.69	0.0

GI50

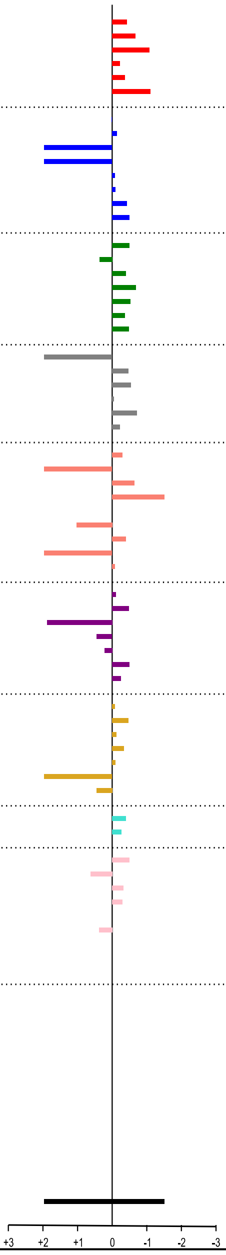

TGI

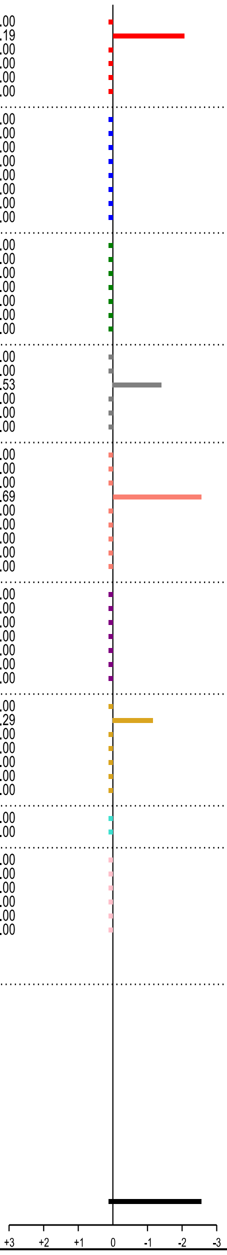

LC50

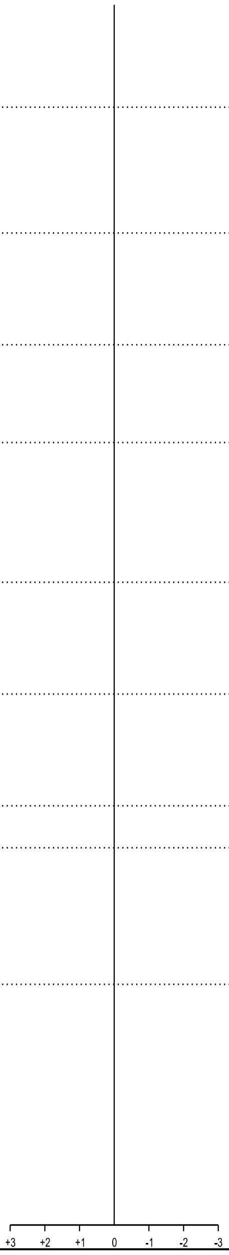

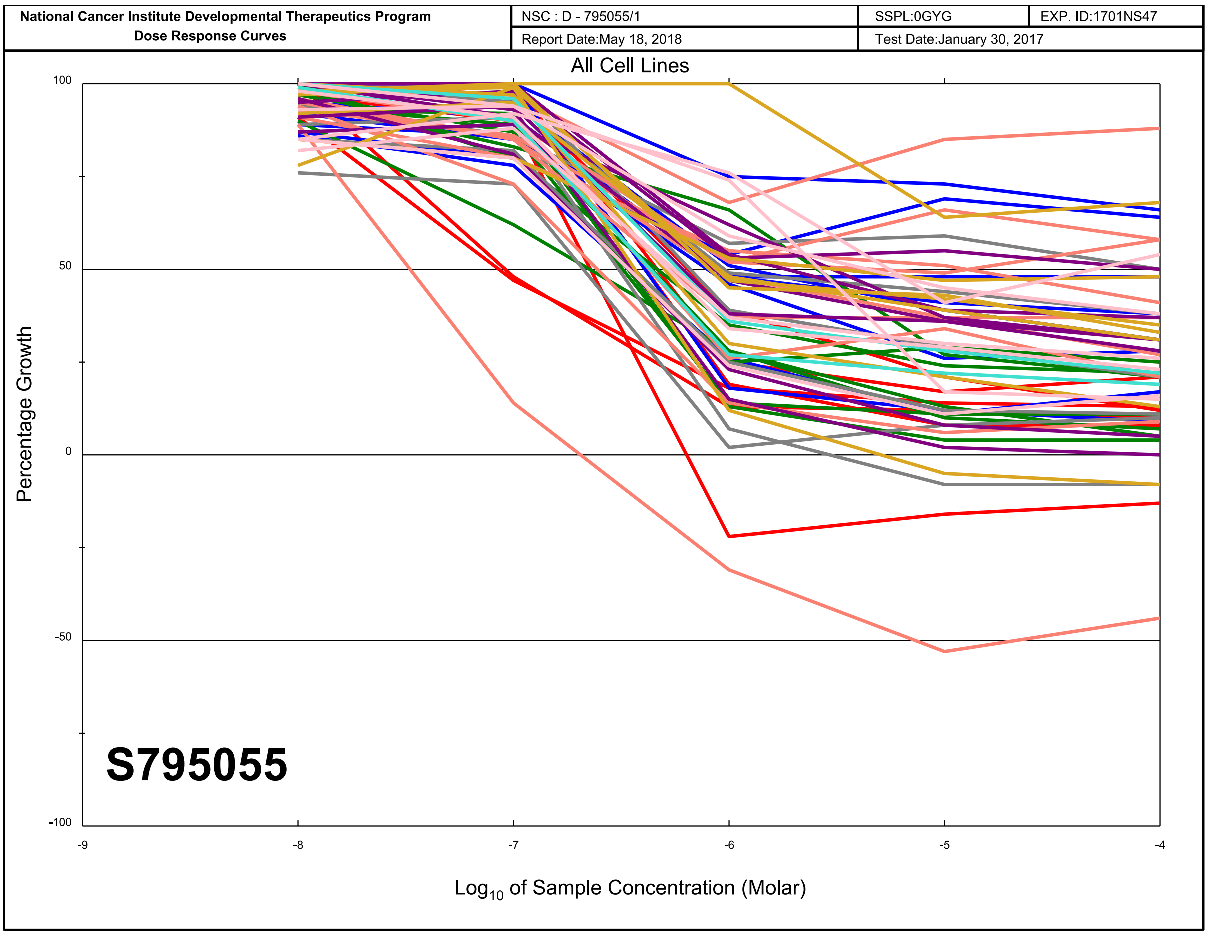
National Cancer Institute Developmental Therapeutics Program
Dose Response Curves
NSC : D - 795055/1
SSPL:0GYG
EXP. ID:1701NS47
Report Date:May 18, 2018
Test Date:January 30, 2017
All Cell Lines
Percentage Growth
100
50
0
-50
-100
-9
-8
-7
-6
-5
-4
Log10 of Sample Concentration (Molar)
S795055

Figure S25. Superposition of top-scoring computationally docked (cyan) and crystallographic pose (grey) of colchicine in tubulin: (a) overview and (b) detail.

(a)

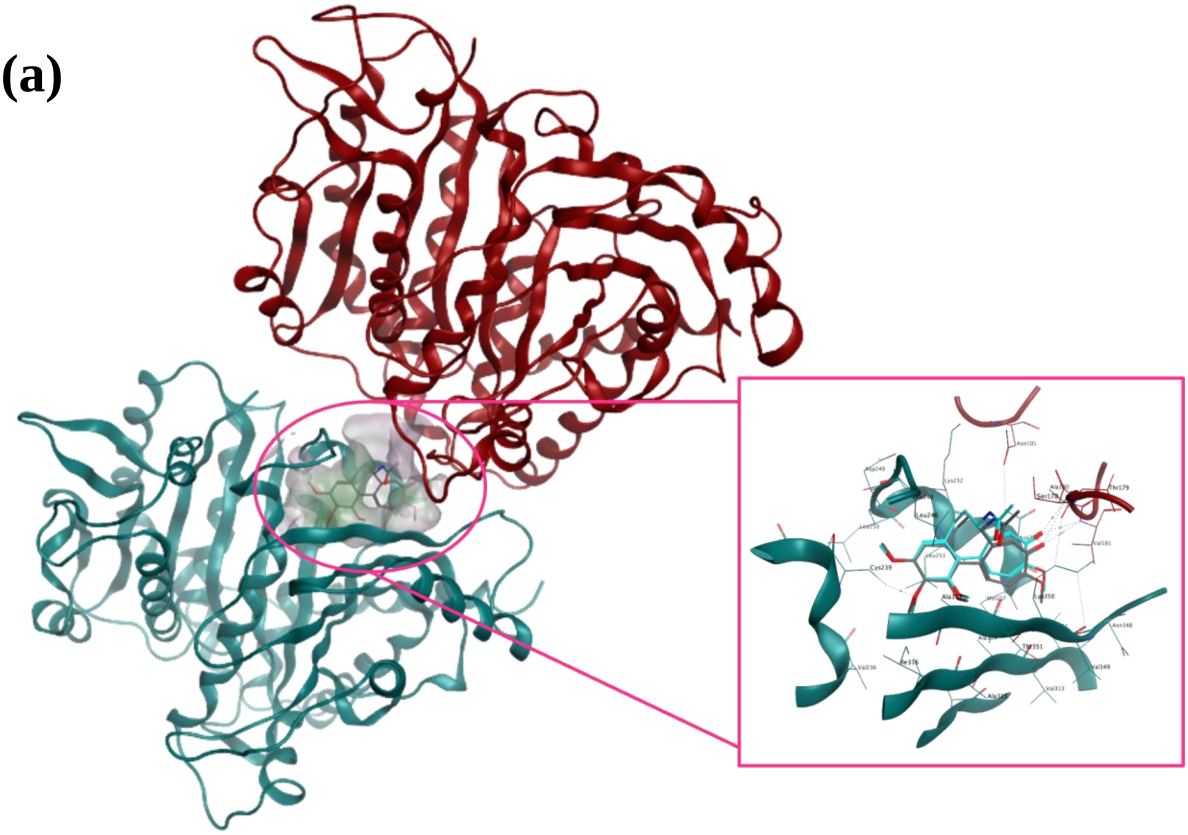

(b)

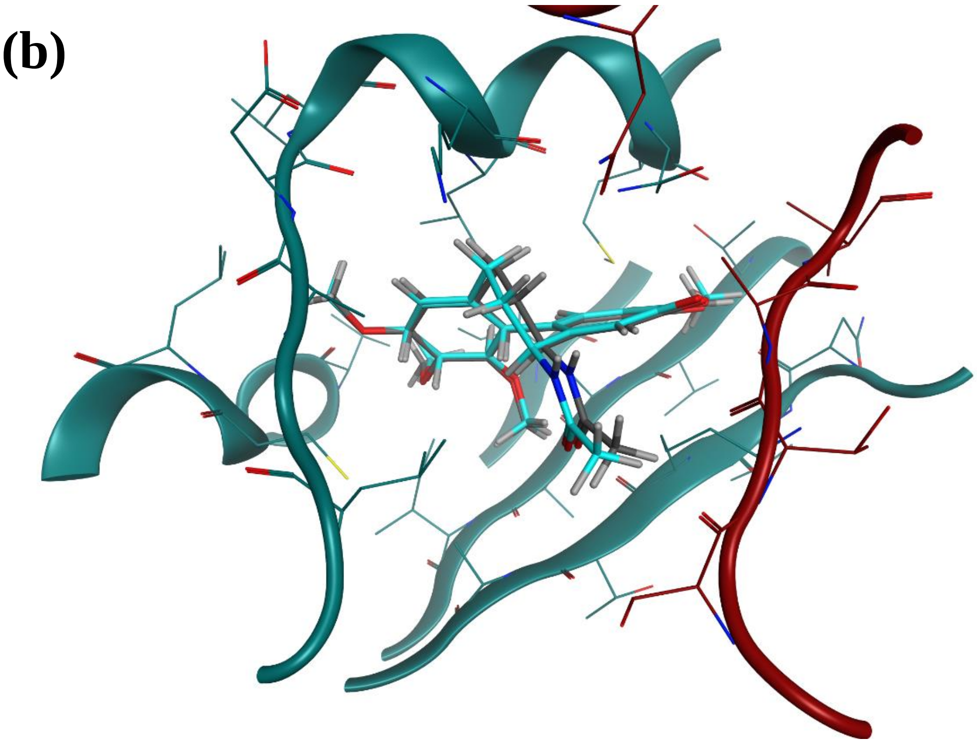

Figure S26. Gating of cells for cell cycle analysis by flow cytometry.

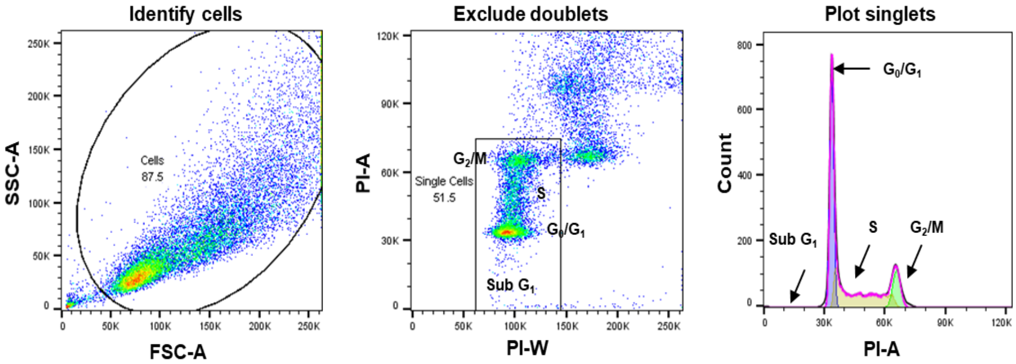

Samples were run at a low-flow rate through FACSCanto II Flow Cytometer (BD Biosciences, Flow Cytometry Facility, The University of Manchester) equipped with the FACSDiva software (v8.0.1). Propidium iodide (PI) was excited by the blue 488 nm laser and the signals were collected into Phycoerythrin (PE) detection channel. The area (A), height (H) and width (W) parameters for both the PI channel and light scatters were recorded. Any cell debris or cell fragments were gated out using forward scatter versus side scatter plot (FSC-A/SSC-A). Next, cell doublets and aggregates were excluded using the PI fluorescence plot (PI-A/PI-W) as the doublets are known to have greater pulse width and area than a single cell. The PI fluorescence data (PI-A) of 10,000 single cells were then acquired with linear amplification and plotted on a histogram. Further analysis was carried out using FlowJo_V10 software. The univariate Watson model was applied in an attempt to fit Gaussian curves to each phase, and thereby it was able to compute cells distribution in each cell cycle phase.

Figure S27. Gating and quadrant setting for Annexin-V/PI apoptosis assay.

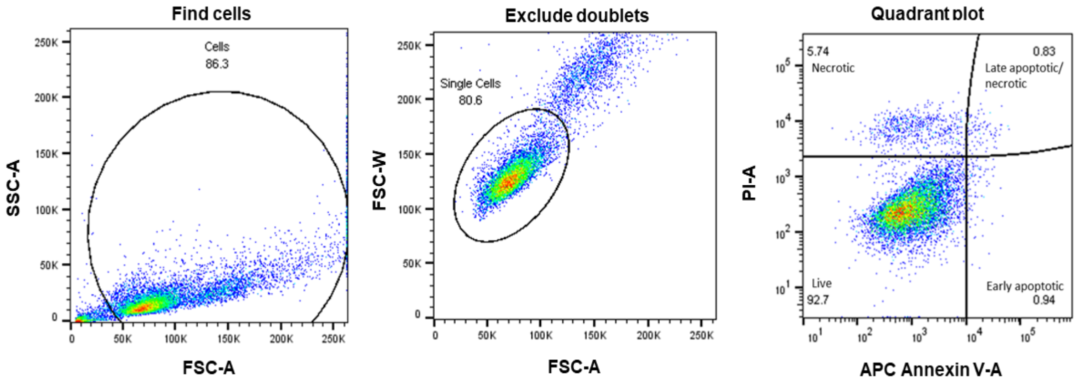

The samples were analysed quickly (i.e., within one hour from preparation) using the flow cytometer. The cells were gated in FSC-A/SSC-A and FSC-A/FSC-W windows to exclude cell debris and aggregates. Then, the data were collected from 10,000 single events. The data were plotted onto a quadrant graph using FlowJo_V10 software. The negative control was used to set the quadrant plot, whereby the percentages of different cell populations (live, early and late apoptotic and necrotic) could be determined.

Table S1. The COMPARE analysis results for **4** against the Standard NCI Database (Top 5 results).

COMPARE Solution	NSC Number	Mechanism of action	Correlation Coefficient	Reference
**1**	S-trityl-L-cysteine (S83265)	Kinesin Eg5 inhibitor	0.583	[3]
**2**	Rhizoxin (S332598)	Microtubule destabilisation	0.573	[4]
**3**	Paclitaxel (S125973)	Microtubule stabilisation	0.555	[4]
**4**	Methotrexate (S795055)	Dihydrofolate reductase inhibitor	0.545	[5]
**5**	Brequinar (S368390)	Dihydroorotate dehydrogenase inhibitor	0.480	[6]

Table S2. Docking scores for compounds to tubulin using the FRED docking software with the Chemgauss4 scoring function.

compound	R	R'	docking score
**Colchicine**	-	-	-15.2
**2**	6-F	m-OMe	-11.6
**3**	6-F	p-OMe	-11.0
**4**	6-F	m-F	-11.3
**5**	6-F	p-F	-10.9
**6**	7-F	m-OMe	-11.6
**7**	7-F	p-OMe	-10.5
**8**	7-F	p-F	-9.5

Table S3. Average distance dXO and standard deviation (in Å) between ligand atom X=F,Cl and tubulin backbone O atom of Val238.

compound	d_{XO}	
**4**	5.25	± 0.47
**5**	3.33	± 0.41
**8**	3.24	± 0.37
**md3**	4.09	± 0.96
**md4**	5.33	± 0.52
**md5**	5.59	± 0.44

Table S4. Final structure from MD simulation of ligand-tubulin complexes in explicit aqueous solvent. Surface of site shown in grey.

Mol	MD pose	Mol	MD pose
2	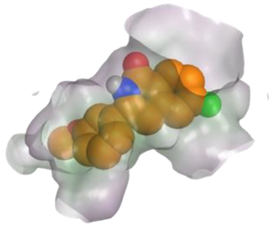	md1	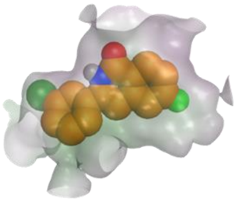
3	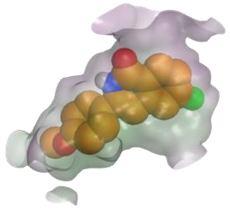	md2	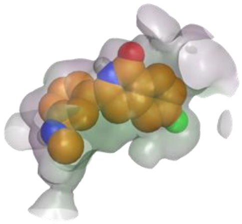
4	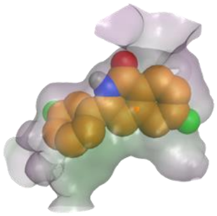	md3	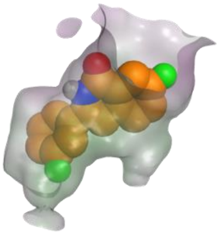
5	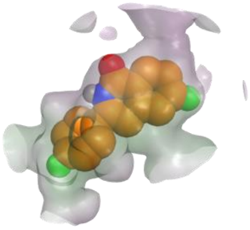	md4	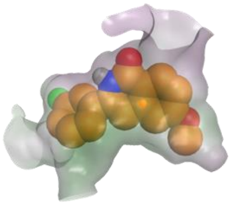
6	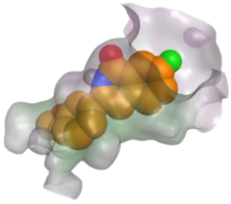	md5	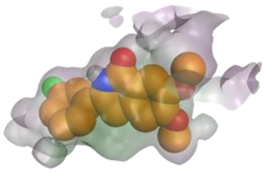
7	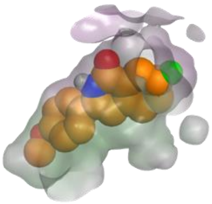	md6	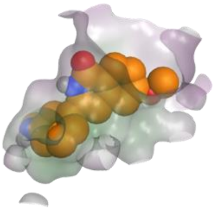

**8**	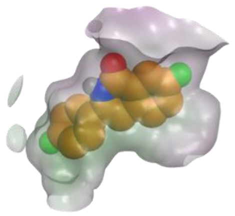		

Table S5. HPLC-MS purity analysis of compounds **2**, **3**, **4**, **5**, **6** and **7**

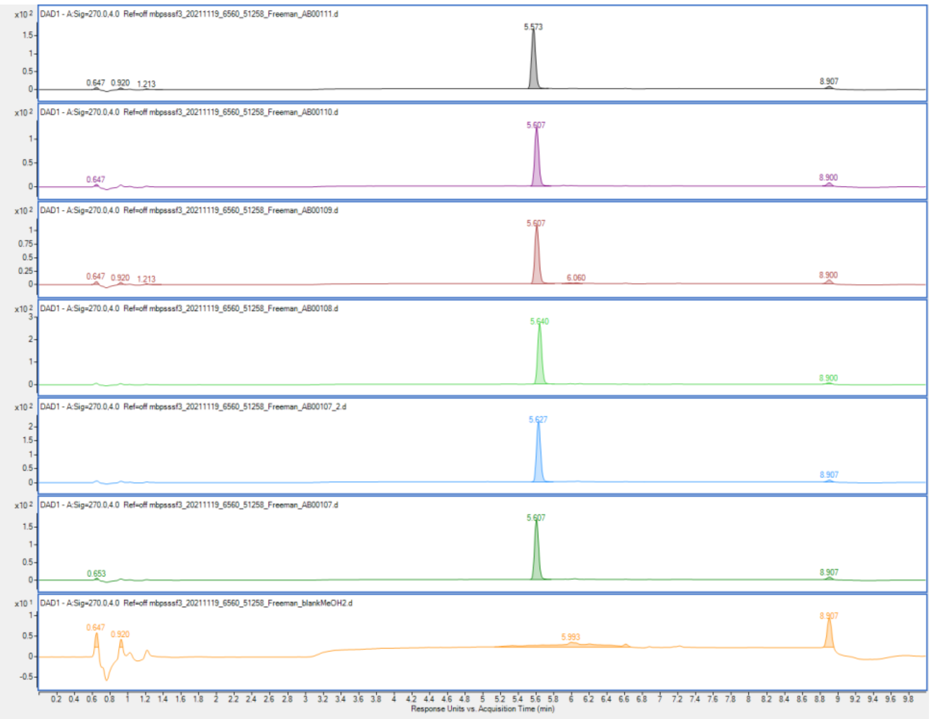

No 7

No 6

No 3

No 2

No 4

No 5

BLANK

Table S6. Molecular Formula Strings of compounds **2-8**

2: O=C1C2=CC=C(F)C=C2C=C(C3=CC=CC(OC)=C3)N1

3: O=C1C2=CC=C(F)C=C2C=C(C3=CC=C(OC)C=C3)N1

4: O=C1C2=CC=C(F)C=C2C=C(C3=CC=CC(F)=C3)N1

5: O=C1C2=CC=C(F)C=C2C=C(C3=CC=C(F)C=C3)N1

6: O=C1C2=CC(F)=CC=C2C=C(C3=CC=CC(OC)=C3)N1

7: O=C1C2=CC(F)=CC=C2C=C(C3=CC=C(OC)C=C3)N1

8: O=C1C2=CC(F)=CC=C2C=C(C3=CC=C(F)C=C3)N1

References

1. NCI 60 Cell One-Dose Screen. https://dtp.cancer.gov/discovery_development/nci-60/single_dose.htm (accessed 2019-11-04).

2. COMPARE Analysis. https://dtp.cancer.gov/databases_tools/docs/compare/compare_methodology.htm (accessed 2019-11-05).

3. Skoufias, D. A.; DeBonis, S.; Saoudi, Y.; Lebeau, L.; Crevel, I.; Cross, R.; Wade, R. H.; Hackney, D.; Kozielski, F. J. J. o. b. c. S-trityl-L-cysteine is a reversible, tight binding inhibitor of the human kinesin Eg5 that specifically blocks mitotic progression. *J Biol Chem* **2006,** 281, 17559-17569.

4. Stanton, R. A.; Gernert, K. M.; Nettles, J. H.; Aneja, R. Drugs that target dynamic microtubules: a new molecular perspective. *Med Res Rev* **2011,** 31, 443-81.

5. Schweitzer, B. I.; Dicker, A. P.; Bertino, J. R. Dihydrofolate reductase as a therapeutic target. *FASEB J* **1990,** 4, 2441-52.

6. Peters, G. J.; Kraal, I.; Pinedo, H. M. In vitro and in vivo studies on the combination of Brequinar sodium (DUP-785; NSC 368390) with 5-fluorouracil; effects of uridine. *Br J Cancer* **1992,** 65, 229-233.
